# Biologicals for the treatment of lupus nephritis: a Bayesian network meta-regression analysis

**DOI:** 10.3389/fimmu.2024.1445814

**Published:** 2024-08-30

**Authors:** Xi Liu, Xiaoli Chen, Chengyin Yang, Ruixue Li, Xi Chen, Qiaoli Li

**Affiliations:** ^1^ Nephrology Department, The People’s Hospital of Yubei District of Chongqing, Chongqing, China; ^2^ Zhejiang University, Department of Epidemiology and Statistics, School of Public Health, Medical College, Hangzhou, Zhejiang, China; ^3^ Stomatology Department, The Thirteenth People’s Hospital of Chongqing, Chongqing, China

**Keywords:** biological agents, lupus nephritis, follow-up period, Bayesian network meta-regression analysis, complete response rate

## Abstract

**Background:**

Previous studies comparing the efficacy and safety of different treatment regimens for lupus nephritis are scarce. Moreover, confounding factors such as the duration of follow-up were hardly adjusted in those studies, potentially compromising the results and their extents to clinical settings.

**Objective:**

To rigorously investigate the efficacy and safety of biologics in patients with lupus nephritis using Bayesian network meta-regression analyses that adjust for the follow-up period, in order to provide more robust evidence for clinicians.

**Methods:**

Databases comprising PubMed, Embase, MedlinePlus, Cochrane Library, Google Scholars, and Scopus were retrieved for eligible articles from inception to February 29, 2024. The primary endpoint was the complete response rate, the secondary endpoint was the partial response rate, the tertiary endpoints were the adverse events, and infection-related adverse events. Napierian Logarithm of hazard ratio (lnHR) and the standard error of lnHR (selnHR) were generated for dichotomous variants by STATA 18.0 MP and then put into Rstudio 4.3.2 to conduct Bayesian network meta-analysis as well as network meta-regression analysis to yield hazard ratio (HR) as pairwise effect size.

**Results:**

Ten studies involving 2138 patients and 11 treatment regimens were ultimately included. In the original analysis, for the primary endpoint, compared to the control group, obinutuzumab (22.6 months), abatacept-30mg (20.5 months), abatacept-10mg (17.8 months), and belimumab (23.3 months) demonstrated significant superiority (HR ranged from 1.6 to 2.5), more ever, their significance regarding relative efficacy was correlated with follow up period, namely “time window” (shown in parentheses above). For the secondary endpoint, compared to the control group, obinutuzumab and abatacept-30mg showed conspicuous preponderance (HR ranged from 1.6 to 2.4), “time window” was also detected in abatacept-30mg (20.5 months), whereas obinutuzumab remained consistently obviously effective regardless of the follow-up period (shown in parentheses above). For the tertiary endpoint, there were no differences among active regimens and control.

**Conclusions:**

Considering the efficacy and safety and “time window” phenomenon, we recommend obinutuzumab as the preferred treatment for LN. Certainly, more rigorous head-to-head clinical trials are warranted to validate those findings.

## Introduction

Systemic lupus erythematosus (SLE), characterized by the presence of autoantibodies, is a multifactorial autoimmune disease impairing one or more organs and has a wide range of clinical symptoms (1). Lupus nephritis (LN) is a prevalent and severe manifestation of systemic lupus erythematosus (SLE), involving kidneys. The estimated prevalence of lupus nephritis among lupus patients ranges from approximately 35% to 60%, with variations based on factors such as age, gender, and race, mainly affecting young women (2–4). SLE patients with LN undergo significantly increased risks of myocardial infarction, cardiovascular mortality, and progression to end-stage renal disease within 5 years, which further elevates the risk of death compared to the SLE patients without LN (5–7).

Currently, the predominant treatment approach for LN involves a combination of corticosteroid therapy and immunosuppressants. However, this approach is susceptible to relapse and can cause organ damage during treatment (8, 9). Although our understanding of the pathogenesis of LN has evolved and novel treatment panels have been developed, only 50%—70% of patients showed better outcomes, and LN continues to be the major cause of onset and death in SLE patients (10). Consequently, innovating new drugs with reduced side effects and enhanced efficacy remains the overwhelming task of LN.

Luckily, a number of biologicals have been created and validated in recent years for the management of LN, including abatacept, anifrolumab, belimumab, interleukin-2 (IL-2), obinutuzumab, ocrelizumab and rituximab. Certainly, clinicians were ecstatic to witness so many blooming biologicals. However, they were obsessed in a dilemma on making choice since there lacked direct comparisons among those biologicals in terms of efficacy and safety.

Previous network meta-analyses ubiquitously lacked the adjustment for follow-up time, and its common sense that the follow-up period could influence the evaluation of treatment outcomes, therefore those studies might suffer a shortfall in their applicability to clinical settings for their results (11).

Hence, we conducted this Bayesian network meta-regression analysis to update and expand upon previous meta-analyses, comparing various treatments of LN and additionally addressing the potential impact of follow-up time to provide more pragmatic evidence for clinical decision-making.

## Methods

### Search strategy

Databases including PubMed, Embase, MedlinePlus, Cochrane Library, Google Scholar, and Scopus were searched for eligible articles from their inception to February 29, 2024. The literature search was conducted using the following MeSH terms: ‘Lupus Erythematosus, Systemic’ OR ‘Lupus Nephritis’ OR ‘Abatacept’ OR ‘Anifrolumab’ OR ‘Belimumab’ OR ‘Interleukin-2’ OR ‘Obinutuzumab’ OR ‘Ocrelizumab’ OR ‘Rituximab’ OR ‘Biological Therapy’ OR ‘Randomized Controlled Trials as Topic’.

### Selection criteria

Inclusion Criteria:

1. Randomized clinical trials (RCTs) of biological agents for the treatment of LN.
2. Adult patients meeting the SLE diagnostic criteria based on the 1982 American College of Rheumatology SLE classification criteria updated in 1997, shown in **Supplementary Data 1**.
3. The experimental group received standard combination therapy for SLE.

Exclusion Criteria:

1. During the induction and maintenance treatment phases, different biologic agents were used, or only one phase involved the use of biologic agents.
2. Studies with incomplete or ambiguous data on outcome indicators.
3. Full text that was not accessible.
4. Reviews, conference proceedings, or duplicate publications.

### Endpoints

Primary Endpoint: Evaluable effectiveness indicators include the complete response rate (CRR), a composite measure requiring a UPCR < 0.5, normal renal function (serum creatinine ≤ upper limit of normal), serum creatinine increase not exceeding 15% from baseline, and < 10 red blood cells (RBCs) per high-power field in urine sediment without RBC casts.

Secondary Endpoint: Partial response rate (PRR) is another composite measure requiring a ≥ 50% reduction in UPCR to < 1 (or < 3 if baseline UPCR was ≥ 3), serum creatinine increase not exceeding 15% from baseline, and < 10 RBCs/HPF or ≤ 50% increase over baseline in urinary RBCs.

Tertiary Endpoint: Safety indicators include adverse events (AE) and infection-related adverse events (IAE).

### Data extraction and quality assessment

Two researchers (XL and XC) independently extracted the included studies, and any disagreements were arbitrated by a senior evaluator (XC). The following information was collected: Name of the first author, ethnicity, publication year, sample size, age, intervention, follow-up period, number of CRR, number of PRR, number of AE, number of IAE. The included RCTs’ bias risks were evaluated referring to the Risk of Bias tool, 2nd edition (RoB2) by Review Manager 5.3.

### Statistical analysis

Using STATA 18.0 MP, the Napierian Logarithm of hazard ratio (lnHR) and the standard error of lnHR (selnHR) were calculated for binomial variants, namely CRR, PRR, AE, and IAE, in each study. Subsequently, these data were input into Rstudio 4.3.2 for Bayesian network meta-analysis followed by network meta-regression using the “gemtc” package. Inconsistencies were assessed through inconsistency and consistency tests. If I^2^ < 50% and p > 0.01, a fixed-effect model would be implemented; if 50% < I^2^ < 75%, a random-effects model would be used; if I^2^ > 75%, a Galbraith plot would be formulated to eliminate heterogeneity. To obtain a posterior distribution, Markov chain Monte Carlo (MCMC) with 20,000 burn-ins and 300,000 iterations, repeated four times for each chain, were

used, with a thinning interval of 10 to generate hazard ratios (HRs) for pairwise comparisons. The Brooks-Gelman-Rubin diagnostics and trace and density plots were used to assess and visualize the convergence of the model. Furthermore, the surface under the cumulative ranking curve (SUCRA) and matrices were generated.

In the sensitivity analysis, specifically the Bayesian network meta-regression analyses, the follow-up period was enrolled as a co-variate to explore the correlation between effect size and it. We conducted pairwise comparisons between biological agents and the control group. In each pairwise comparison, we calculated the effect size (lnHR). Based on the follow-up time (time points) and the corresponding lnHR values, curves were drawn to represent the relative effects of each biological agent compared to other biological agents at different follow-up time points. The definite timing of the initiation and termination, namely the “time window”, was captured from the time-effect-size plots using Getdata 3.6. The statistical analysis has been reviewed by a professional statistician (XC).

## Results

### Basic characteristics and quality assessment

**Figure 1:**
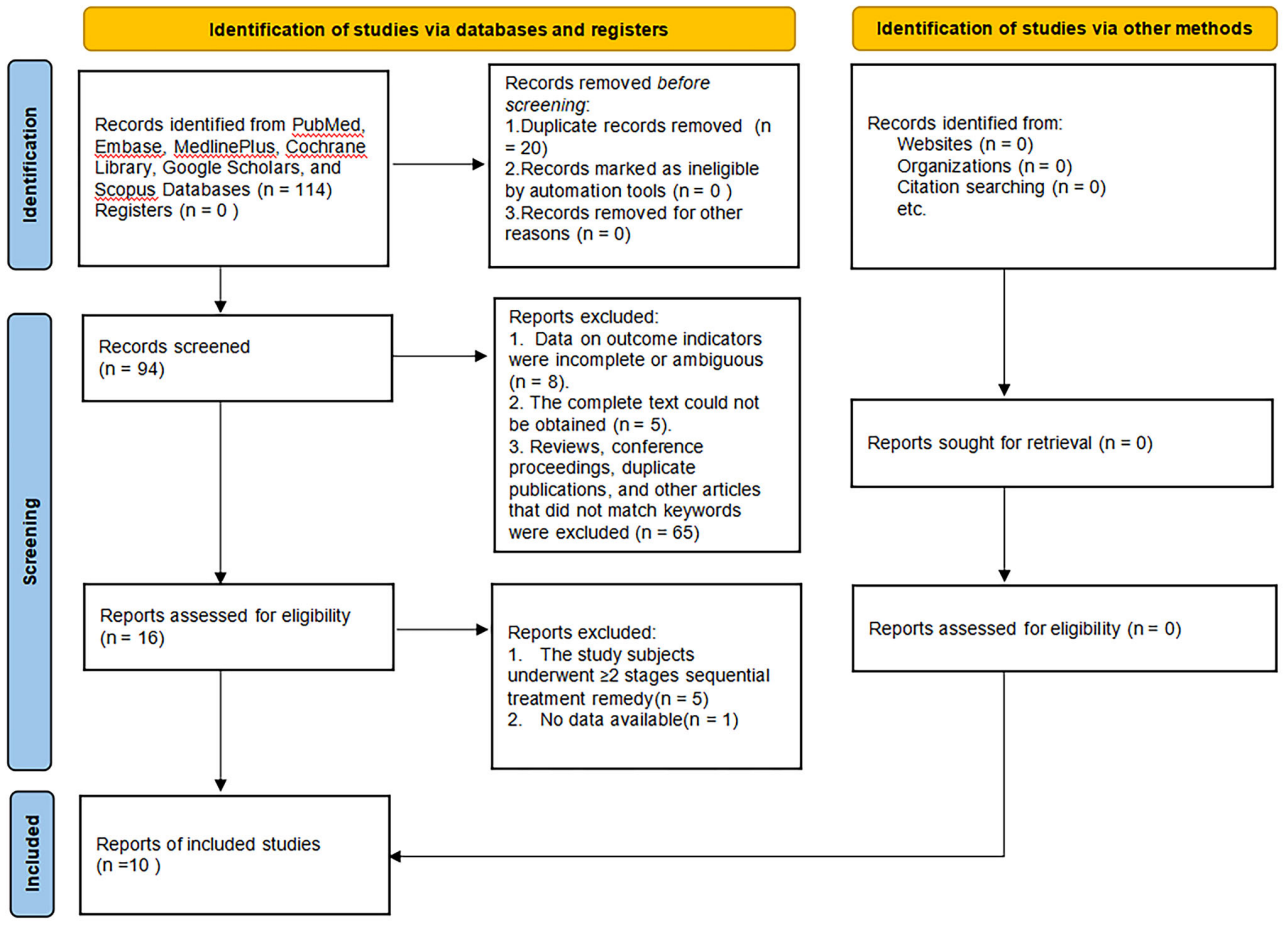
PRISMA flow chart.

**Figure 2:**
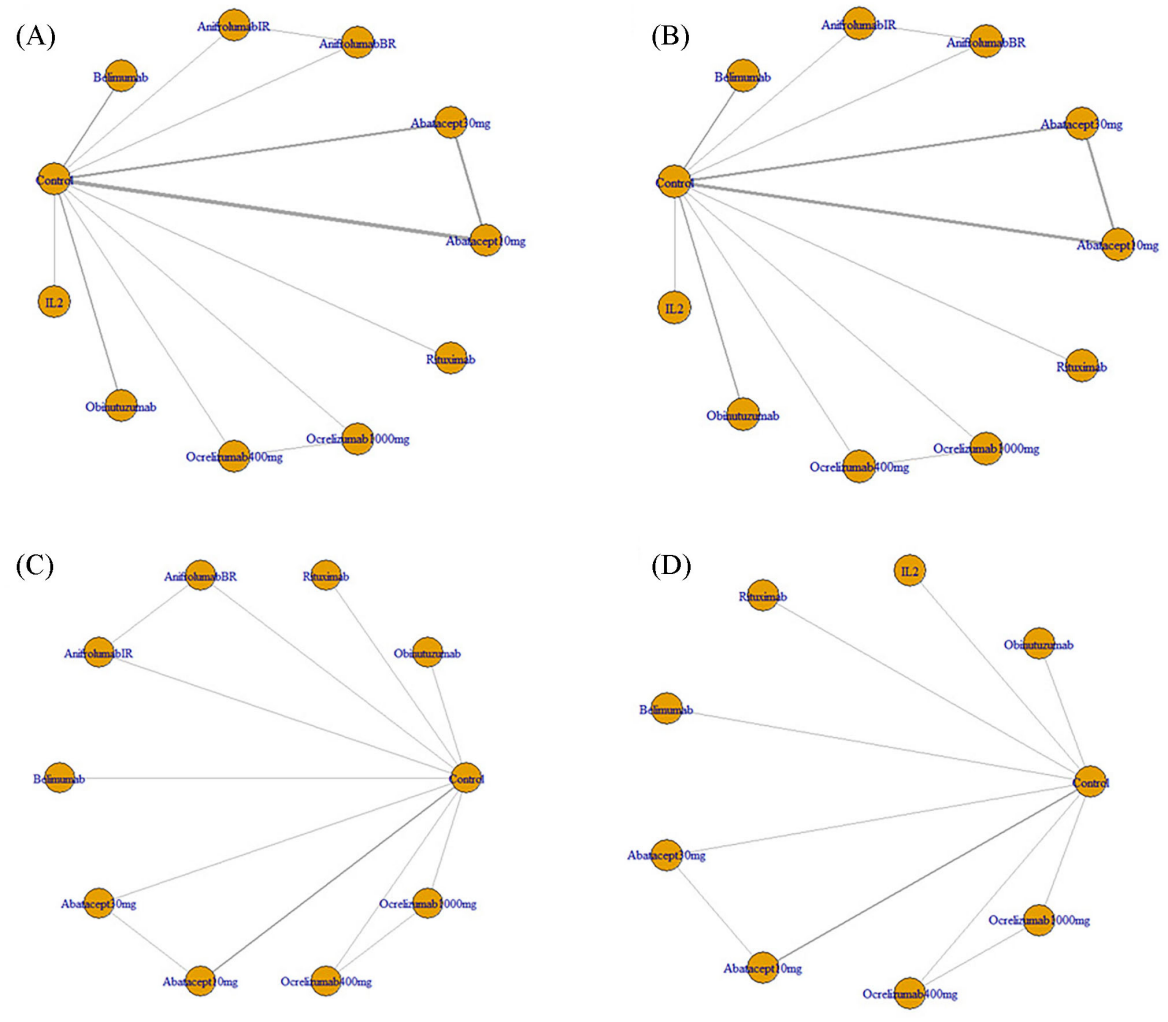
**(A)** Regression network plots of CRR outcomes for different biological agents in the treatment of LN; **(B)** Regression network plots of PRR outcomes for different biological agents in the treatment of LN; **(C)** Regression network plots of AE outcomes for different biological agents in the treatment of LN; **(D)** Regression network plots of IAE outcomes for different biological agents in the treatment of LN; CRR, Complete Response Rate; PRR, Partial Response Rate; AE, Adverse Events; IAE, Infection Adverse Events; LN, Lupus Nephritis; Abatacept10mg, Abatacept approximating 10 mg/kg on all infusion days; Abatacept30mg, Abatacept 30 mg/kg on days 1, 15, 29, and 57, followed by abatacept approximating 10 mg/kg (weight tiered, 500 mg for patients weighing 60 kg, 750 mg for patients 60-100 kg, 1,000 mg for patients > 100 kg) on days 85, 113, 141, 169, 197, 225, 253, 281, 309, and 337; AnifrolumabBR, Anifrolumab basic regimen (BR; 300 mg, corresponding to SLE dosing 10-12), AnifrolumabIR, Anifrolumab intensified regimen (IR; 900 mg for the first three doses, 300 mg thereafter); Ocrelizumab1000mg, 1,000 mg ocrelizumab given as an intravenous (IV) infusion on days 1 and 15, followed by a single infusion at week 16 and every 16 weeks thereafter; Ocrelizumab400mg, 400 mg ocrelizumab given as an intravenous (IV) infusion on days 1 and 15, followed by a single infusion at week 16 and every 16 weeks thereafter.

Eventually, 10 studies reporting on 11 regimens involving a total of 2138 patients were included after screening 2346 publications, removing duplicates, and filtering titles and abstracts from 144 studies that met the criteria for full review, a detailed explanation of the inclusion in the study can be found in **Figure 1**. The follow-up durations ranged from 24 to 104 weeks. The treatment regimens encompassed abatacept-10mg, abatacept-30mg, anifrolumab-BR, anifrolumab-IR, belimumab, IL-2, obinutuzumab, ocrelizumab-1000mg, ocrelizumab-400mg, and rituximab (12–21), as shown in **Table 1**. The regression network plots of CRR, PRR, AE, and IAE outcomes for different biological agents in the treatment of LN are shown in **Figure 2**. All studies were at low risk of bias, shown in **Supplementary Figure 1**.

**Table 1:** General characteristics of individual studies.

Study	Year	Ethnicity	LN biopsy classification^$$^	Age^||^ (years ± SD)	Treatment	Standard treatment	Sample size	Follow-up (month)	Dosage	Outcomes
Richard A Furie (12)	2022	Mixed^m^	III, IV, V	33.1 ± 9.8 31.9 ± 10.1	Obinutuzumab^a^ Control	MMF	63 62	52 weeks and 104 weeks	Obinutuzumab was administered as an intravenous infusion of 1,000 mg on day 1 and at weeks 2, 24, and 26.	CRR^i^、PRR^j^、AE^k^、IAE^l^
David Jayne (13)	2022	European	III, IV, V	34.0 (19-67) ^d^ 35.0 (18-65) 32.0 (18-58)	Anifrolumab-BR^b^ Anifrolumab-IR^c^ Control	MMF	45 51 49	52 weeks	Patients received the anifrolumab basic regimen (BR; 300 mg), the anifrolumab intensified regimen (IR; 900 mg for the first three doses, 300 mg thereafter), or placebo intravenously every 4 weeks for 48 weeks.	CRR、PRR、AE
L Andreoli (14)	2022	European	III, IV, V	36.1 ± 11.07 36.5 ± 10.90	Belimumab Control	CYC/AZA or MMF	41 45	104 weeks	Patients received intravenous belimumab at a dose of 10 mg per kilogram of body weight on days 1 (baseline), 15, and 29 and every 28 days thereafter to week 100.	CRR、PRR
Richard Furie (15)	2020	Mixed^n^	III, IV, V	33.7 ± 10.7 33.1 ± 10.6	Belimumab Control	CYC/AZA or MMF	224 224	104 weeks	Patients received intravenous belimumab at a dose of 10 mg per kilogram of body weight on days 1 (baseline), 15, and 29 and every 28 days thereafter to week 100.	CRR、PRR、AE、IAE
Jing He (16)	2019	Asian	clinical diagnosis LN	31.58 ± 9.25 29.83 ± 9.72	IL2 Control	CYC, MMF, or other immuno-suppressants	13 12	24 weeks	IL2 (1 million IU) or placebo was administered subcutaneously every other day for 2 weeks (seven injections), followed by a 2-week break, as one treatment cycle of 4 weeks.	CRR、PRR、IAE
Rovin H (21)	2012	Mixed°	III, IV	31.8 ± 9.6 29.4 ± 9.3	Rituximab Control	MMF	42 42	52 weeks	Rituximab (1,000 mg) on days 1, 15, 168, and 182.	CRR、PRR、AE、IAE
Richard Furie (17)	2014	Mixed^p^	III, IV, V	31.0 ± 9.5 30.5 ± 10.6 31.8 ± 9.0	Abatacept-30mg^e^ Abatacept-10mg ^f^ Control	MMF	99 99 100	104 weeks	Patients assigned to the abatacept 30/10 regimen received abatacept 30 mg/kg on days 1, 15, 29, and 57, followed by abatacept approximating 10 mg/kg (weight tiered: 500 mg for patients weighing 60 kg, 750 mg for patients 60–100 kg, 1,000 mg for patients 100 kg) on days 85, 113, 141, 169, 197, 225, 253, 281, 309, and 337. In the abatacept 10/10 group, patients received weight-tiered doses of abatacept approximating 10 mg/kg on all infusion days.	CRR、PRR、AE、IAE
David Wofsy (19)	2013	Not mentioned	III, IV, V	Not mentioned	Abatacept-30mg Abatacept-10mg Control	MMF	86 87 80	24 weeks and 52 weeks	An abatacept10mg group received abatacept infusions at a fixed, weight-tiered dose approximating 10 mg/kg according to the same schedule. The abatacept 30mg group received a higher dose of abatacept for the first 5 infusions (30 mg/kg), followed by a fixed, weight-tiered dose of ~10 mg/kg every 28 days.	CRR、PRR
Eduardo F (20)	2013	Mixed^q^	III, IV, V	31.9 (16–69)^d^ 30.6 (16–60) 31.3 (17–66)	Ocrelizumab-400mg^g^ Ocrelizumab-1000mg ^h^ Control	CYC/AZA or MMF	75 73 75	48 weeks	Patients received the placebo,400 mg ocrelizumab, or 1,000 mg ocrelizumab given as an intravenous (IV) infusion on days 1 and 15, followed by a single infusion at week 16 and every 16 weeks thereafter.	CRR、PRR、AE、IAE
ACCESS Trial Group (18)	2014	Mixed^r^	III, IV, V	32.0 ± 10.1 32.7 ± 12.0	Abatacept-10mg Control	CYC/AZA or MMF	66 68	24 weeks and 52 weeks	Treatment was initiated with monthly infusions of abatacept at doses (for <60 kg, 500 mg; for 60–100 kg, 750 mg; for 100 kg, 1 gm)	CRR、PRR、AE、IAE

LN, lupus nephritis; SD, standard deviation; IL-2, interleukin-2; CYC, cyclophosphamide; AZA, azathioprine; MMF, mycophenolate mofetil.

^$$^The classification of kidney biopsy is based on the criteria established by the International Society of Nephrology/Renal Pathology Society in 2003;

^||^If not specified, data were described as mean ± SD;

a Obinutuzumab was administered as a blinded intravenous infusion of 1,000 mg on day 1 and at week 2nd, 24th, and 26th;

b BR, basic regimen; Anifrolumab-BR, Anifrolumab 300 mg;

c IR, intensified regimen; Anifrolumab-BR, Anifrolumab 900 mg for the first three doses, 300 mg thereafter;

d Data were described as median (range);

e Abatacept 30 mg/kg on day 1st, 15th, 29th, and 57th, followed by abatacept approximating 10 mg/kg (weight tiered: 500 mg for patients weighing 60 kg, 750 mg for patients 60–100 kg, 1,000 mg for patients 100 kg) on day 85th, 113th, 141st, 169th, 197th, 225th, 253rd, 281st, 309th, and 337th;

f Abatacept approximating 10 mg/kg on all infusion days;

g 400 mg ocrelizumab given as an intravenous (IV) infusion on day 1st and 15th, followed by a single infusion at week 16th and every 16 weeks thereafter;

h 1,000 mg ocrelizumab given as an intravenous (IV) infusion on day 1st and 15th, followed by a single infusion at week 16th and every 16 weeks thereafter;

i CRR, complete renal response; UPCR <0.5 g/g, eGFR no more than 10% below preflare value or ≥90 ml/min/1.73 m^2^, and no treatment failure;

j PRR, partial renal response; composite measure requiring ≥50% reduction in UPCR from baseline to a value <1 (to <3 if baseline UPCR was ≥3), serum creatinine not increased >15% from baseline and urinary RBCs <10/HPF or ≤50% increase over the baseline value;

k AE, adverse event;

l IAE, infection adverse event;

m Latin America and Caribbean 60%, Europe and Israel 29%, the USA 11%;

n Asian 223 (50%), white 148 (33%), black 61 (14%), American Indian or Alaska Native 10 (2%), and multiple races 4 (1%);

°White 26.4%, black 27.8%, Hispanic 40.3%, Asian/Pacific Islander 5.6%;

p Asian 60.6%, white 28.3%, African American 6.1%, other 5.1%.

q Latin America 42%, Asia 23.1%, Western Europe 12.9%, Eastern Europe 10.5%, the USA and Canada 10.2%, Africa 1.3%.

r White 51%, African American 41%, Asian 3%, undeclared 3%.

### Primary endpoint

In the raw analysis: compared to the control group, abatacept-10mg (HR=1.62, 95% CI: 1.1 to 2.25), abatacept-30mg (HR=1.85, 95% CI: 1.18 to 3.08), and obinutuzumab (HR=2.11, 95% CI: 1.06 to 4.21) showed significance, while the rest did not demonstrate significance, as shown in **Supplementary Table 1**.

In the sensitivity analysis, belimumab (HR=2.4, 95% CI: 1.1 to 5.1) showed significance compared with the control group, different from the raw analysis. Other interventions showing significance included abatacept-10mg (HR=1.6, 95% CI: 1.1 to 2.5), abatacept-30mg (HR=1.9, 95% CI: 1.2 to 3.2), and obinutuzumab (HR=2.5, 95% CI: 1.2 to 5.3), resembling the raw analysis. Compared to obinutuzumab, abatacept-30mg (HR=0.66, 95% CI: 0.29 to 1.5), abatacept-30mg (HR=0.77, 95% CI: 0.33 to 1.8), and belimumab (HR=0.94, 95% CI: 0.37 to 2.4) did not show significant differences. Obinutuzumab was superior to anifrolumab-BR (HR=0.17, 95% CI: 0.052 to 0.57) and ocrelizumab-1000mg (HR=0.31, 95% CI: 0.10 to 0.89), as shown in **Table 2**.

**Table 2:** Matrix and SUCRA^a^ of pairwise comparisons of regimens on CRR^b^ after Bayesian network meta-regression (shown as HR^c^ and 95% Cls^d^).

	Abatacept-10mg^e^	Abatacept-30mg^f^	Anifrolumab-BR^g^	Anifrolumab-IR^h^	Belimumab
SUCRA (%)	58	70	3	54	78
Abatacept-10mg	Abatacept-10mg	1.2 (0.76, 1.8)	0.27(0.096, 0.72)	0.93 (0.35, 2.4)	1.4 (0.61, 3.3)
Abatacept-30mg	0.85 (0.55, 1.3)	Abatacept-30mg	0.23(0.078, 0.63)	0.79 (0.29, 2.1)	1.2 (0.51, 2.9)
Anifrolumab-BR	3.8 (1.4, 10.0)	4.4 (1.6, 13.0)	Anifrolumab-BR	3.5 (1.4, 8.7)	5.4 (1.6, 18.0)
Anifrolumab-IR	1.1 (0.42, 2.8)	1.3 (0.48, 3.4)	0.29 (0.11, 0.71)	Anifrolumab-IR	1.6 (0.49, 5.0)
Belimumab	0.69 (0.30, 1.7)	0.82 (0.35, 2.0)	0.18(0.054, 0.62)	0.65 (0.20, 2.1)	Belimumab
Control	1.6 (1.1, 2.5)	1.9 (1.2, 3.2)	0.44 (0.17, 1.1)	1.5 (0.65, 3.6)	2.4 (1.1, 5.1)
IL2	0.40 (0.051, 3.2)	0.47 (0.059, 3.9)	0.11(0.012, 0.97)	0.37 (0.043, 3.3)	0.58 (0.061, 5.6)
Obinutuzumab	0.66 (0.29, 1.5)	0.77 (0.33, 1.8)	0.17(0.052, 0.57)	0.61 (0.19, 1.9)	0.94 (0.37, 2.4)
Ocrelizumab-1000mg	2.1 (0.93, 5.0)	2.5 (1.1, 6.1)	0.56 (0.17, 1.8)	2.0 (0.65, 6.0)	3.0 (1.0, 9.3)
Ocrelizumab-400mg	1.2 (0.55, 2.9)	1.4 (0.62, 3.6)	0.33 (0.10, 1.1)	1.1 (0.38, 3.5)	1.8 (0.60, 5.4)
Rituximab	1.5 (0.58, 4.2)	1.8 (0.66, 5.1)	0.41 (0.11, 1.5)	1.4 (0.41, 4.9)	2.2 (0.66, 7.5)
Control	IL2	Obinutuzumab	Ocrelizumab-1000mg^i^	Ocrelizumab-400mg^j^	Rituximab
26	83	81	17	46	34
0.61 (0.40, 0.89)	2.5 (0.31, 19.)	1.5 (0.66, 3.5)	0.47 (0.20, 1.1)	0.81 (0.35, 1.8)	0.66 (0.24, 1.7)
0.52 (0.31, 0.81)	2.1 (0.26, 17.)	1.3 (0.54, 3.0)	0.40 (0.16, 0.95)	0.69 (0.28, 1.6)	0.56 (0.19, 1.5)
2.3 (0.91, 5.8)	9.4 (1.0, 85.0)	5.7 (1.7, 19.0)	1.8 (0.55, 5.8)	3.1 (0.95, 9.9)	2.5 (0.68, 8.9)
0.65 (0.28, 1.5)	2.7 (0.30, 23.0)	1.6 (0.52, 5.3)	0.51 (0.17, 1.5)	0.87 (0.29, 2.7)	0.70 (0.21, 2.4)
0.42 (0.19, 0.89)	1.7 (0.18, 17.0)	1.1 (0.41, 2.7)	0.33 (0.11, 0.98)	0.56 (0.19, 1.7)	0.45 (0.13, 1.5)
Control	4.1 (0.54, 31.0)	2.5 (1.2, 5.3)	0.78 (0.37, 1.6)	1.3 (0.65, 2.8)	1.1 (0.44, 2.6)
0.24 (0.032, 1.9)	IL2	0.61 (0.068, 5.8)	0.19 (0.023, 1.6)	0.32 (0.039, 2.7)	0.26 (0.030, 2.4)
0.40 (0.19, 0.82)	1.6 (0.17, 15.0)	Obinutuzumab	0.31 (0.10, 0.89)	0.53 (0.18, 1.5)	0.43 (0.13, 1.4)
1.3 (0.62, 2.7)	5.3 (0.62, 43.0)	3.2 (1.1, 9.6)	Ocrelizumab-1000mg	1.7 (0.83, 3.5)	1.4 (0.43, 4.4)
0.75 (0.36, 1.5)	3.1 (0.37, 25.0)	1.9 (0.65, 5.5)	0.58 (0.28, 1.2)	Ocrelizumab-400mg	0.81 (0.25, 2.5)
0.93 (0.38, 2.3)	3.8 (0.42, 33.0)	2.3 (0.72, 7.7)	0.72 (0.23, 2.3)	1.2 (0.40, 3.9)	Rituximab

a SUCRA, Surface Under the Cumulative Ranking Curve;

b CRR, Complete Response Rate;

c HR, Hazard Ratio;

d Cls, Confidence Intervals;

e Abatacept approximating 10 mg/kg on all infusion days; obinutuzumab was administered as a blinded intravenous infusion of 1,000 mg on day 1 and weeks 2, 24, and 26;

f Abatacept 30 mg/kg on days 1, 15, 29, and 57, followed by abatacept approximating 10 mg/kg (weight tiered: 500 mg for patients weighing 60 kg, 750 mg for patients 60–100 kg, 1,000 mg for patients 100 kg) on days 85, 113, 141, 169, 197, 225, 253, 281, 309, and 337;

g Anifrolumab basic regimen (BR; 300 mg, corresponding to SLE dosing10–12);

h Anifrolumab intensified regimen (IR; 900 mg for the first three doses, 300 mg thereafter);

i 1,000 mg ocrelizumab given as an intravenous (IV) infusion on days 1 and 15, followed by a single infusion at week 16 and every 16 weeks thereafter;

j 400 mg ocrelizumab given as an intravenous (IV) infusion on days 1 and 15, followed by a single infusion at week 16 and every 16 weeks thereafter.

### CRR “Time Window” analysis

**Figure 3:**
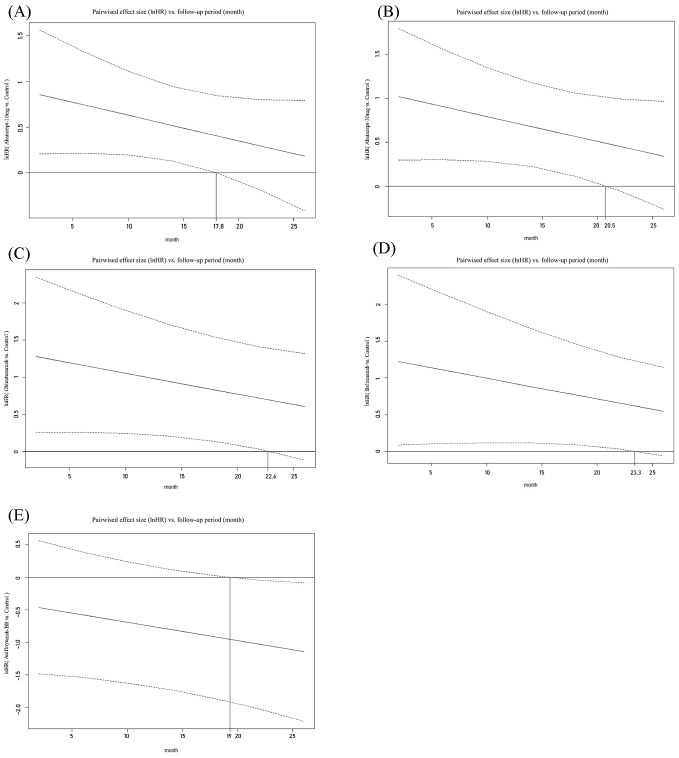
Curves depicting pairwise comparisons of effect sizes (lnHR) versus follow-up period for CRR. **(A)** Abatacept-10 mg versus Control; **(B)** Abatacept-30 mg versus Control; **(C)** Obinutuzumab versus Control; **(D)** Belimumab versus Control; **(E)** Anifrolumab-BR versus Control. (1) The upper and lower dashed lines represent the logarithm of the confidence interval values, while the solid line indicates the mean. The auxiliary lines denote the time points at which there are significant differences in effect sizes (InHR) between the two treatments in pairwise comparisons. (2) InHR, Napierian logarithm of the hazard ratio. (3) Abatacept-10mg, Abatacept at a dose approximating 10 mg/kg on all infusion days. (4) Abatacept-30mg, Abatacept at 30 mg/kg on days 1, 15, 29, and 57, followed by a dose approximating 10 mg/kg (weight-tiered: 500 mg for patients weighing ≤ 60 kg, 750 mg for patients 60-100 kg, 1,000 mg for patients > 100 kg) on days 85, 113, 141, 169, 197, 225, 253, 281, 309, and 337. (5) Anifrolumab-BR, Anifrolumab basic regimen (BR: 300 mg, corresponding to SLE dosing guidelines of 10-12 mg/kg).

Significant differences in treatment efficacy compared to the control group were observed for the four drugs studied within a specific time zone. Abatacept-10mg showed significant superiority from the initiation of treatment, which terminated at 17.8 months. However, this distinction was not statistically significant beyond that point. Similarly, abatacept-30mg showed significant superiority over the control group in ES at 20.5 months, belimumab at 23.3 months, and obinutuzumab at 22.6 months, with a decreasing trend. However, these variances were not statistically significant thereafter. Conversely, anifrolumab-BR did not show a significant difference in CRR compared to the control group within the initial 19 months after treatment commencement, but it exhibited inferiority to the control group after 19 months. Please refer to **Figure 3**.

### Secondary endpoint

In the raw analysis: compared to the control group, abatacept-30mg (HR=1.57, 95% CI: 1.12 to 2.21) and obinutuzumab (HR=2.18, 95% CI: 1.22 to 3.88) showed significance, while the rest did not demonstrate significance, as shown in **Supplementary Table 2**.

In the sensitivity analysis, compared to the control group, abatacept-30mg (HR=1.6, 95% CI: 1.1 to 2.2) and obinutuzumab (HR=2.4, 95% CI: 1.3 to 4.4) demonstrated a significant difference, resembling the raw analysis. In pairwise comparison, abatacept-30mg did not show a significant difference compared to obinutuzumab (HR=0.66, 95% CI: 0.33 to 1.3). Obinutuzumab demonstrated superiority over anifrolumab-BR (HR=0.23, 95% CI: 0.077 to 0.67), as seen in **Table 3**.

**Table 3:** Matrix and SUCRA^a^ of pairwise comparisons of regimens on PRR^b^ after Bayesian network meta-regression (shown as HR^c^ and 95% Cls^d^).

	Abatacept-10mg^e^	Abatacept-30mg^f^	Anifrolumab-BR^g^	Anifrolumab-IR^h^	Belimumab
SUCRA(%)	34	55	3	56	63
Abatacept-10mg	Abatacept-10mg	1.2 (0.89, 1.8)	0.43 (0.17, 1.1)	1.3 (0.54, 3.1)	1.4 (0.70, 2.7)
Abatacept-30mg	0.80 (0.57, 1.1)	Abatacept30mg	0.35 (0.14, 0.89)	1.0 (0.43, 2.5)	1.1 (0.56, 2.2)
Anifrolumab-BR	2.3 (0.90, 5.9)	2.9 (1.1, 7.4)	Anifrolumab-BR	3.0 (1.3, 7.0)	3.2 (1.1, 9.4)
Anifrolumab-IR	0.77 (0.32, 1.9)	0.97 (0.40, 2.3)	0.34 (0.14, 0.79)	Anifrolumab-IR	1.1 (0.38, 3.0)
Belimumab	0.71 (0.37, 1.4)	0.89 (0.46, 1.8)	0.31 (0.11, 0.93)	0.92 (0.33, 2.6)	Belimumab
Control	1.3 (0.90, 1.8)	1.6 (1.1, 2.2)	0.55 (0.23, 1.3)	1.6 (0.72, 3.7)	1.8 (0.96, 3.1)
IL2	0.47 (0.075, 3.0)	0.58 (0.093, 3.7)	0.20 (0.027, 1.5)	0.60 (0.084, 4.4)	0.65 (0.091, 4.8)
Obinutuzumab	0.52(0.26,1.1)	0.66(0.33,1.3)	0.23(0.077,0.67)	0.68(0.24,1.9)	0.74(0.34,1.6)
Ocrelizumab-1000mg	0.87 (0.40, 1.9)	1.1 (0.50, 2.4)	0.38 (0.12, 1.1)	1.1 (0.39, 3.2)	1.2 (0.46, 3.1)
Ocrelizumab-400mg	0.70 (0.32, 1.5)	0.88 (0.40, 1.9)	0.30 (0.099, 0.93)	0.91 (0.31, 2.6)	0.98 (0.37, 2.5)
Rituximab	0.78(0.29,2.1)	0.97(0.37,2.6)	0.34(0.096,1.2)	1.0(0.30,3.4)	1.1(0.36,3.3)
Control	IL2	Obinutuzumab	Ocrelizumab-1000mg^i^	Ocrelizumab-400mg^j^	Rituximab
26	73	82	47	64	55
0.80 (0.57, 1.1)	2.1 (0.33, 13.)	1.9 (0.95, 3.8)	1.2 (0.53, 2.5)	1.4 (0.65, 3.1)	1.3 (0.49, 3.4)
0.64 (0.45, 0.90)	1.7 (0.27, 11.)	1.5 (0.76, 3.1)	0.92 (0.43, 2.0)	1.1 (0.52, 2.5)	1.0 (0.39, 2.7)
1.8 (0.76, 4.4)	4.9 (0.65, 36.)	4.4 (1.5, 13.)	2.6 (0.88, 8.1)	3.3 (1.1, 10.)	2.9 (0.84, 10.)
0.62 (0.27, 1.4)	1.7 (0.23, 12.)	1.5 (0.52, 4.1)	0.89 (0.31, 2.6)	1.1 (0.38, 3.2)	0.99 (0.29, 3.4)
0.56 (0.32, 1.0)	1.5 (0.21, 11.)	1.4 (0.63, 3.0)	0.82 (0.32, 2.2)	1.0 (0.39, 2.7)	0.91 (0.31, 2.8)
Control	2.7 (0.43, 16.)	2.4 (1.3, 4.4)	1.4 (0.73, 2.9)	1.8 (0.88, 3.6)	1.6 (0.65, 4.0)
0.37 (0.062, 2.3)	IL2	0.89 (0.13, 6.5)	0.54 (0.079, 3.7)	0.67 (0.098, 4.5)	0.60 (0.081, 4.5)
0.42(0.23,0.77)	1.1(0.15,7.8)	Obinutuzumab	0.60(0.24,1.6)	0.75(0.29,1.9)	0.67(0.22,2.1)
0.69 (0.34, 1.4)	1.9 (0.27, 13.)	1.7 (0.64, 4.2)	Ocrelizumab-1000mg	1.2 (0.63, 2.4)	1.1 (0.36, 3.5)
0.56 (0.28, 1.1)	1.5 (0.22, 10.)	1.3 (0.52, 3.5)	0.81 (0.41, 1.6)	Ocrelizumab-400mg	0.90 (0.29, 2.8)
0.62(0.25,1.5)	1.7(0.22,12.)	1.5(0.49,4.5)	0.90(0.29,2.8)	1.1(0.35,3.5)	Rituximab

a SUCRA, Surface Under the Cumulative Ranking Curve;

b PRR, Partial Response Rate;

c HR, Hazard Ratio;

d Cls, Confidence Intervals;

e Abatacept approximating 10 mg/kg on all infusion days; obinutuzumab was administered as a blinded intravenous infusion of 1,000 mg on day 1 and weeks 2, 24, and 26;

f Abatacept 30 mg/kg on days 1, 15, 29, and 57, followed by abatacept approximating 10 mg/kg (weight tiered: 500 mg for patients weighing 60 kg, 750 mg for patients 60–100 kg, 1,000 mg for patients 100 kg) on days 85, 113, 141, 169, 197, 225, 253, 281, 309, and 337;

g Anifrolumab basic regimen (BR; 300 mg, corresponding to SLE dosing10–12);

h Anifrolumab intensified regimen (IR; 900 mg for the first three doses, 300 mg thereafter);

i 1,000 mg ocrelizumab given as an intravenous (IV) infusion on days 1 and 15, followed by a single infusion at week 16 and every 16 weeks thereafter;

j 400 mg ocrelizumab given as an intravenous (IV) infusion on days 1 and 15, followed by a single infusion at week 16 and every 16 weeks thereafter.

### PRR “Time Window” analysis

**Figure 4:**
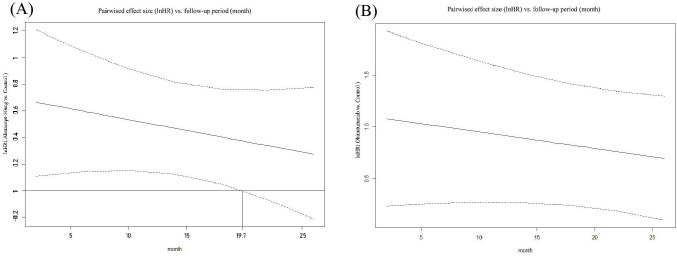
Curves depicting pairwise comparisons of effect sizes (lnHR) versus follow-up period for PRR. **(A)** Abatacept-30 mg versus Control; **(B)** Obinutuzumab versus Control. (1) The upper and lower dashed lines represent the logarithm of the Confidence Interval values, while the solid line indicates the mean. The auxiliary lines denote the time points at which there are significant differences in effect sizes (InHR) between the two medications in pairwise comparisons. (2) InHR, Napierian logarithm of the hazard ratio.

Abatacept-30mg, at 19.7 months after treatment initiation, showed significant superiority in ES as measured by lnHR compared to the control group, with a downward trend. However, obinutuzumab showed no “time window” in ES measured by lnHR, indicating that its superiority persisted from the beginning to the end (the longest follow-up period in our study), regardless of the follow-up period. Please refer to **Figure 4**.

### Tertiary endpoint

In the raw analysis: Comparison of matrix AE results revealed no significant differences between abatacept-10mg, abatacept-30mg, anifrolumab-BR, anifrolumab-IR, belimumab, obinutuzumab, ocrelizumab-1000mg, ocrelizumab-400mg, and rituximab when compared to the control group, as shown in **Supplementary Table 3**.

In the sensitivity analysis, comparison of matrix AE results demonstrated no significant differences between abatacept-10mg, abatacept-30mg, anifrolumab-BR, anifrolumab-IR, belimumab, obinutuzumab, ocrelizumab-1000mg, ocrelizumab-400mg, and rituximab when compared to the control group, similar to the raw analysis, as shown in **Table 4**.

Table 4Matrix and SUCRA^a^ of pairwise comparisons of regimens on AE^b^ after Bayesian network meta-regression (shown as HR^c^ and 95% Cls^d^).

**Table:** 

	Abatacept-10mg^e^	Abatacept-30mg^f^	Anifrolumab-IR^g^	Anifrolumab-IR^h^	Belimumab
SUCRA(%)	47	35	59	65	64
Abatacept-10mg	Abatacept-10mg	0.78 (0.25, 2.5)	1.4 (0.17, 11.0)	1.6 (0.22, 12.0)	1.6 (0.29, 8.1)
Abatacept-30mg	1.3 (0.40, 4.0)	Abatacept-30mg	1.7 (0.17, 18.0)	2.0 (0.21, 19.0)	2.0 (0.34, 11.0)
Anifrolumab-BR	0.74 (0.091, 5.9)	0.58 (0.056, 6.1)	Anifrolumab-IR	1.2 (0.24, 5.5)	1.1 (0.086, 15.0)
Anifrolumab-IR	0.64 (0.086, 4.6)	0.50 (0.053, 4.7)	0.86 (0.18, 4.1)	Anifrolumab-IR	0.99 (0.078, 12.0)
Belimumab	0.64 (0.12, 3.4)	0.50 (0.089, 2.9)	0.87 (0.066, 12.0)	1.0 (0.081, 13.0)	Belimumab
Control	1.0 (0.39, 2.8)	0.82 (0.22, 3.1)	1.4 (0.27, 7.7)	1.6 (0.35, 7.9)	1.6 (0.31, 8.2)
Obinutuzumab	0.69 (0.12, 4.4)	0.54 (0.084, 3.7)	0.95 (0.064, 14.0)	1.1 (0.080, 15.0)	1.1 (0.15, 7.7)
Ocrelizumab-1000mg	2.1 (0.36, 13.0)	1.7 (0.21, 14.0)	2.9 (0.47, 18.0)	3.3 (0.60, 19.0)	3.3 (0.31, 36.0)
Ocrelizumab-400mg	1.4 (0.23, 8.1)	1.1 (0.13, 8.6)	1.8 (0.30, 12.0)	2.1 (0.38, 12.0)	2.1 (0.20, 23.0)
Rituximab	0.37 (0.022, 6.1)	0.29 (0.014, 6.0)	0.50 (0.027, 9.3)	0.58 (0.033, 9.9)	0.57 (0.023, 14.0)

**Table:** 

Control	Obinutuzumab	Ocrelizumab-1000mg^i^	Ocrelizumab-400mg^j^	Rituximab
45	60	15	35	75
0.96 (0.36, 2.6)	1.4 (0.23, 8.6)	0.47 (0.078, 2.8)	0.74 (0.12, 4.4)	2.7 (0.16, 45.0)
1.2 (0.32, 4.6)	1.8 (0.27, 12.0)	0.60 (0.073, 4.8)	0.94 (0.12, 7.5)	3.5 (0.17, 71.0)
0.71 (0.13, 3.7)	1.1 (0.071, 16.0)	0.35 (0.055, 2.1)	0.54 (0.086, 3.4)	2.0 (0.11, 37.0)
0.61 (0.13, 2.9)	0.92 (0.065, 12.0)	0.30 (0.053, 1.7)	0.47 (0.084, 2.6)	1.7 (0.10, 30.0)
0.62 (0.12, 3.2)	0.93 (0.13, 6.7)	0.31 (0.028, 3.2)	0.48 (0.043, 5.1)	1.8 (0.069, 43.0)
Control	1.5 (0.25, 9.0)	0.49 (0.14, 1.7)	0.77 (0.22, 2.6)	2.8 (0.23, 36.0)
0.67 (0.11, 4.0)	Obinutuzumab	0.33 (0.027, 3.9)	0.52 (0.042, 6.2)	1.9 (0.070, 51.0)
2.0 (0.60, 6.9)	3.0 (0.26, 37.0)	Ocrelizumab-1000mg	1.6 (0.59, 4.1)	5.8 (0.41, 82.0)
1.3 (0.38, 4.5)	1.9 (0.16, 24.0)	0.64 (0.24, 1.7)	Ocrelizumab-400mg	3.7 (0.26, 52.0)
0.35 (0.028, 4.4)	0.53 (0.019, 14.0)	0.17 (0.012, 2.4)	0.27 (0.019, 3.9)	Rituximab

a SUCRA, Surface Under the Cumulative Ranking Curve;

b AE, Adverse Events;

c HR, Hazard Ratio;

d Cls, Confidence Intervals;

e Abatacept approximating 10 mg/kg on all infusion days; obinutuzumab was administered as a blinded intravenous infusion of 1,000 mg on day 1 and weeks 2, 24, and 26;

f Abatacept 30 mg/kg on days 1, 15, 29, and 57, followed by abatacept approximating 10 mg/kg (weight tiered: 500 mg for patients weighing 60 kg, 750 mg for patients 60–100 kg, 1,000 mg for patients 100 kg) on days 85, 113, 141, 169, 197, 225, 253, 281, 309, and 337;

g Anifrolumab basic regimen (BR; 300 mg, corresponding to SLE dosing10–12);

h Anifrolumab intensified regimen (IR; 900 mg for the first three doses, 300 mg thereafter);

i 1,000 mg ocrelizumab given as an intravenous (IV) infusion on days 1 and 15, followed by a single infusion at week 16 and every 16 weeks thereafter;

j 400 mg ocrelizumab given as an intravenous (IV) infusion on days 1 and 15, followed by a single infusion at week 16 and every 16 weeks thereafter.

In the raw analysis: Comparison of matrix IAE results revealed no significant differences between abatacept-10mg, abatacept-30mg, anifrolumab-BR, IL2, anifrolumab-IR, belimumab, obinutuzumab, ocrelizumab-1000mg, ocrelizumab-400mg, and rituximab when compared to the control group, as shown in **Supplementary Table 4**.

In the sensitivity analysis, comparison of matrix IAE results demonstrated no significant differences between abatacept-10mg, abatacept-30mg, anifrolumab-BR, anifrolumab-IR, belimumab, obinutuzumab, ocrelizumab-1000mg, ocrelizumab-400mg, and rituximab when compared to the control group, similar to the raw analysis, as shown in **Table 5**.

**Table 5:** Matrix and SUCRA^a^ of pairwise comparisons of regimens on IAE^b^ after Bayesian network meta-regression (shown as HR^c^ and 95% Cls^d^).

	Abatacept-10mg^e^	Abatacept-30mg^f^	Belimumab	Control
SUCRA(%)	40	62	35	35
Abatacept-10mg	Abatacept-10mg	1.4 (0.57, 3.3)	0.86 (0.22, 3.5)	0.96 (0.48, 1.9)
Abatacept-30mg	0.73 (0.30, 1.8)	Abatacept-30mg	0.63 (0.15, 2.6)	0.70 (0.28, 1.7)
Belimumab	1.2 (0.28, 4.6)	1.6 (0.38, 6.5)	Belimumab	1.1 (0.32, 3.8)
Control	1.0 (0.52, 2.1)	1.4 (0.59, 3.5)	0.90 (0.27, 3.1)	Control
IL2	3.6 (0.40, 32.0)	4.9 (0.47, 51.0)	3.1 (0.24, 42.0)	3.4 (0.43, 27.0)
Obinutuzumab	0.43 (0.10, 1.8)	0.59 (0.14, 2.5)	0.37 (0.076, 1.9)	0.41 (0.11, 1.5)
Ocrelizumab-1000mg	0.60 (0.20, 1.9)	0.82 (0.22, 3.1)	0.52 (0.10, 2.6)	0.57 (0.24, 1.4)
Ocrelizumab-400mg	0.60 (0.20, 1.8)	0.82 (0.22, 3.0)	0.52 (0.11, 2.6)	0.58 (0.24, 1.4)
Rituximab	1.1 (0.27, 4.3)	1.5 (0.32, 6.8)	0.92 (0.15, 5.6)	1.0 (0.31, 3.4)
IL2	Obinutuzumab	Ocrelizumab-1000mg^g^	Ocrelizumab-400mg^h^	Rituximab
10	84	73	73	39
0.28 (0.031, 2.5)	2.3 (0.56, 9.8)	1.7 (0.54, 5.1)	1.7 (0.54, 5.1)	0.93 (0.23, 3.8)
0.20 (0.020, 2.1)	1.7 (0.40, 7.3)	1.2 (0.33, 4.5)	1.2 (0.33, 4.5)	0.69 (0.15, 3.2)
0.32 (0.024, 4.2)	2.7 (0.54, 13.0)	1.9 (0.38, 9.5)	1.9 (0.38, 9.4)	1.1 (0.18, 6.5)
0.29 (0.037, 2.3)	2.4 (0.68, 8.7)	1.7 (0.73, 4.2)	1.7 (0.73, 4.1)	0.98 (0.30, 3.2)
IL2	8.4 (0.62, 1.2e+02)	6.0 (0.71, 50.0)	6.0 (0.70, 50.0)	3.4 (0.34, 34.0)
0.12 (0.0087, 1.6)	Obinutuzumab	0.72 (0.14, 3.7)	0.71 (0.14, 3.6)	0.40 (0.065, 2.4)
0.17 (0.020, 1.4)	1.4 (0.27, 7.2)	Ocrelizumab-1000mg	1.0 (0.43, 2.3)	0.56 (0.13, 2.3)
0.17 (0.020, 1.4)	1.4 (0.27, 7.2)	1.0 (0.44, 2.3)	Ocrelizumab-400mg	0.56 (0.13, 2.3)
0.30 (0.030, 2.9)	2.5 (0.41, 15.0)	1.8 (0.43, 7.4)	1.8 (0.43, 7.4)	Rituximab

a SUCRA, Surface Under the Cumulative Ranking Curve;

b IAE, Infection Adverse Events;

c HR, Hazard Ratio;

d Cls, Confidence Intervals;

e Abatacept approximating 10 mg/kg on all infusion days; obinutuzumab was administered as a blinded intravenous infusion of 1,000 mg on day 1 and weeks 2, 24, and 26;

f Abatacept 30 mg/kg on days 1, 15, 29, and 57, followed by abatacept approximating 10 mg/kg (weight tiered: 500 mg for patients weighing 60 kg, 750 mg for patients 60–100 kg, 1,000 mg for patients 100 kg) on days 85, 113, 141, 169, 197, 225, 253, 281, 309, and 337;

g 1,000 mg ocrelizumab given as an intravenous (IV) infusion on days 1 and 15, followed by a single infusion at week 16 and every 16 weeks thereafter;

h 400 mg ocrelizumab given as an intravenous (IV) infusion on days 1 and 15, followed by a single infusion at week 16 and every 16 weeks thereafter.

Heterogeneity and inconsistency analysis is shown in **Supplementary Figures 2-9**. The Brooks-Gelman-Rubin diagnostic demonstrated that the inferential iterations for each MCMC were stable and reproducible for all outcomes. We used the history feature to confirm the models convergence in all outcomes. Detailed results are presented in **Supplementary Figures 10-17**.

## Discussion

To the best of our knowledge, this is the first time that the confounding factors of follow-up time have been corrected, and the efficacy and safety of biologic agents in the treatment of LN have been evaluated through Bayesian network meta-regression analysis. Although previous studies, such as the one conducted by Lee YH et al., have also investigated the efficacy and safety of biologic agents for LN, they did not employ regression analysis or correct for the confounding factor of follow-up time (11). Our research introduces methodological innovations by including a more comprehensive literature review and employing regression analysis to adjust for the impact of follow-up time on the results. Additionally, our study highlights the “time window” effect in the treatment with biologic agents.

In terms of the CRR for the treatment of LN, our study suggests that obinutuzumab, belimumab, abatacept-30mg, and abatacept-10mg were all effective treatment methods at the initial stage of treatment. However, considering the impact of time, with longer follow-up periods, the benefits of obinutuzumab, belimumab, abatacept-30mg, and abatacept-10mg did not significantly differ from those observed in the placebo group. Anifrolumab-BR showed inferior efficacy compared to the control group as the follow-up period extended.

From the perspective of PRR, the optimal interventions were obinutuzumab and abatacept-30mg. In the “time window” analysis of the secondary endpoint, obinutuzumab showed no “time window” in ES measured by lnHR, indicating that its superiority persisted from the beginning to the end of the longest follow-up period in the study.

The “time window” demonstrates the period during the early stages of treatment when the combination of biological agents plus standard treatment holds an advantage over the use of standard treatment alone. This phenomenon has been observed in the treatment of Ulcerative Colitis (UC) with biological agents (22). This suggests that biological agents have a prominent advantage in terms of the speed of efficacy compared to traditional treatment plans. Biological agents can more rapidly target the inflammatory response to alleviate symptoms, which is particularly important for patients in need of quick relief. In the later stages of follow-up, there is no significant difference in complete remission between the combination of biological agents plus standard treatment and standard treatment alone, indicating that, in the long term, standard therapy remains the base in the treatment of LN.

The observation of a “time window” effect in the therapeutic outcomes of certain biologic agents, specifically obinutuzumab, is intriguing and may be attributed to the dynamic response of LN to different treatments over time. The mechanism behind this phenomenon is likely related to the pharmacodynamic and pharmacokinetic properties of obinutuzumab, which targets the CD20 antigen on B-lymphocytes. This monoclonal antibody mediates B-cell lysis through various mechanisms, including engagement of immune effector cells, direct activation of intracellular death signaling pathways, and activation of the complement cascade, resulting in significant CD19 B-cell depletion (23). The population pharmacokinetic analysis of obinutuzumab reveals a unique characteristic of its elimination, which is a combination of linear and time-dependent nonlinear clearance pathways (24). The time-dependent pathway diminishes as treatment progresses, indicating target-mediated drug disposition. This suggests that the concentration of obinutuzumab in the body may decrease over time due to the saturation of its target CD20 antigen on B cells. Consequently, the efficacy of obinutuzumab may decrease as the treatment continues, leading to the observed “time window” effect in the therapeutic outcomes.

Dysregulated B cell activation is a key driver in the pathogenesis of lupus, but monoclonal depleting antibodies, such as rituximab targeting the B cell antigen CD20, have failed to achieve primary efficacy endpoints in RCT (21, 25). Consequently, new “second-generation” anti-CD20 monoclonal antibodies have been designed to achieve greater B cell cytotoxicity. Obinutuzumab is one such drug. Compared to first-generation anti-CD20 antibodies, it has a stronger affinity for FcγRIII on effector cells (26), enabling it to exhibit stronger antibody-dependent cell cytotoxicity, enhanced direct B cell killing capabilities, and reduced reliance on complement-dependent cytotoxicity. Obinutuzumab outperforms rituximab in direct cell killing and antibody-dependent cellular cytotoxicity (ADCC), leading to more thorough B cell depletion in diseases such as follicular lymphoma, rheumatoid arthritis, and SLE. In SLE patient samples, obinutuzumab has demonstrated greater B cell cytotoxicity and activation of natural killer cells compared to rituximab, and it has been more effective than rituximab in treating mouse models of LN (27–29).

These findings are supported physiologically. In head-to-head studies, obinutuzumab has been shown to help improve clinical response, with greater improvements in anti-dsDNA antibodies, C3, C4, eGFR, and proteinuria observed with its use. Compared to placebo, obinutuzumab can rapidly and effectively deplete peripheral CD19 B cells without increasing the incidence of severe adverse events, severe infections, or death (12). These data suggest that B cell targeting may still have a place in the treatment of lupus nephritis, provided that anti-CD20 agents are selected for their ability to induce deep and durable B cell depletion.

In terms of safety, the six biological agents abatacept, anifrolumab, belimumab, obinutuzumab, ocrelizumab, and rituximab exhibited AE outcomes, with the addition of IL-2 among seven agents showing IAE results, and these findings revealed no significant differences in AEs or IAE between the biological agents and the control group.

### Limitation

1. Firstly, the standard treatment regimens used in the control group in each RCT were partially divergent. However, in the heterogeneity test and inconsistency test,we found that all the *P* values were greater than 0.01, indicating there was no significant different in direct and indirect comparisons. As such difference of the control group across enrolled studies did not compromise our results.
2. The number of RCT studies included is small, with only one to three studies per biological agent. Among them, the number of LN in five studies is less than 100, potentially compromising our results
3. The included studies were limited in number and the longest follow-up time was not long enough, given that the disease course of SLE spans a lifespan. Additionally, there was a considerable dispersion in the follow-up time points of the studies, which affected the precision of the plotted curves.

### Future research

For more precise comparisons with regard to agents safety, Food and Drug Administration Adverse Event Reporting System (FAERS) could be exploited owing to its tremendous real-world data.

## Conclusion

Considering the effectiveness, safety, and “time window” of biological agents, we recommend obinutuzumab as the preferred treatment for LN. In the future, larger samples and longer follow-up studies are needed to confirm our findings.

## Data Availability

The original contributions presented in the study are included in the article/**Supplementary Material**. Further inquiries can be directed to the corresponding author.
