# Supplementary material for: Biologicals for the treatment of lupus nephritis: a Bayesian network meta-regression analysis
Source: Front Immunol. 2024 Aug 30;15:1445814. doi: 10.3389/fimmu.2024.1445814 (PMC11392858; doi:10.3389/fimmu.2024.1445814)
Supplement: Supplementary file 1 [file DataSheet1.pdf]

Biologicals for the Treatment of Lupus Nephritis: A Bayesian network meta-regression analysis

1 Supplementary Data

Supplementary Data 1:Search strategy in five electronic databases.

Search strategy of Pubmed	
#1	("Lupus Erythematosus, Systemic") OR("Systemic Lupus Erythematosus") OR("Libman-Sacks Disease") OR("Disease, Libman-Sacks") OR("Libman Sacks Disease")
#2	("Lupus Nephritis") OR ("Glomerulonephritides, Lupus") OR ("Lupus Glomerulonephritides") OR ("Lupus Glomerulonephritis") OR ("Nephritis, Lupus") OR ("Lupus Nephritides") OR ("Nephritides, Lupus")
#3	("Orencia") OR ("BMS 188667") OR ("BMS-188667") OR ("BMS188667") OR ("Belatacept") OR ("BMS224818") OR ("BMS-224818") OR ("BMS 224818") OR ("CTLA-4-Ig") OR ("CTLA4-Ig Immunoconjugate") OR ("CTLA4 Ig Immunoconjugate") OR ("Immunoconjugate, CTLA4-Ig") OR ("CTLA4-Fc") OR ("CTLA4-Ig") OR ("Cytotoxic T Lymphocyte-Associated Antigen 4-Immunoglobulin") OR ("Cytotoxic T Lymphocyte Associated Antigen 4 Immunoglobulin") OR ("LEA29Y") OR ("Nulojix")
#4	("anifrolumab") OR ("MEDI-546") OR ("Saphnelo")
#5	("belimumab") OR ("LymphoStat-B") OR ("Benlysta") OR ("GSK-1550188") OR ("GSK1550188") OR ("BEL-114333") OR ("BEL114333") OR ("HGS-1006") OR ("HGS1006")
#6	("Interleukin-2") OR ("Interleukin 2") OR ("IL2") OR ("Interleukin II") OR ("Interleukine 2") OR ("Lymphocyte Mitogenic Factor") OR ("Mitogenic Factor, Lymphocyte") OR ("T-Cell Growth Factor") OR ("T Cell Growth Factor") OR ("T-Cell Stimulating Factor") OR ("T Cell Stimulating Factor") OR ("TCGF") OR ("IL-2") OR ("Thymocyte Stimulating Factor") OR ("RU 49637") OR ("RU-49637") OR ("RU49637") OR ("Ro-23-6019") OR ("Ro236019") OR ("Ro 23 6019") OR ("Ro-236019") OR ("Ro 236019")
#7	("Obinutuzumab")
#8	("ocrelizumab") OR ("PR 070769") OR ("PR-070769") OR ("PR070769") OR ("R 1594") OR ("R-1594") OR ("R1594") OR ("RG-1594") OR ("Ocrevus")

#9	("Rituximab") OR ("CD20 Antibody, Rituximab") OR ("Rituximab CD20 Antibody") OR ("Rituxan") OR ("IDEC-C2B8 Antibody") OR ("IDEC C2B8 Antibody") OR ("IDEC-C2B8") OR ("IDEC C2B8") OR ("Mabthera") OR ("GP2013")
#10	("Biological Therapy") OR ("Biological Therapies") OR ("Therapies, Biological") OR ("Therapy, Biological") OR ("Biologic Therapy") OR ("Biologic Therapies") OR ("Therapies, Biologic") OR ("Therapy, Biologic") OR ("Biotherapy") OR ("Biotherapies")
#11	("epratuzumab")
#12	("telitacicept")
#13	("Azathioprine") OR ("Azothioprine") OR ("Imuran") OR ("Immuran") OR ("Imurel") OR ("Azathioprine Sulfate") OR ("Azathioprine Sodium") OR ("Sodium, Azathioprine") OR ("Azathioprine Sodium Salt")
#14	("Cyclophosphamide") OR ("Cytoxan") OR ("Endoxan") OR ("Neosar") OR ("Procytox") OR ("Sendoxan") OR ("Cyclophosphamide, (S)-Isomer") OR ("Cyclophosphamide, (R)-Isomer") OR ("NSC-26271") OR ("NSC26271") OR ("NSC 26271") OR ("B-518") OR ("B518") OR ("B 518") OR ("Cytophosphan") OR ("Cyclophosphamide Anhydrous") OR ("Cyclophosphane") OR ("Cytophosphane") OR ("Cyclophosphamide Monohydrate") OR ("(+,-)-2-(bis(2-Chloroethyl)amino)tetrahydro-2H-1,3,2-oxazaphosphorine 2-Oxide Monohydrate")
#15	("Mycophenolic Acid") OR ("Cellcept") OR ("Mycophenolate Mofetil") OR ("Mofetil, Mycophenolate") OR ("Mycophenolic Acid Morpholinoethyl Ester") OR ("RS 61443") OR ("RS-61443") OR ("RS61443") OR ("Mycophenolate Mofetil Hydrochloride") OR ("Mofetil Hydrochloride, Mycophenolate") OR ("Myfortic") OR ("Mycophenolate Sodium") OR ("Sodium Mycophenolate") OR ("Mycophenolate, Sodium")
#16	("Tacrolimus") OR ("Prograf") OR ("Prograft") OR ("Anhydrous Tacrolimus") OR ("Tacrolimus, Anhydrous") OR ("Tacrolimus Anhydrous") OR ("Anhydrous, Tacrolimus") OR ("FK-506") OR ("FK 506") OR ("FK506") OR ("FR-900506") OR ("FR900506") OR ("FR 900506")
#17	("voclosporin") OR ("ISATX247") OR ("ISA(TX)247") OR ("ISA 247") OR ("ISA-247") OR ("lupkynis")
#18	("Glucocorticoids") OR ("Glucocorticoid") OR ("Glucocorticoid Effect") OR ("Effect, Glucocorticoid") OR ("Glucorticoid Effects") OR ("Effects, Glucorticoid")
#19	"Randomized Controlled Trials as Topic" OR "Clinical Trial")

#20	#2 OR #3 OR #4 OR #5 OR #6 OR #7 OR #8 OR #9 OR #10 OR #11 OR #12 OR #13 OR #14 OR #15 OR #16 OR #17 OR #18 OR #19
#21	#1 AND #19 AND #20

Search strategy of Embase	
#1	(“Lupus Erythematosus, Systemic”/exp OR “Lupus Erythematosus Disseminatus”) OR (“Systemic Lupus Erythematosus”/exp OR “Systemic Lupus Erythematosus”) OR (“Libman-Sacks Disease”/exp OR “Libman-Sacks Disease”) OR (“Disease, Libman-Sacks”/exp OR “Disease, Libman-Sacks”) OR (“Libman Sacks Disease” /exp OR “Libman Sacks Disease”)
#2	("Lupus Nephritis"/exp OR "glomerulonephritides lupus"/exp OR "lupus glomerulonephritides"/exp OR "lupus glomerulonephritis"/exp OR "nephritis lupus"/exp OR "lupus nephritides"/exp OR "nephritides lupus"/exp)
#3	("orencia"/exp OR "bms 188667"/exp OR "bms-188667"/exp OR "bms188667"/exp OR "belatacept"/exp OR "bms224818"/exp OR "bms-224818"/exp OR "bms 224818"/exp OR "ctla-4-ig"/exp OR "ctla4-ig immunoconjugate"/exp OR "ctla4 ig immunoconjugate"/exp OR "immunoconjugate, ctle4-ig"/exp OR "ctla4-fc"/exp OR "ctla4-ig"/exp OR "cytotoxic t lymphocyte-associated antigen 4-immunoglobulin"/exp OR "cytotoxic t lymphocyte associated antigen 4 immunoglobulin"/exp OR "lea29y"/exp OR "nulojix"/exp)
#4	("anifrolumab"/exp OR "medi-546"/exp OR "saphnelo"/exp)
#5	("belimumab"/exp OR "lymphostat-b"/exp OR "benlysta"/exp OR "gsk-1550188"/exp OR "gsk1550188"/exp OR "bel-114333"/exp OR "bel114333"/exp OR "hgs-1006"/exp OR "hgs1006"/exp)
#6	("interleukin-2"/exp OR "interleukin 2"/exp OR "il2"/exp OR "interleukin ii"/exp OR "interleukine 2"/exp OR "lymphocyte mitogenic factor"/exp OR "mitogenic factor, lymphocyte"/exp OR "t-cell growth factor"/exp OR "t cell growth factor"/exp OR "t-cell stimulating factor"/exp OR "t cell stimulating factor"/exp OR "tcgf"/exp OR "il-2"/exp OR "thymocyte stimulating factor"/exp OR "ru 49637"/exp OR "ru-49637"/exp OR "ru49637"/exp OR "ro-23-6019"/exp OR "ro236019"/exp OR "ro 23 6019"/exp OR "ro-236019"/exp OR "ro 236019"/exp)
#7	("obinutuzumab"/exp)
#8	("ocrelizumab"/exp OR "pr 070769"/exp OR "pr-070769"/exp OR "pr070769"/exp OR "r

	1594"/exp OR "r-1594"/exp OR "r1594"/exp OR "rg-1594"/exp OR "ocrevus"/exp)
#9	("rituximab"/exp OR "cd20 antibody, rituximab"/exp OR "rituximab cd20 antibody"/exp OR "rituxan"/exp OR "idec-c2b8 antibody"/exp OR "idec c2b8 antibody"/exp OR "idec-c2b8"/exp OR "idec c2b8"/exp OR "mabthera"/exp OR "gp2013"/exp)
#10	("biological therapy"/exp OR "biological therapies"/exp OR "therapies, biological"/exp OR "therapy, biological"/exp OR "biologic therapy"/exp OR "biologic therapies"/exp OR "therapies, biologic"/exp OR "therapy, biologic"/exp OR "biotherapy"/exp OR "biotherapies"/exp)
#11	("epratuzumab"/exp)
#12	("telitacicept"/exp)
#13	("azathioprine"/exp OR "azothioprine"/exp OR "imuran"/exp OR "immuran"/exp OR "imurel"/exp OR "azathioprine sulfate"/exp OR "azathioprine sodium"/exp OR "sodium, azathioprine"/exp OR "azathioprine sodium salt"/exp)
#14	("cyclophosphamide"/exp OR "cytoxan"/exp OR "endoxan"/exp OR "neosar"/exp OR "procytox"/exp OR "sendoxan"/exp OR "cyclophosphamide, (s)-isomer"/exp OR "cyclophosphamide, (r)-isomer"/exp OR "nsc-26271"/exp OR "nsc26271"/exp OR "nsc 26271"/exp OR "b-518"/exp OR "b518"/exp OR "b 518"/exp OR "cytophosphan"/exp OR "cyclophosphamide anhydrous"/exp OR "cyclophosphane"/exp OR "cytophosphane"/exp OR "cyclophosphamide monohydrate"/exp OR "(+,-)-2-(bis(2-chloroethyl)amino)tetrahydro-2H-1,3,2-oxazaphosphorine 2-Oxide Monohydrate"/exp)
#15	("mycophenolic acid"/exp OR "cellcept"/exp OR "mycophenolate mofetil"/exp OR "mofetil, mycophenolate"/exp OR "mycophenolic acid morpholinoethyl ester"/exp OR "rs 61443"/exp OR "rs-61443"/exp OR "rs61443"/exp OR "mycophenolate mofetil hydrochloride"/exp OR "mofetil hydrochloride, mycophenolate"/exp OR "myfortic"/exp OR "mycophenolate sodium"/exp OR "sodium mycophenolate"/exp OR "mycophenolate, sodium"/exp)
#16	("tacrolimus"/exp OR "prograf"/exp OR "prograft"/exp OR "anhydrous tacrolimus"/exp OR "tacrolimus, anhydrous"/exp OR "tacrolimus anhydrous"/exp OR "anhydrous, tacrolimus"/exp OR "fk-506"/exp OR "fk 506"/exp OR "fk506"/exp OR "fr-900506"/exp OR "fr900506"/exp OR "fr 900506"/exp) #17 ("voclosporin"/exp OR "isatx247"/exp OR "isa(tx)247"/exp OR "isa 247"/exp OR "isa-247"/exp OR "lupkynis"/exp)
#17	("voclosporin"/exp OR "isatx247"/exp OR "isa(tx)247"/exp OR "isa 247"/exp OR "isa-247"/exp OR "lupkynis"/exp)

#18	("glucocorticoids"/exp OR "glucocorticoid"/exp OR "glucocorticoid effect"/exp OR "effect, glucocorticoid"/exp OR "glucorticoid effects"/exp OR "effects, glucorticoid"/exp)
#19	("randomized controlled trials as topic"/exp OR "clinical trial"/exp)
#20	(#2 OR #3 OR #4 OR #5 OR #6 OR #7 OR #8 OR #9 OR #10 OR #11 OR #12 OR #13 OR #14 OR #15 OR #16 OR #17 OR #18 OR #19) #21 (#1 AND #19 AND #20)
#21	#1 AND #19 AND #20

Search strategy of MedlinePlus

#1	Lupus Erythematosus, Systemic or Lupus Nephritis

Search strategy of Cochrane Library

#1	("Lupus Erythematosus, Systemic" OR "Systemic Lupus Erythematosus" OR "Libman-Sacks Disease" OR "Disease, Libman-Sacks" OR "Libman Sacks Disease")
#2	("Lupus Nephritis" OR "Glomerulonephritides, Lupus" OR ("Lupus Glomerulonephritides") OR ("Lupus Glomerulonephritis") OR ("Nephritis, Lupus") OR ("Lupus Nephritides") OR ("Nephritides, Lupus")
#3	("Orencia") OR ("BMS 188667") OR ("BMS-188667") OR ("BMS188667") OR ("Belatacept") OR ("BMS224818") OR ("BMS-224818") OR ("BMS 224818") OR ("CTLA-4-Ig") OR ("CTLA4-Ig Immunoconjugate") OR ("CTLA4 Ig Immunoconjugate") OR ("Immunoconjugate, CTLA4-Ig") OR ("CTLA4-Fc") OR ("CTLA4-Ig") OR ("Cytotoxic T Lymphocyte-Associated Antigen 4-Immunoglobulin") OR ("Cytotoxic T Lymphocyte Associated Antigen 4 Immunoglobulin") OR ("LEA29Y") OR ("Nulojix")
#4	("anifrolumab") OR ("MEDI-546") OR ("Saphnelo")
#5	("belimumab") OR ("LymphoStat-B") OR ("Benlysta") OR ("GSK-1550188") OR ("GSK1550188") OR ("BEL-114333") OR ("BEL114333") OR ("HGS-1006") OR ("HGS1006")
#6	("Interleukin-2") OR ("Interleukin 2") OR ("IL2") OR ("Interleukin II") OR ("Interleukine 2") OR ("Lymphocyte Mitogenic Factor") OR ("Mitogenic Factor, Lymphocyte") OR ("T-Cell Growth Factor") OR ("T Cell Growth Factor") OR ("T-Cell Stimulating Factor") OR

	("T Cell Stimulating Factor") OR ("TCGF") OR ("IL-2") OR ("Thymocyte Stimulating Factor") OR ("RU 49637") OR ("RU-49637") OR ("RU49637") OR ("Ro-23-6019") OR ("Ro236019") OR ("Ro 23 6019") OR ("Ro-236019") OR ("Ro 236019")
#7	("Obinutuzumab")
#8	("ocrelizumab") OR ("PR 070769") OR ("PR-070769") OR ("PR070769") OR ("R 1594") OR ("R-1594") OR ("R1594") OR ("RG-1594") OR ("Ocrevus")
#9	("Rituximab") OR ("CD20 Antibody, Rituximab") OR ("Rituximab CD20 Antibody") OR ("Rituxan") OR ("IDEC-C2B8 Antibody") OR ("IDEC C2B8 Antibody") OR ("IDEC-C2B8") OR ("IDEC C2B8") OR ("Mabthera") OR ("GP2013")
#10	("Biological Therapy") OR ("Biological Therapies") OR ("Therapies, Biological") OR ("Therapy, Biological") OR ("Biologic Therapy") OR ("Biologic Therapies") OR ("Therapies, Biologic") OR ("Therapy, Biologic") OR ("Biotherapy") OR ("Biotherapies")
#11	("epratuzumab")
#12	("telitacicept")
#13	("Azathioprine") OR ("Azothioprine") OR ("Imuran") OR ("Immuran") OR ("Imurel") OR ("Azathioprine Sulfate") OR ("Azathioprine Sodium") OR ("Sodium, Azathioprine") OR ("Azathioprine Sodium Salt")
#14	("Cyclophosphamide") OR ("Cytoxan") OR ("Endoxan") OR ("Neosar") OR ("Procytox") OR ("Sendoxan") OR ("Cyclophosphamide, (S)-Isomer") OR ("Cyclophosphamide, (R)-Isomer") OR ("NSC-26271") OR ("NSC26271") OR ("NSC 26271") OR ("B-518") OR ("B518") OR ("B 518") OR ("Cytophosphan") OR ("Cyclophosphamide Anhydrous") OR ("Cyclophosphane") OR ("Cytophosphane") OR ("Cyclophosphamide Monohydrate") OR ("(+,-)-2-(bis(2-Chloroethyl)amino)tetrahydro-2H-1,3,2-oxazaphosphorine 2-Oxide Monohydrate")
#15	("Mycophenolic Acid") OR ("Cellcept") OR ("Mycophenolate Mofetil") OR ("Mofetil, Mycophenolate") OR ("Mycophenolic Acid Morpholinoethyl Ester") OR ("RS 61443") OR ("RS-61443") OR ("RS61443") OR ("Mycophenolate Mofetil Hydrochloride") OR ("Mofetil Hydrochloride, Mycophenolate") OR ("Myfortic") OR ("Mycophenolate Sodium") OR ("Sodium Mycophenolate") OR ("Mycophenolate, Sodium")
#16	("Tacrolimus") OR ("Prograf") OR ("Prograft") OR ("Anhydrous Tacrolimus") OR ("Tacrolimus, Anhydrous") OR ("Tacrolimus Anhydrous") OR ("Anhydrous, Tacrolimus") OR ("FK-506") OR ("FK 506") OR ("FK506") OR ("FR-900506") OR ("FR900506") OR ("FR 900506")

#17	("voclosporin") OR ("ISATX247") OR ("ISA(TX)247") OR ("ISA 247") OR ("ISA-247") OR ("lupkynis")
#18	("Glucocorticoids") OR ("Glucocorticoid") OR ("Glucocorticoid Effect") OR ("Effect, Glucocorticoid") OR ("Glucorticoid Effects") OR ("Effects, Glucorticoid")
#19	"Randomized Controlled Trials as Topic" OR "Clinical Trial")
#20	#2 OR #3 OR #4 OR #5 OR #6 OR #7 OR #8 OR #9 OR #10 OR #11 OR #12 OR #13 OR #14 OR #15 OR #16 OR #17 OR #18 OR #19
#21	#1 AND #19 AND #20

Search strategy of Cochrane Library	
#1	("Lupus Erythematosus, Systemic" OR "Systemic Lupus Erythematosus" OR "Libman-Sacks Disease" OR "Disease, Libman-Sacks" OR "Libman Sacks Disease")
#2	("Lupus Nephritis" OR "Glomerulonephritides, Lupus" OR "Lupus Glomerulonephritides" OR "Lupus Glomerulonephritis" OR "Nephritis, Lupus" OR "Lupus Nephritides" OR "Nephritides, Lupus")
#3	("Orencia" OR "BMS 188667" OR "BMS-188667" OR "BMS188667" OR "Belatacept" OR "BMS224818" OR "BMS-224818" OR "BMS 224818" OR "CTLA-4-Ig" OR "CTLA4-Ig Immunoconjugate" OR "CTLA4 Ig Immunoconjugate" OR "Immunoconjugate, CTLA4-Ig" OR "CTLA4-Fc" OR "CTLA4-Ig" OR "Cytotoxic T Lymphocyte-Associated Antigen 4-Immunoglobulin" OR "Cytotoxic T Lymphocyte Associated Antigen 4 Immunoglobulin" OR "LEA29Y" OR "Nulojix")
#4	("anifrolumab" OR "MEDI-546" OR "Saphnelo")
#5	("belimumab" OR "LymphoStat-B" OR "Benlysta" OR "GSK-1550188" OR "GSK1550188" OR "BEL-114333" OR "BEL114333" OR "HGS-1006" OR "HGS1006")
#6	("Interleukin-2" OR "Interleukin 2" OR "IL2" OR "Interleukin II" OR "Interleukine 2" OR "Lymphocyte Mitogenic Factor" OR "Mitogenic Factor, Lymphocyte" OR "T-Cell Growth Factor" OR "T Cell Growth Factor" OR "T-Cell Stimulating Factor" OR "T Cell Stimulating Factor" OR "TCGF" OR "IL-2" OR "Thymocyte Stimulating Factor" OR "RU 49637" OR "RU-49637" OR "RU49637" OR "Ro-23-6019" OR "Ro236019" OR "Ro 23 6019" OR "Ro-236019" OR "Ro 236019")

#7	("Obinutuzumab")
#8	("ocrelizumab" OR "PR 070769" OR "PR-070769" OR "PR070769" OR "R 1594" OR "R-1594" OR "R1594" OR "RG-1594" OR "Ocrevus")
#9	("Rituximab" OR "CD20 Antibody, Rituximab" OR "Rituximab CD20 Antibody" OR "Rituxan" OR "IDEC-C2B8 Antibody" OR "IDEC C2B8 Antibody" OR "IDEC-C2B8" OR "IDEC C2B8" OR "Mabthera" OR "GP2013")
#10	("Biological Therapy" OR "Biological Therapies" OR "Therapies, Biological" OR "Therapy, Biological" OR "Biologic Therapy" OR "Biologic Therapies" OR "Therapies, Biologic" OR "Therapy, Biologic" OR "Biotherapy" OR "Biotherapies")
#11	("epratuzumab")
#12	("telitacicept")
#13	("Azathioprine" OR "Azothioprine" OR "Imuran" OR "Immuran" OR "Imurel" OR "Azathioprine Sulfate" OR "Azathioprine Sodium" OR "Sodium, Azathioprine" OR "Azathioprine Sodium Salt")
#14	("Cyclophosphamide" OR "Cytoxan" OR "Endoxan" OR "Neosar" OR "Procytox" OR "Sendoxan" OR "Cyclophosphamide, S -Isomer" OR "Cyclophosphamide, R -Isomer" OR "NSC-26271" OR "NSC26271" OR "NSC 26271" OR "B-518" OR "B518" OR "B 518" OR "Cytophosphan" OR "Cyclophosphamide Anhydrous" OR "Cyclophosphane" OR "Cytophosphane" OR "Cyclophosphamide Monohydrate" OR " +,- -2- bis 2-Chloroethyl amino tetrahydro-2H-1,3,2-oxazaphosphorine 2-Oxide Monohydrate")
#15	("Mycophenolic Acid" OR "Cellcept" OR "Mycophenolate Mofetil" OR "Mofetil, Mycophenolate" OR "Mycophenolic Acid Morpholinoethyl Ester" OR "RS 61443" OR "RS-61443" OR "RS61443" OR "Mycophenolate Mofetil Hydrochloride" OR "Mofetil Hydrochloride, Mycophenolate" OR "Myfortic" OR "Mycophenolate Sodium" OR "Sodium Mycophenolate" OR "Mycophenolate, Sodium")
#16	("Tacrolimus" OR "Prograf" OR "Prograft" OR "Anhydrous Tacrolimus" OR "Tacrolimus, Anhydrous" OR "Tacrolimus Anhydrous" OR "Anhydrous, Tacrolimus" OR "FK-506" OR "FK 506" OR "FK506" OR "FR-900506" OR "FR900506" OR "FR 900506")
#17	("voclosporin" OR "ISATX247" OR "ISA TX 247" OR "ISA 247" OR "ISA-247" OR "lupkynis")

#18	("Glucocorticoids" OR "Glucocorticoid" OR "Glucocorticoid Effect" OR "Effect, Glucocorticoid" OR "Glucorticoid Effects" OR "Effects, Glucorticoid")
#19	("Randomized Controlled Trials as Topic" OR "Clinical Trial")
#20	#2 OR #3 OR #4 OR #5 OR #6 OR #7 OR #8 OR #9 OR #10 OR #11 OR #12 OR #13 OR #14 OR #15 OR #16 OR #17 OR #18 OR #19
#21	#1 AND #19 AND #20

Search strategy of Google Scholar	
#1	("Lupus Erythematosus, Systemic" OR "Systemic Lupus Erythematosus" OR "Libman-Sacks Disease" OR "Disease, Libman-Sacks" OR "Libman Sacks Disease")
#2	("Lupus Nephritis" OR "Glomerulonephritides, Lupus" OR "Lupus Glomerulonephritides" OR "Lupus Glomerulonephritis" OR "Nephritis, Lupus" OR "Lupus Nephritides" OR "Nephritides, Lupus")
#3	("Orencia" OR "BMS 188667" OR "BMS-188667" OR "BMS188667" OR "Belatacept" OR "BMS224818" OR "BMS-224818" OR "BMS 224818" OR "CTLA-4-Ig" OR "CTLA4-Ig Immunoconjugate" OR "CTLA4 Ig Immunoconjugate" OR "Immunoconjugate, CTLA4-Ig" OR "CTLA4-Fc" OR "CTLA4-Ig" OR "Cytotoxic T Lymphocyte-Associated Antigen 4-Immunoglobulin" OR "Cytotoxic T Lymphocyte Associated Antigen 4 Immunoglobulin" OR "LEA29Y" OR "Nulojix")
#4	("anifrolumab" OR "MEDI-546" OR "Saphnelo")
#5	("belimumab" OR "LymphoStat-B" OR "Benlysta" OR "GSK-1550188" OR "GSK1550188" OR "BEL-114333" OR "BEL114333" OR "HGS-1006" OR "HGS1006")
#6	("Interleukin-2" OR "Interleukin 2" OR "IL2" OR "Interleukin II" OR "Interleukine 2" OR "Lymphocyte Mitogenic Factor" OR "Mitogenic Factor, Lymphocyte" OR "T-Cell Growth Factor" OR "T Cell Growth Factor" OR "T-Cell Stimulating Factor" OR "T Cell Stimulating Factor" OR "TCGF" OR "IL-2" OR "Thymocyte Stimulating Factor" OR "RU 49637" OR "RU-49637" OR "RU49637" OR "Ro-23-6019" OR "Ro236019" OR "Ro 23 6019" OR "Ro-236019" OR "Ro 236019")
#7	("Obinutuzumab")

#8	("ocrelizumab" OR "PR 070769" OR "PR-070769" OR "PR070769" OR "R 1594" OR "R-1594" OR "R1594" OR "RG-1594" OR "Ocrevus")
#9	("Rituximab" OR "CD20 Antibody, Rituximab" OR "Rituximab CD20 Antibody" OR "Rituxan" OR "IDEC-C2B8 Antibody" OR "IDEC C2B8 Antibody" OR "IDEC-C2B8" OR "IDEC C2B8" OR "Mabthera" OR "GP2013")
#10	("Biological Therapy" OR "Biological Therapies" OR "Therapies, Biological" OR "Therapy, Biological" OR "Biologic Therapy" OR "Biologic Therapies" OR "Therapies, Biologic" OR "Therapy, Biologic" OR "Biotherapy" OR "Biotherapies")
#11	("epratuzumab")
#12	("telitacicept")
#13	("Azathioprine" OR "Azothioprine" OR "Imuran" OR "Immuran" OR "Imurel" OR "Azathioprine Sulfate" OR "Azathioprine Sodium" OR "Sodium, Azathioprine" OR "Azathioprine Sodium Salt")
#14	("Cyclophosphamide" OR "Cytoxan" OR "Endoxan" OR "Neosar" OR "Procytox" OR "Sendoxan" OR "Cyclophosphamide, S -Isomer" OR "Cyclophosphamide, R -Isomer" OR "NSC-26271" OR "NSC26271" OR "NSC 26271" OR "B-518" OR "B518" OR "B 518" OR "Cytophosphan" OR "Cyclophosphamide Anhydrous" OR "Cyclophosphane" OR "Cytophosphane" OR "Cyclophosphamide Monohydrate" OR " +,- -2- bis 2-Chloroethyl amino tetrahydro-2H-1,3,2-oxazaphosphorine 2-Oxide Monohydrate")
#15	("Mycophenolic Acid" OR "Cellcept" OR "Mycophenolate Mofetil" OR "Mofetil, Mycophenolate" OR "Mycophenolic Acid Morpholinoethyl Ester" OR "RS 61443" OR "RS-61443" OR "RS61443" OR "Mycophenolate Mofetil Hydrochloride" OR "Mofetil Hydrochloride, Mycophenolate" OR "Myfortic" OR "Mycophenolate Sodium" OR "Sodium Mycophenolate" OR "Mycophenolate, Sodium")
#16	("Tacrolimus" OR "Prograf" OR "Prograft" OR "Anhydrous Tacrolimus" OR "Tacrolimus, Anhydrous" OR "Tacrolimus Anhydrous" OR "Anhydrous, Tacrolimus" OR "FK-506" OR "FK 506" OR "FK506" OR "FR-900506" OR "FR900506" OR "FR 900506")
#17	("voclosporin" OR "ISATX247" OR "ISA TX 247" OR "ISA 247" OR "ISA-247" OR "lupkynis")
#18	("Glucocorticoids" OR "Glucocorticoid" OR "Glucocorticoid Effect" OR "Effect,

	Glucocorticoid" OR "Glucorticoid Effects" OR "Effects, Glucorticoid")
#19	("Randomized Controlled Trials as Topic" OR "Clinical Trial")
#20	#2 OR #3 OR #4 OR #5 OR #6 OR #7 OR #8 OR #9 OR #10 OR #11 OR #12 OR #13 OR #14 OR #15 OR #16 OR #17 OR #18 OR #19
#21	#1 AND #19 AND #20

Search strategy of Scopus	
#1	TITLE-ABS-KEY("Lupus Erythematosus, Systemic") OR TITLE-ABS-KEY("Systemic Lupus Erythematosus") OR TITLE-ABS-KEY("Libman-Sacks Disease") OR TITLE-ABS-KEY("Disease, Libman-Sacks") OR TITLE-ABS-KEY("Libman Sacks Disease")
#2	TITLE-ABS-KEY("Lupus Nephritis") OR TITLE-ABS-KEY("Glomerulonephritides, Lupus") OR TITLE-ABS-KEY("Lupus Glomerulonephritides") OR TITLE-ABS-KEY("Lupus Glomerulonephritis") OR TITLE-ABS-KEY("Nephritis, Lupus") OR TITLE-ABS-KEY("Lupus Nephritides") OR TITLE-ABS-KEY("Nephritides, Lupus")
#3	TITLE-ABS-KEY("Orencia") OR TITLE-ABS-KEY("BMS 188667") OR TITLE-ABS-KEY("BMS-188667") OR TITLE-ABS-KEY("BMS188667") OR TITLE-ABS-KEY("Belatacept") OR TITLE-ABS-KEY("BMS224818") OR TITLE-ABS-KEY("BMS-224818") OR TITLE-ABS-KEY("BMS 224818") OR TITLE-ABS-KEY("CTLA-4-Ig") OR TITLE-ABS-KEY("CTLA4-Ig Immunoconjugate") OR TITLE-ABS-KEY("CTLA4 Ig Immunoconjugate") OR TITLE-ABS-KEY("Immunoconjugate, CTLA4-Ig") OR TITLE-ABS-KEY("CTLA4-Fc") OR TITLE-ABS-KEY("CTLA4-Ig") OR TITLE-ABS-KEY("Cytotoxic T Lymphocyte-Associated Antigen 4-Immunoglobulin") OR TITLE-ABS-KEY("Cytotoxic T Lymphocyte Associated Antigen 4 Immunoglobulin") OR TITLE-ABS-KEY("LEA29Y") OR TITLE-ABS-KEY("Nulojix")
#4	TITLE-ABS-KEY("anifrolumab") OR TITLE-ABS-KEY("MEDI-546") OR TITLE-ABS-KEY("Saphnelo")
#5	TITLE-ABS-KEY("belimumab") OR TITLE-ABS-KEY("LymphoStat-B") OR TITLE-ABS-KEY("Benlysta") OR TITLE-ABS-KEY("GSK-1550188") OR TITLE-ABS-KEY("GSK1550188") OR TITLE-ABS-KEY("BEL-114333") OR TITLE-ABS-KEY("BEL114333") OR TITLE-ABS-KEY("HGS-1006") OR TITLE-ABS-KEY("HGS1006")

#6	TITLE-ABS-KEY("Interleukin-2") OR TITLE-ABS-KEY("Interleukin 2") OR TITLE-ABS-KEY("IL2") OR TITLE-ABS-KEY("Interleukin II") OR TITLE-ABS-KEY("Interleukine 2") OR TITLE-ABS-KEY("Lymphocyte Mitogenic Factor") OR TITLE-ABS-KEY("Mitogenic Factor, Lymphocyte") OR TITLE-ABS-KEY("T-Cell Growth Factor") OR TITLE-ABS-KEY("T Cell Growth Factor") OR TITLE-ABS-KEY("T-Cell Stimulating Factor") OR TITLE-ABS-KEY("T Cell Stimulating Factor") OR TITLE-ABS-KEY("TCGF") OR TITLE-ABS-KEY("IL-2") OR TITLE-ABS-KEY("Thymocyte Stimulating Factor") OR TITLE-ABS-KEY("RU 49637") OR TITLE-ABS-KEY("RU-49637") OR TITLE-ABS-KEY("RU49637") OR TITLE-ABS-KEY("Ro-23-6019") OR TITLE-ABS-KEY("Ro236019") OR TITLE-ABS-KEY("Ro 23 6019") OR TITLE-ABS-KEY("Ro-236019") OR TITLE-ABS-KEY("Ro 236019")
#7	TITLE-ABS-KEY("Obinutuzumab")
#8	TITLE-ABS-KEY("ocrelizumab") OR TITLE-ABS-KEY("PR 070769") OR TITLE-ABS-KEY("PR-070769") OR TITLE-ABS-KEY("PR070769") OR TITLE-ABS-KEY("R 1594") OR TITLE-ABS-KEY("R-1594") OR TITLE-ABS-KEY("R1594") OR TITLE-ABS-KEY("RG-1594") OR TITLE-ABS-KEY("Ocrevus")
#9	TITLE-ABS-KEY("Rituximab") OR TITLE-ABS-KEY("CD20 Antibody, Rituximab") OR TITLE-ABS-KEY("Rituximab CD20 Antibody") OR TITLE-ABS-KEY("Rituxan") OR TITLE-ABS-KEY("IDEC-C2B8 Antibody") OR TITLE-ABS-KEY("IDEC C2B8 Antibody") OR TITLE-ABS-KEY("IDEC-C2B8") OR TITLE-ABS-KEY("IDEC C2B8") OR TITLE-ABS-KEY("Mabthera") OR TITLE-ABS-KEY("GP2013")
#10	TITLE-ABS-KEY("Biological Therapy") OR TITLE-ABS-KEY("Biological Therapies") OR TITLE-ABS-KEY("Therapies, Biological") OR TITLE-ABS-KEY("Therapy, Biological") OR TITLE-ABS-KEY("Biologic Therapy") OR TITLE-ABS-KEY("Biologic Therapies") OR TITLE-ABS-KEY("Therapies, Biologic") OR TITLE-ABS-KEY("Therapy, Biologic") OR TITLE-ABS-KEY("Biotherapy") OR TITLE-ABS-KEY("Biotherapies")
#11	TITLE-ABS-KEY("epratuzumab")
#12	TITLE-ABS-KEY("telitacicept")
#13	TITLE-ABS-KEY("Azathioprine") OR TITLE-ABS-KEY("Azothioprine") OR TITLE-ABS-KEY("Imuran") OR TITLE-ABS-KEY("Immuran") OR TITLE-ABS-KEY("Imurel") OR TITLE-ABS-KEY("Azathioprine Sulfate") OR TITLE-ABS-KEY("Azathioprine Sodium") OR TITLE-ABS-KEY("Sodium, Azathioprine") OR TITLE-ABS-KEY("Azathioprine Sodium Salt")
#14	TITLE-ABS-KEY("Cyclophosphamide") OR TITLE-ABS-KEY("Cytoxan") OR TITLE-ABS-KEY("Endoxan") OR TITLE-ABS-KEY("Neosar") OR TITLE-ABS-

	KEY("Procytox") OR TITLE-ABS-KEY("Sendoxan") OR TITLE-ABS-KEY("Cyclophosphamide, TITLE-ABS-KEY(S)-Isomer") OR TITLE-ABS-KEY("Cyclophosphamide, TITLE-ABS-KEY(R)-Isomer") OR TITLE-ABS-KEY("NSC-26271") OR TITLE-ABS-KEY("NSC26271") OR TITLE-ABS-KEY("NSC 26271") OR TITLE-ABS-KEY("B-518") OR TITLE-ABS-KEY("B518") OR TITLE-ABS-KEY("B 518") OR TITLE-ABS-KEY("Cytophosphan") OR TITLE-ABS-KEY("Cyclophosphamide Anhydrous") OR TITLE-ABS-KEY("Cyclophosphane") OR TITLE-ABS-KEY("Cytophosphane") OR TITLE-ABS-KEY("Cyclophosphamide Monohydrate") OR TITLE-ABS-KEY(" TITLE-ABS-KEY(+,-)-2- TITLE-ABS-KEY(bis TITLE-ABS-KEY(2-Chloroethyl)amino)tetrahydro-2H-1,3,2-oxazaphosphorine 2-Oxide Monohydrate")
#15	TITLE-ABS-KEY("Mycophenolic Acid") OR TITLE-ABS-KEY("Cellcept") OR TITLE-ABS-KEY("Mycophenolate Mofetil") OR TITLE-ABS-KEY("Mofetil, Mycophenolate") OR TITLE-ABS-KEY("Mycophenolic Acid Morpholinoethyl Ester") OR TITLE-ABS-KEY("RS 61443") OR TITLE-ABS-KEY("RS-61443") OR TITLE-ABS-KEY("RS61443") OR TITLE-ABS-KEY("Mycophenolate Mofetil Hydrochloride") OR TITLE-ABS-KEY("Mofetil Hydrochloride, Mycophenolate") OR TITLE-ABS-KEY("Myfortic") OR TITLE-ABS-KEY("Mycophenolate Sodium") OR TITLE-ABS-KEY("Sodium Mycophenolate") OR TITLE-ABS-KEY("Mycophenolate, Sodium")
#16	TITLE-ABS-KEY("Tacrolimus") OR TITLE-ABS-KEY("Prograf") OR TITLE-ABS-KEY("Prograft") OR TITLE-ABS-KEY("Anhydrous Tacrolimus") OR TITLE-ABS-KEY("Tacrolimus, Anhydrous") OR TITLE-ABS-KEY("Tacrolimus Anhydrous") OR TITLE-ABS-KEY("Anhydrous, Tacrolimus") OR TITLE-ABS-KEY("FK-506") OR TITLE-ABS-KEY("FK 506") OR TITLE-ABS-KEY("FK506") OR TITLE-ABS-KEY("FR-900506") OR TITLE-ABS-KEY("FR900506") OR TITLE-ABS-KEY("FR 900506")
#17	TITLE-ABS-KEY("voclosporin") OR TITLE-ABS-KEY("ISATX247") OR TITLE-ABS-KEY("ISA TITLE-ABS-KEY(TX)247") OR TITLE-ABS-KEY("ISA 247") OR TITLE-ABS-KEY("ISA-247") OR TITLE-ABS-KEY("lupkynis")
#18	TITLE-ABS-KEY("Glucocorticoids") OR TITLE-ABS-KEY("Glucocorticoid") OR TITLE-ABS-KEY("Glucocorticoid Effect") OR TITLE-ABS-KEY("Effect, Glucocorticoid") OR TITLE-ABS-KEY("Glucorticoid Effects") OR TITLE-ABS-KEY("Effects, Glucorticoid")
#19	"Randomized Controlled Trials as Topic" OR "Clinical Trial")
#20	#2 OR #3 OR #4 OR #5 OR #6 OR #7 OR #8 OR #9 OR #10 OR #11 OR #12 OR #13 OR #14 OR #15 OR #16 OR #17 OR #18 OR #19
#21	#1 AND #19 AND #20

Supplementary Data 2: Adult patients meeting the LN diagnostic criteria based on the 1982 American College of Rheumatology LN classification criteria updated in 1997, which include:

- Urine protein-to-creatinine ratio (UPCR) > 1 from a 24-hour urine collection;

- Estimated glomerular filtration rate (eGFR) ≥ 30 mL/min/1.73 m2;

- Histological findings consistent with International Society of Nephrology and Renal Pathology Society Class III (focal LN) or Class IV (diffuse LN) LN, with or without Class V (membranous LN), or pure Class V LN within 6 months of disease onset;

- Positive autoantibody status.

2 Supplementary Figures and Tables

2.1 Supplementary Figures

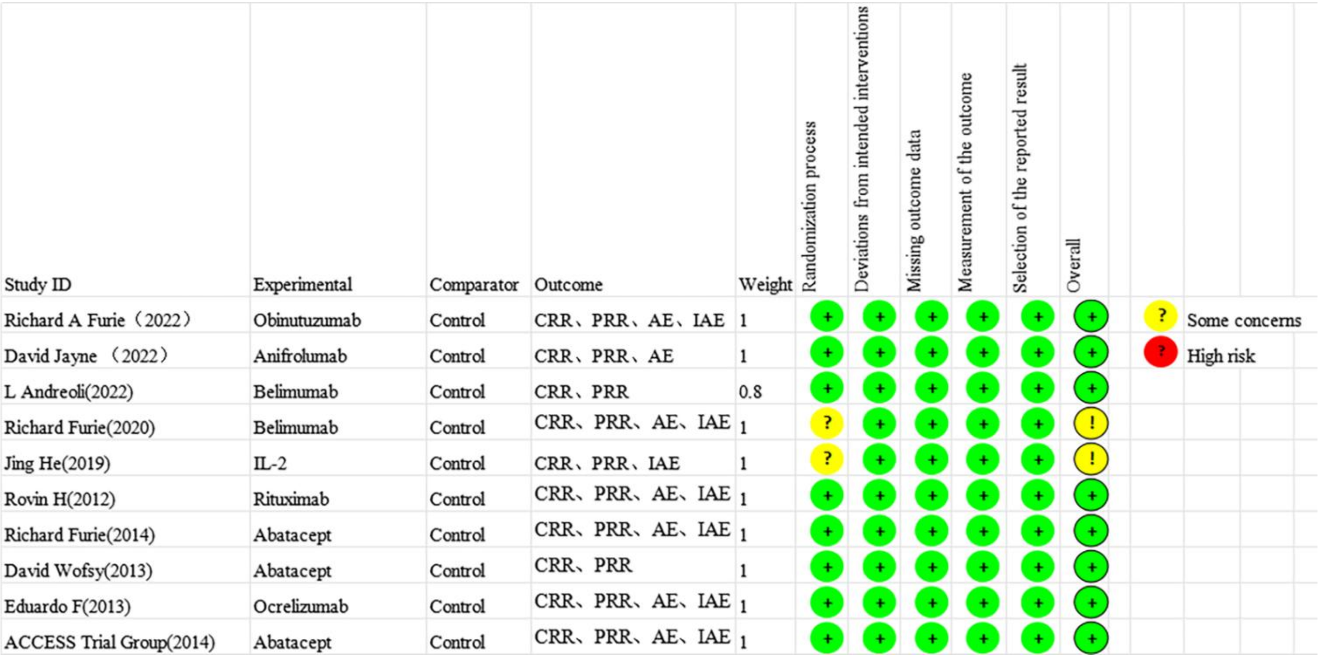

Study ID	Experimental	Comparator	Outcome	Weight	Randomization process	Deviations from intended interventions	Missing outcome data	Measurement of the outcome	Selection of the reported result	Overall
Richard A Furie（2022）	Obinutuzumab	Control	CRR、PRR、AE、IAE	1	+	+	+	+	+	+
David Jayne （2022）	Anifrolumab	Control	CRR、PRR、AE	1	+	+	+	+	+	+
L Andreoli(2022)	Belimumab	Control	CRR、PRR	0.8	+	+	+	+	+	+
Richard Furie(2020)	Belimumab	Control	CRR、PRR、AE、IAE	1	?	+	+	+	+	!
Jing He(2019)	IL-2	Control	CRR、PRR、IAE	1	?	+	+	+	+	!
Rovin H(2012)	Rituximab	Control	CRR、PRR、AE、IAE	1	+	+	+	+	+	+
Richard Furie(2014)	Abatacept	Control	CRR、PRR、AE、IAE	1	+	+	+	+	+	+
David Wofsy(2013)	Abatacept	Control	CRR、PRR	1	+	+	+	+	+	+
Eduardo F(2013)	Ocrelizumab	Control	CRR、PRR、AE、IAE	1	+	+	+	+	+	+
ACCESS Trial Group(2014)	Abatacept	Control	CRR、PRR、AE、IAE	1	+	+	+	+	+	+

Supplementary Figure 1. Summary of quality assessments using Cochrane Risk of Bias Tool.

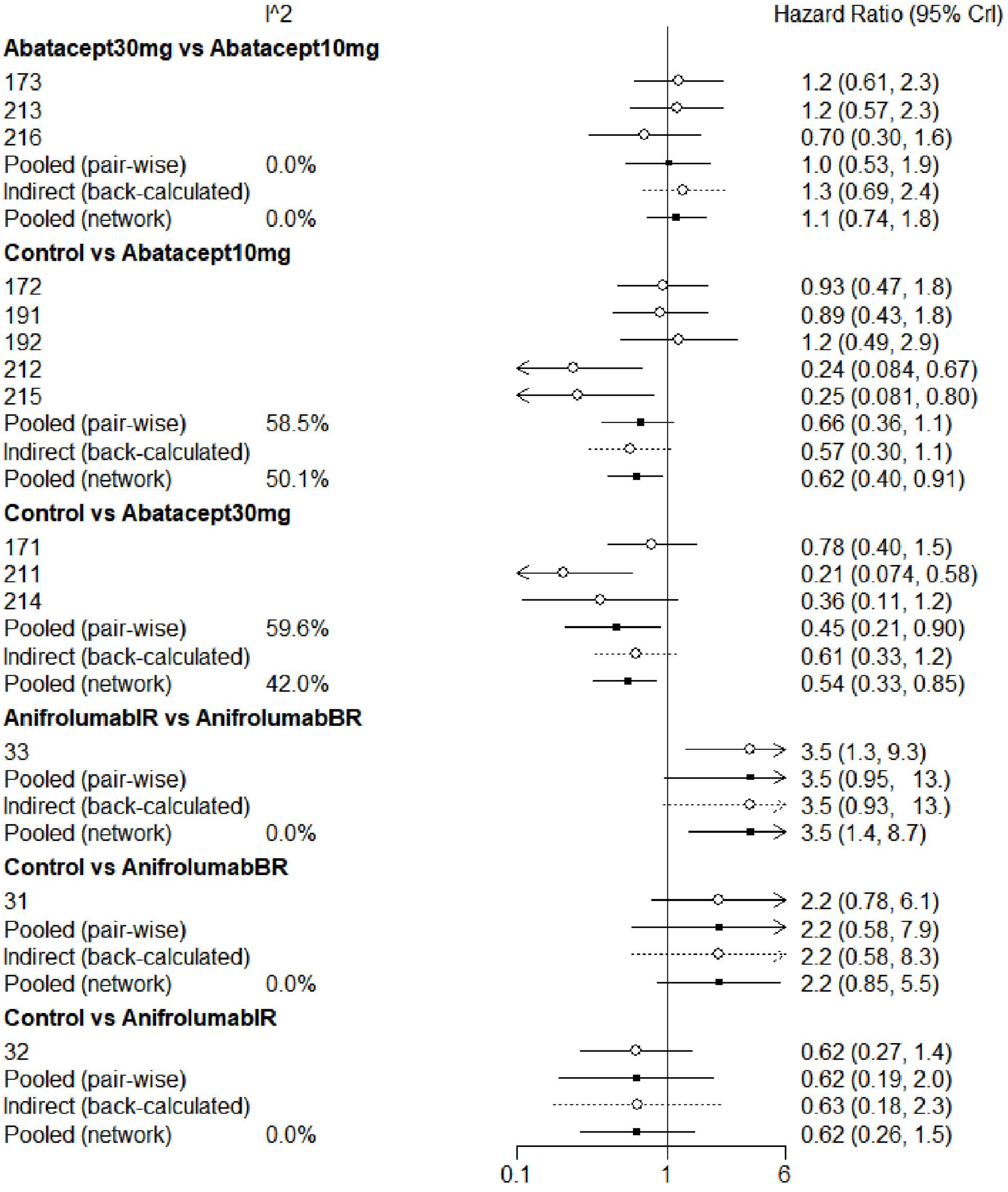

Supplementary Figure 2A. Inconsistency and consistency test on CRR by Bayesian network meta-analysis.

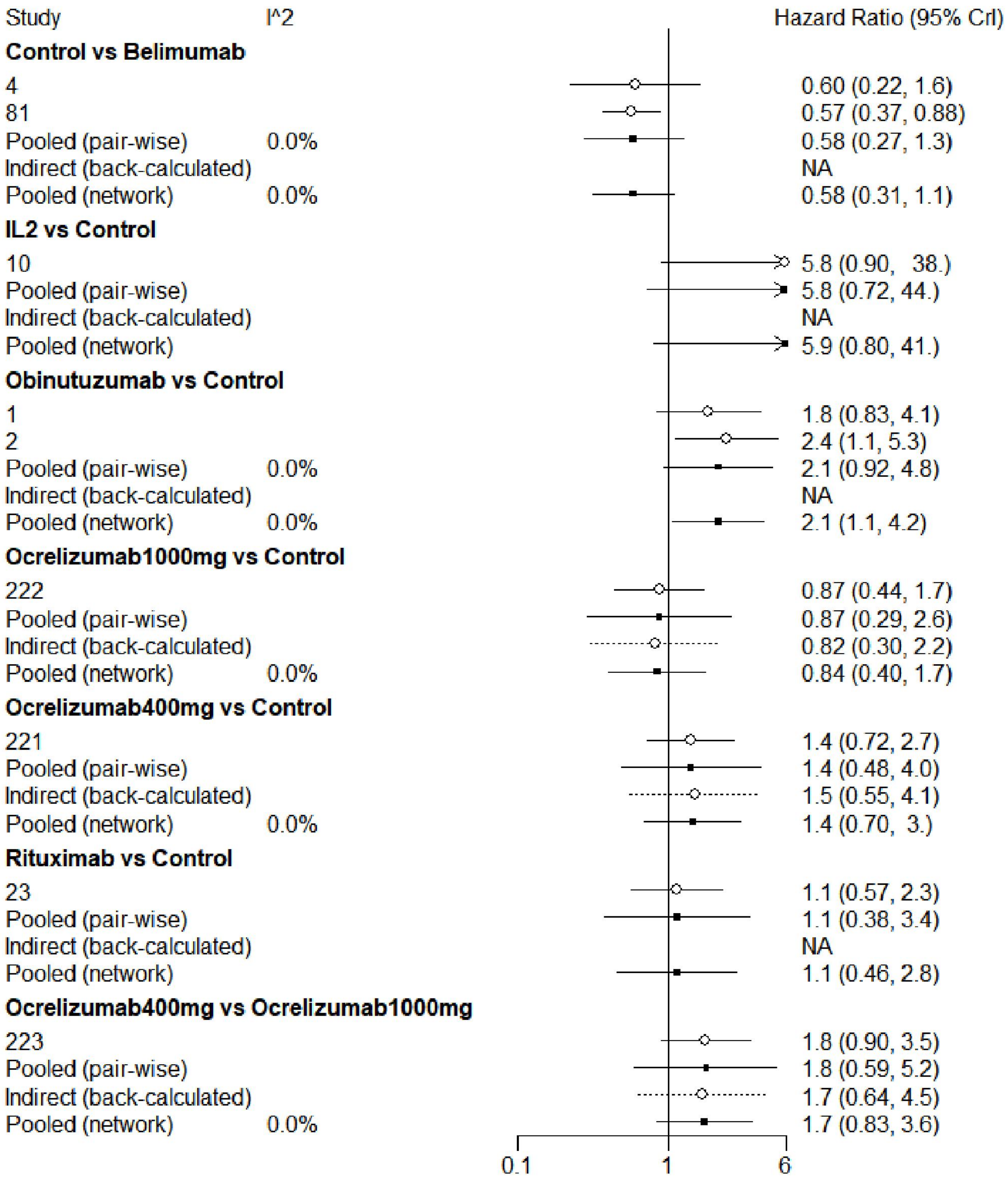

Supplementary Figure 2B. Inconsistency and consistency test on CRR by Bayesian network meta-analysis.

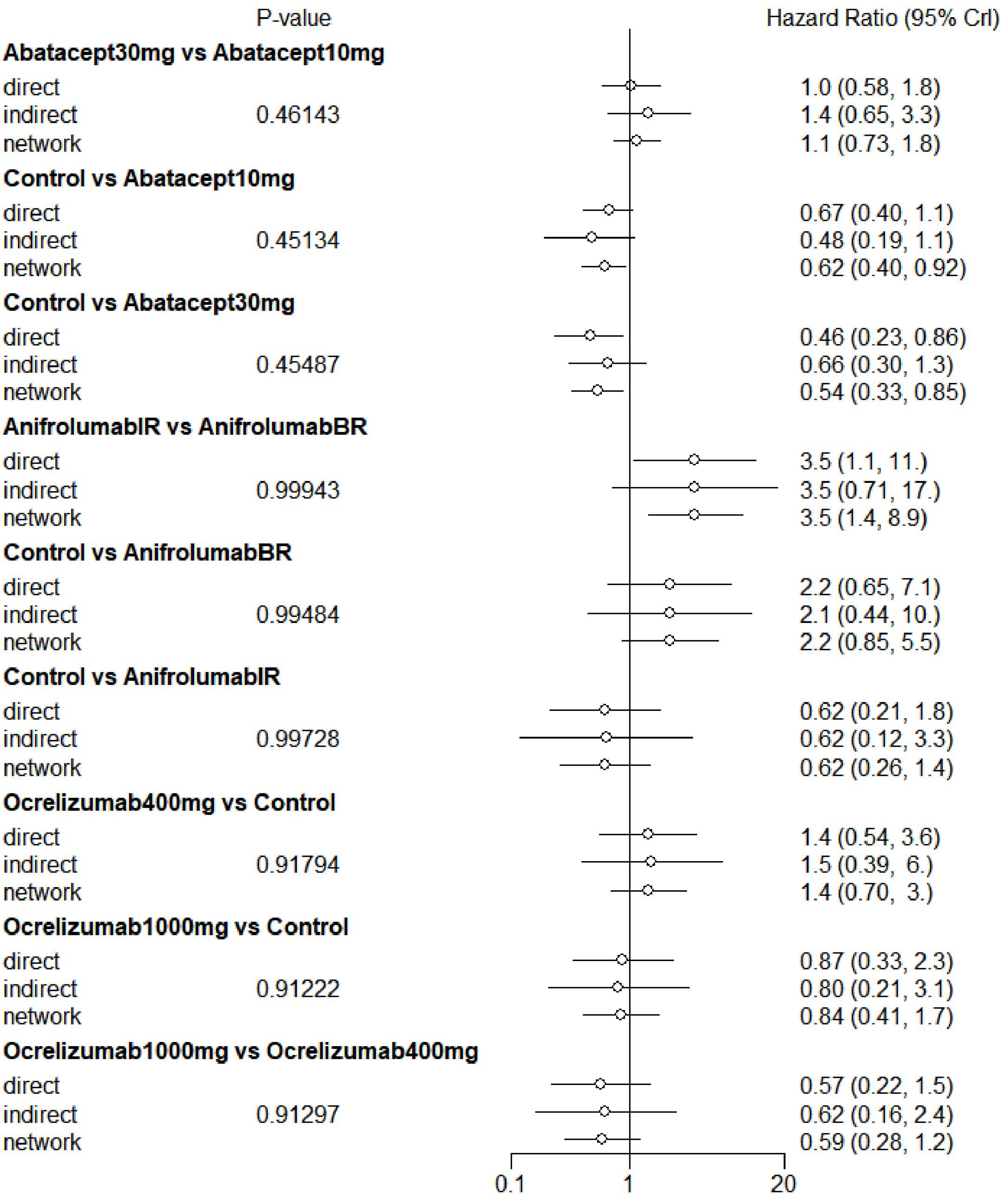

Supplementary Figure 3. Inconsistency analysis of CRR.

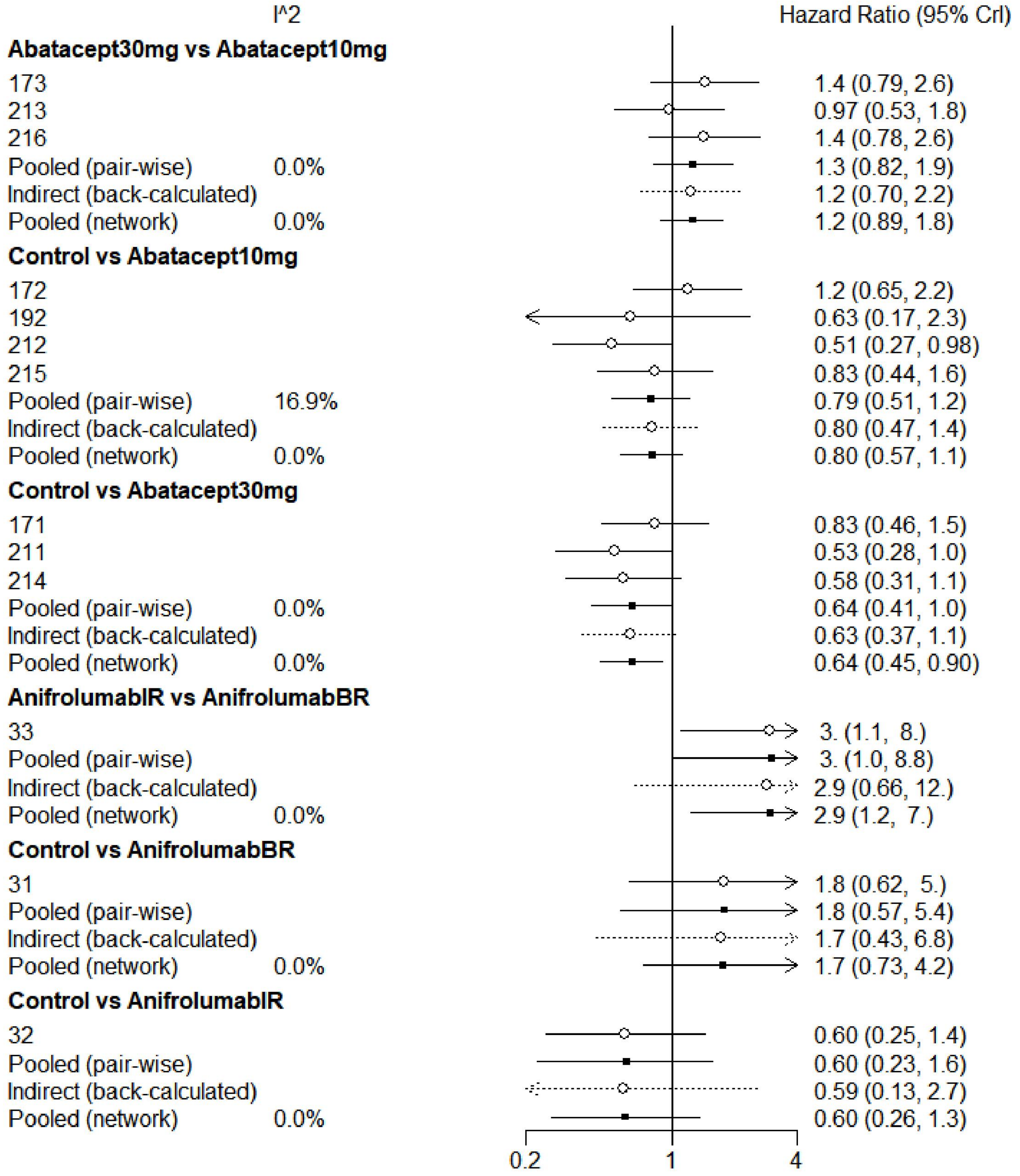

Supplementary Figure 4A. Inconsistency and consistency test on PRR by Bayesian network meta-analysis.

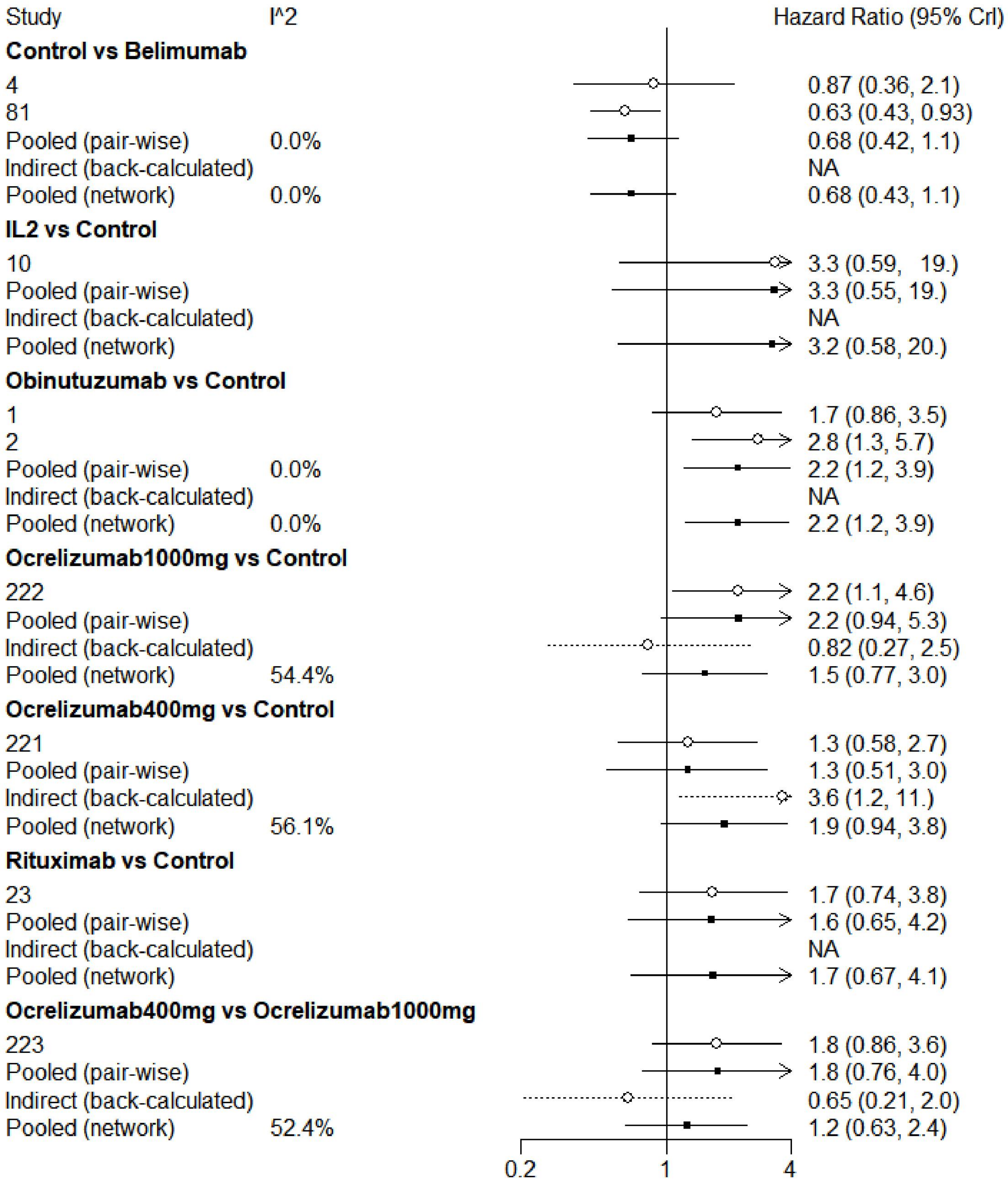

Supplementary Figure 4B. Inconsistency and consistency test on PRR by Bayesian network meta-analysis.

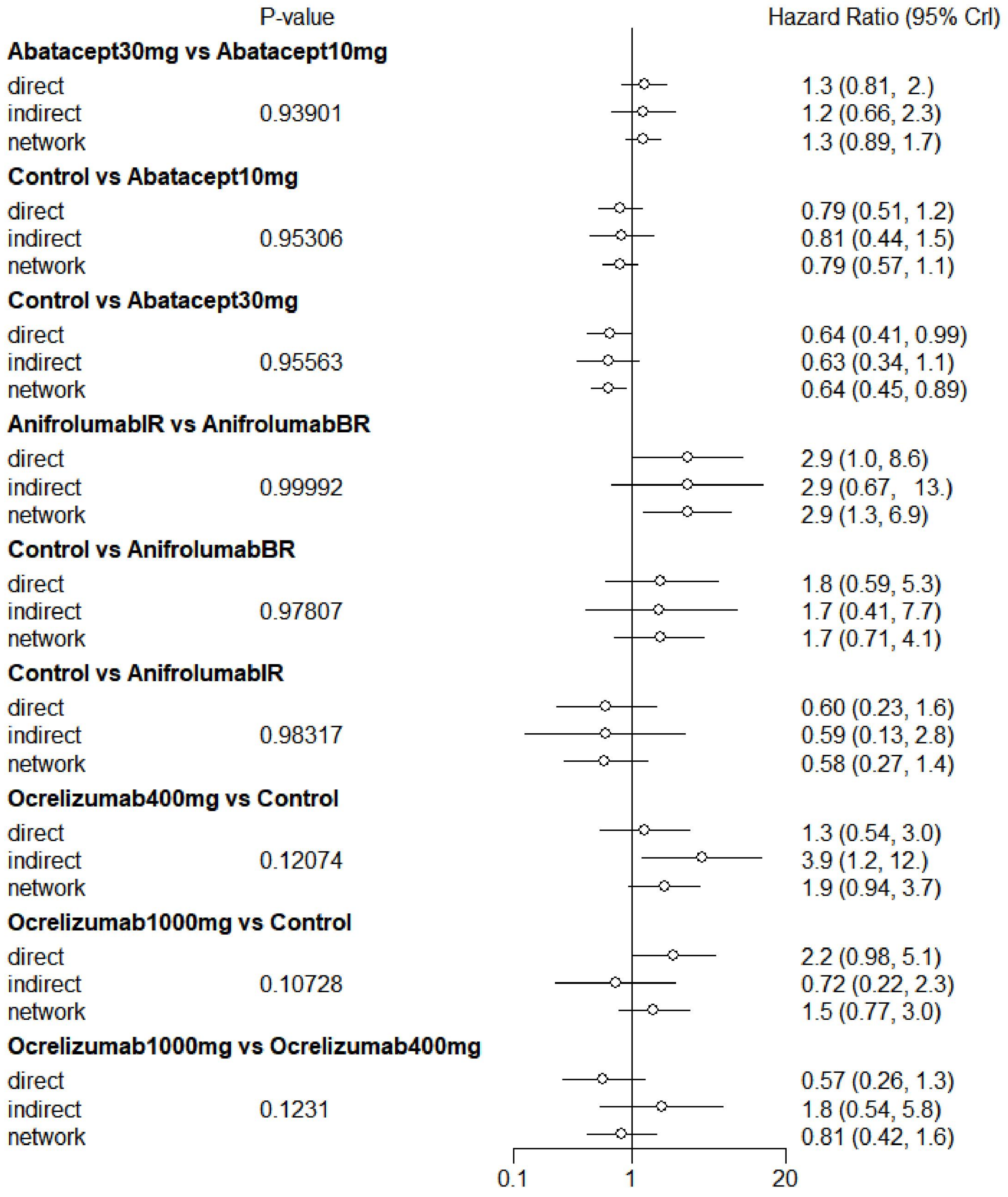

Supplementary Figure 5. Inconsistency analysis of PRR.

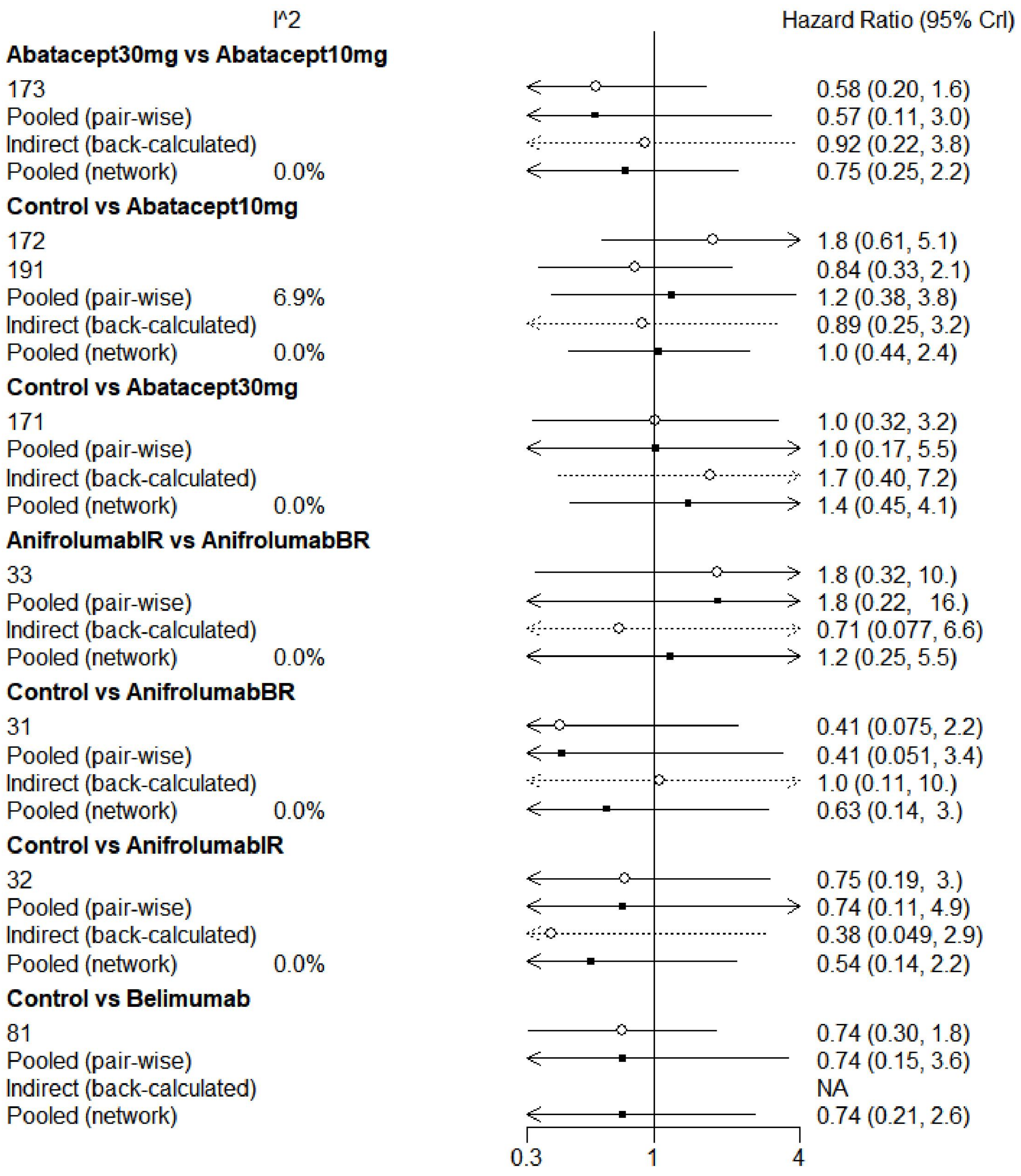

Supplementary Figure 6A. Inconsistency and consistency test on AE by Bayesian network meta-analysis.

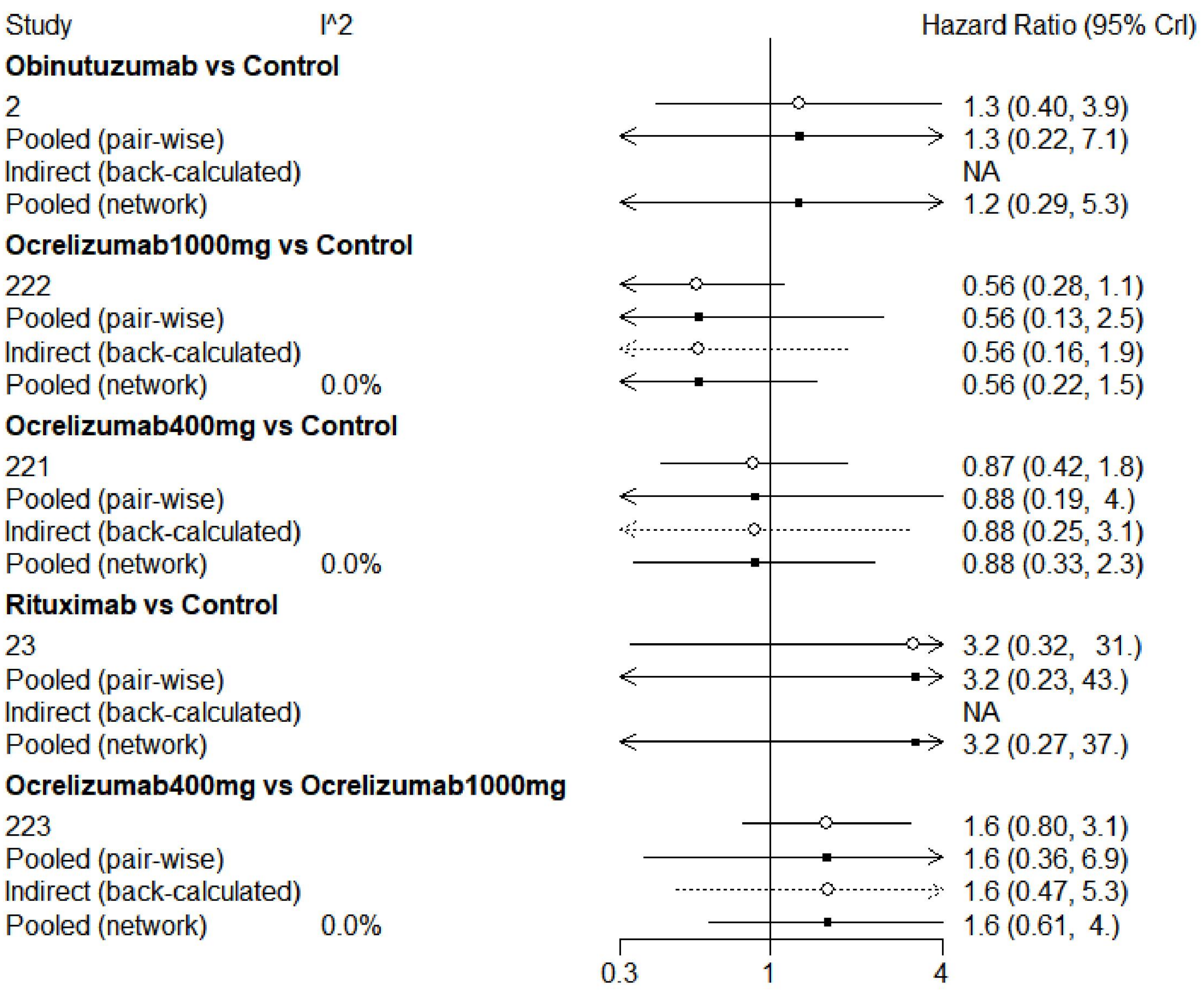

Supplementary Figure 6B. Inconsistency and consistency test on AE by Bayesian network meta-analysis.

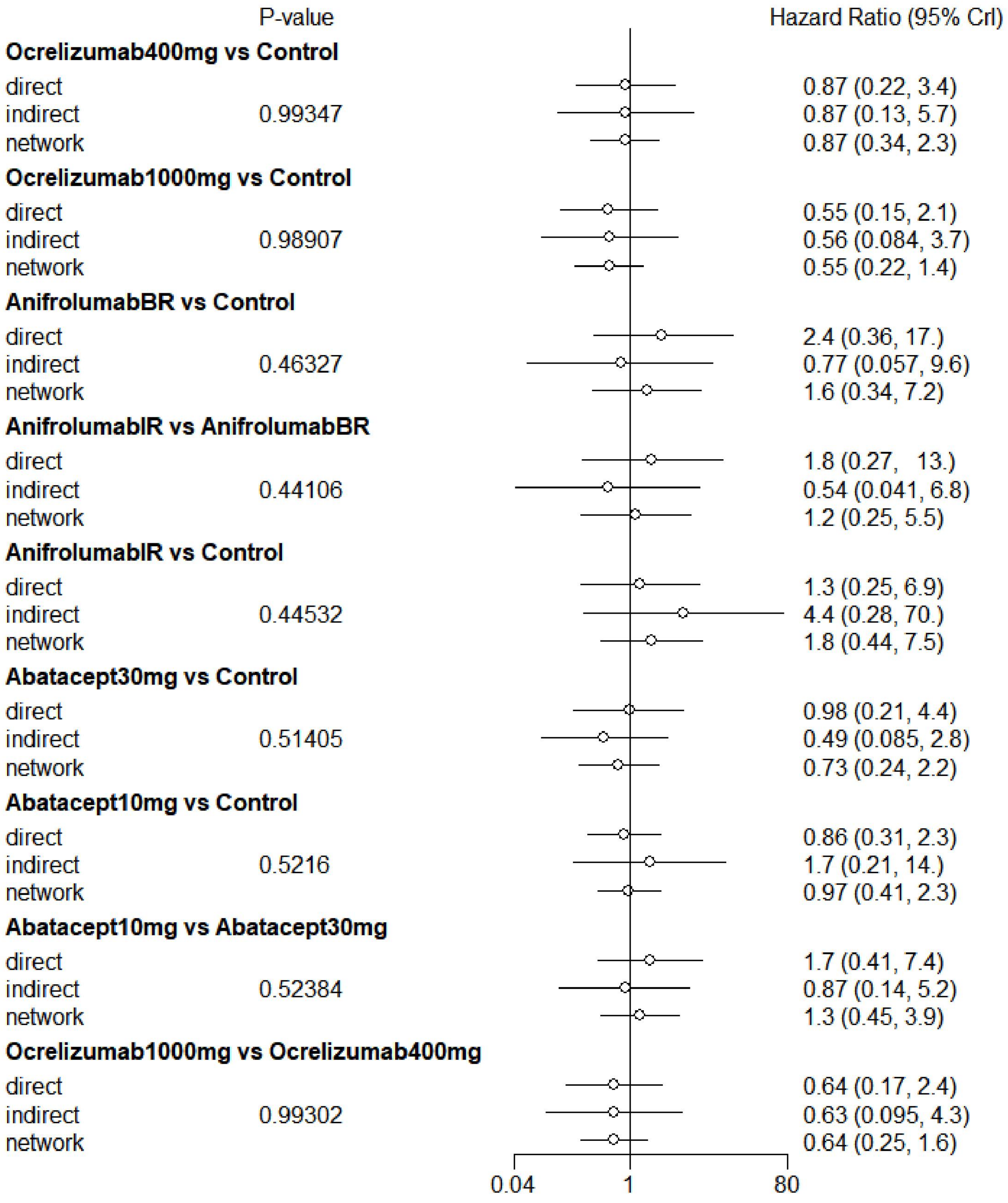

Supplementary Figure 7. Inconsistency analysis of AE.

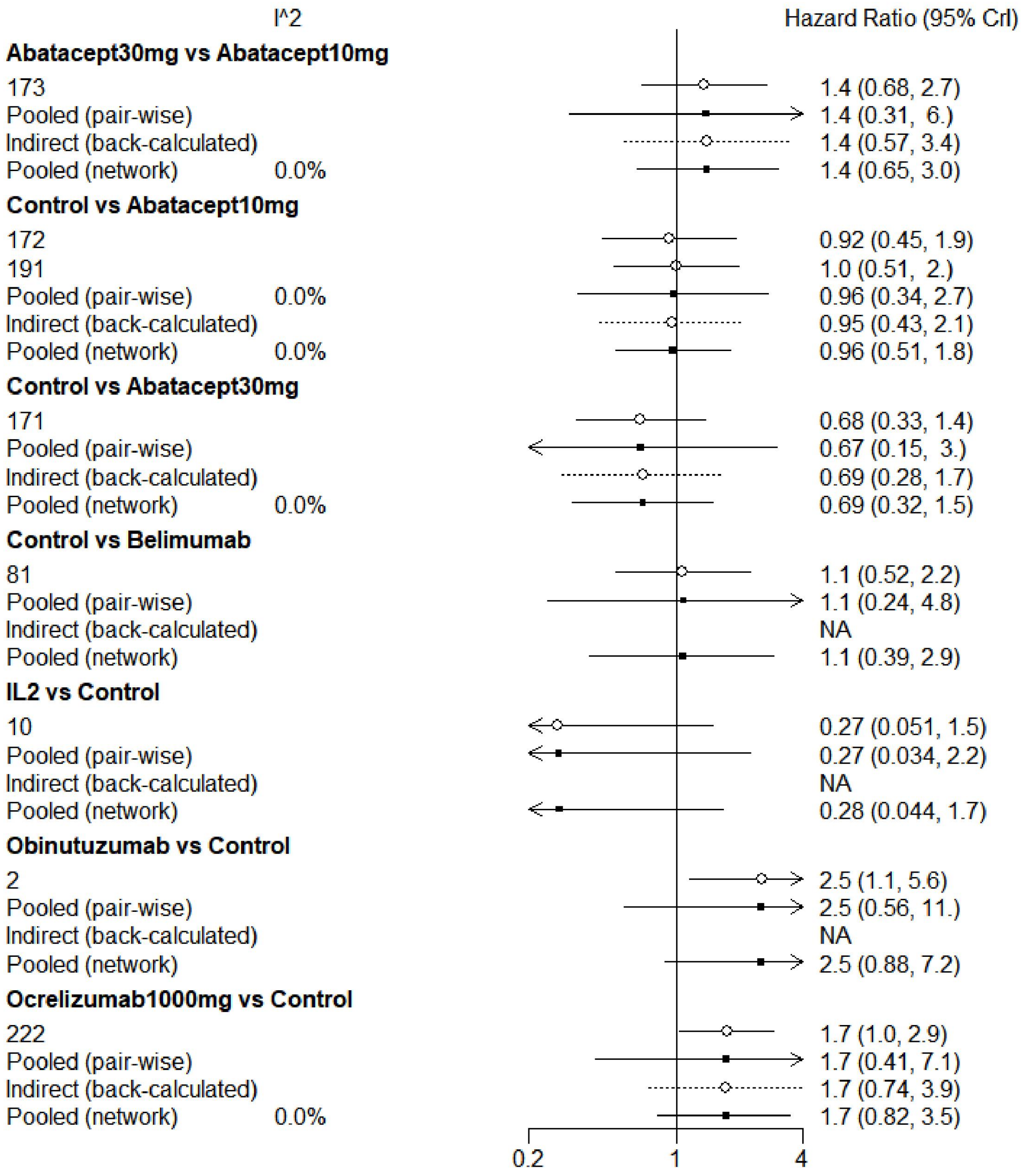

Supplementary Figure 8A. Inconsistency and consistency test on IAE by Bayesian network meta-analysis.

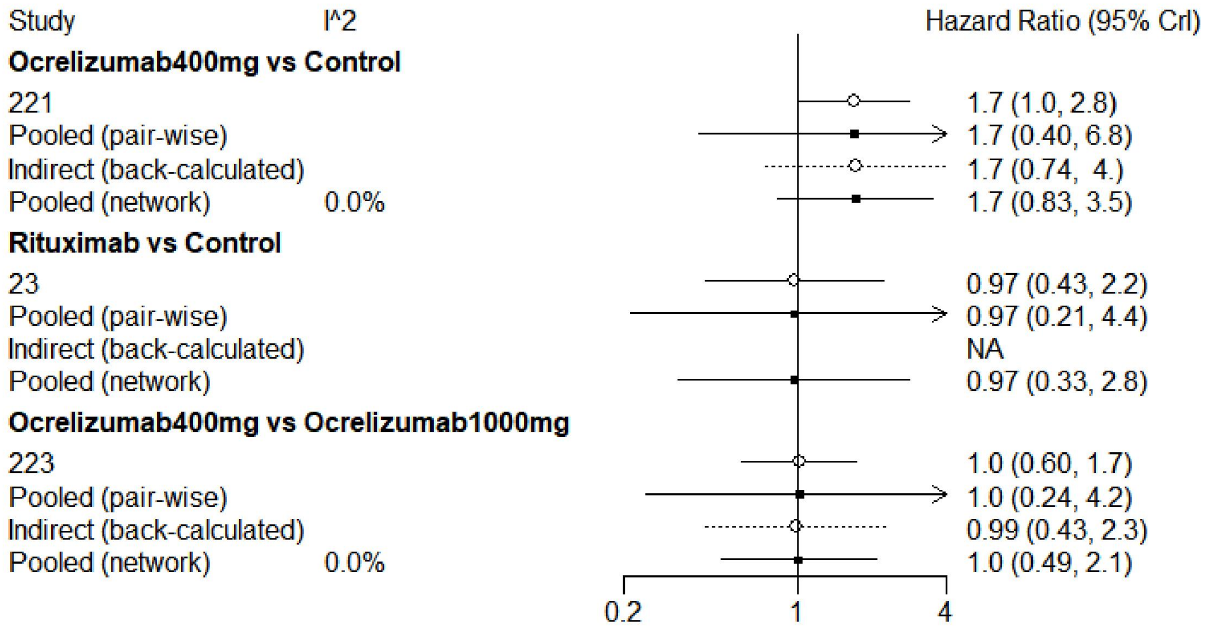

Supplementary Figure 8B. Inconsistency and consistency test on IAE by Bayesian network meta-analysis.

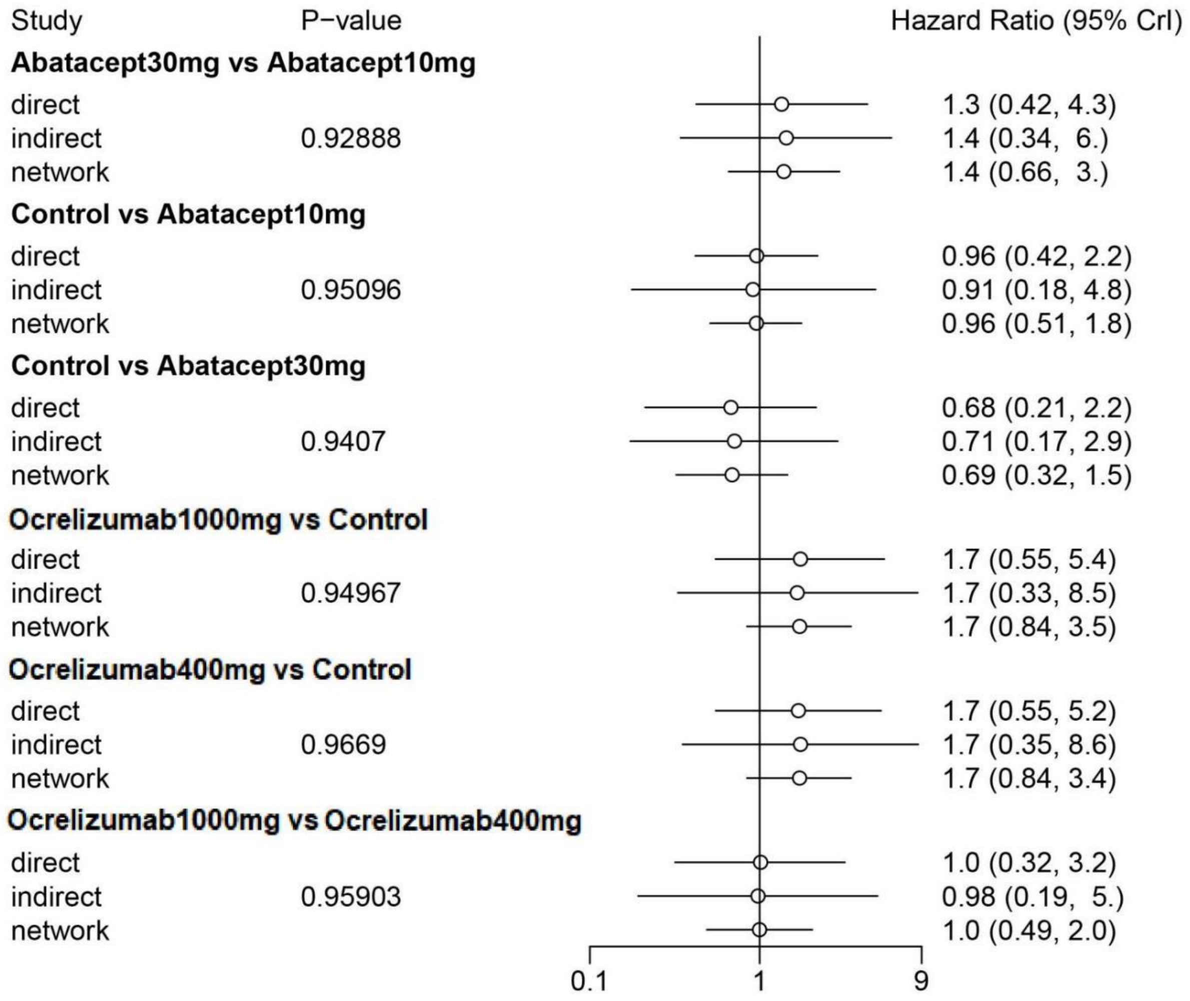

Supplementary Figure 9. Inconsistency analysis of IAE.

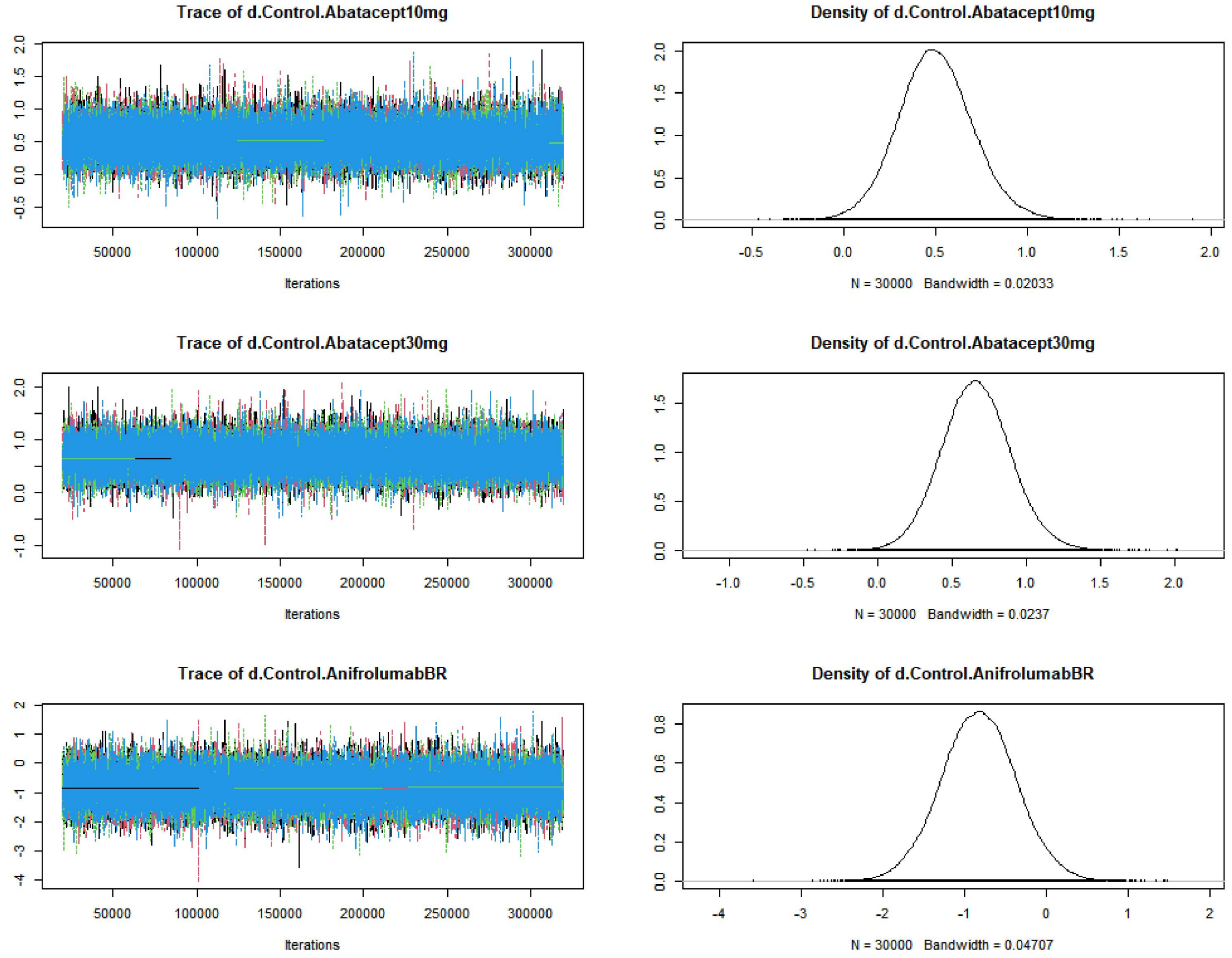

Supplementary Figure 10A. Traces plots and density plots of pairwise comparisons on CRR by Bayesian network meta-regression analyses.

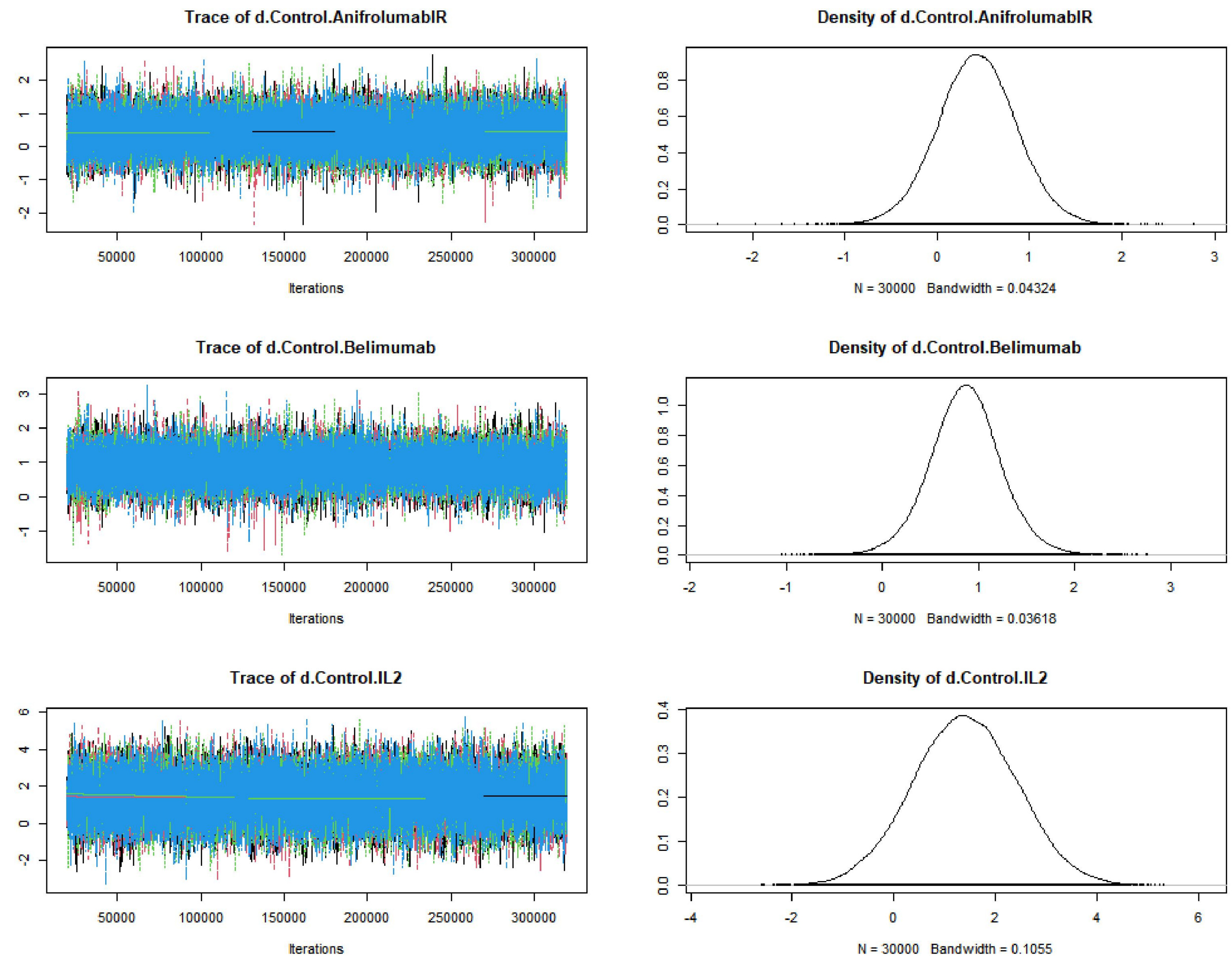

Supplementary Figure 10B. Traces plots and density plots of pairwise comparisons on CRR by Bayesian network meta-regression analyses.

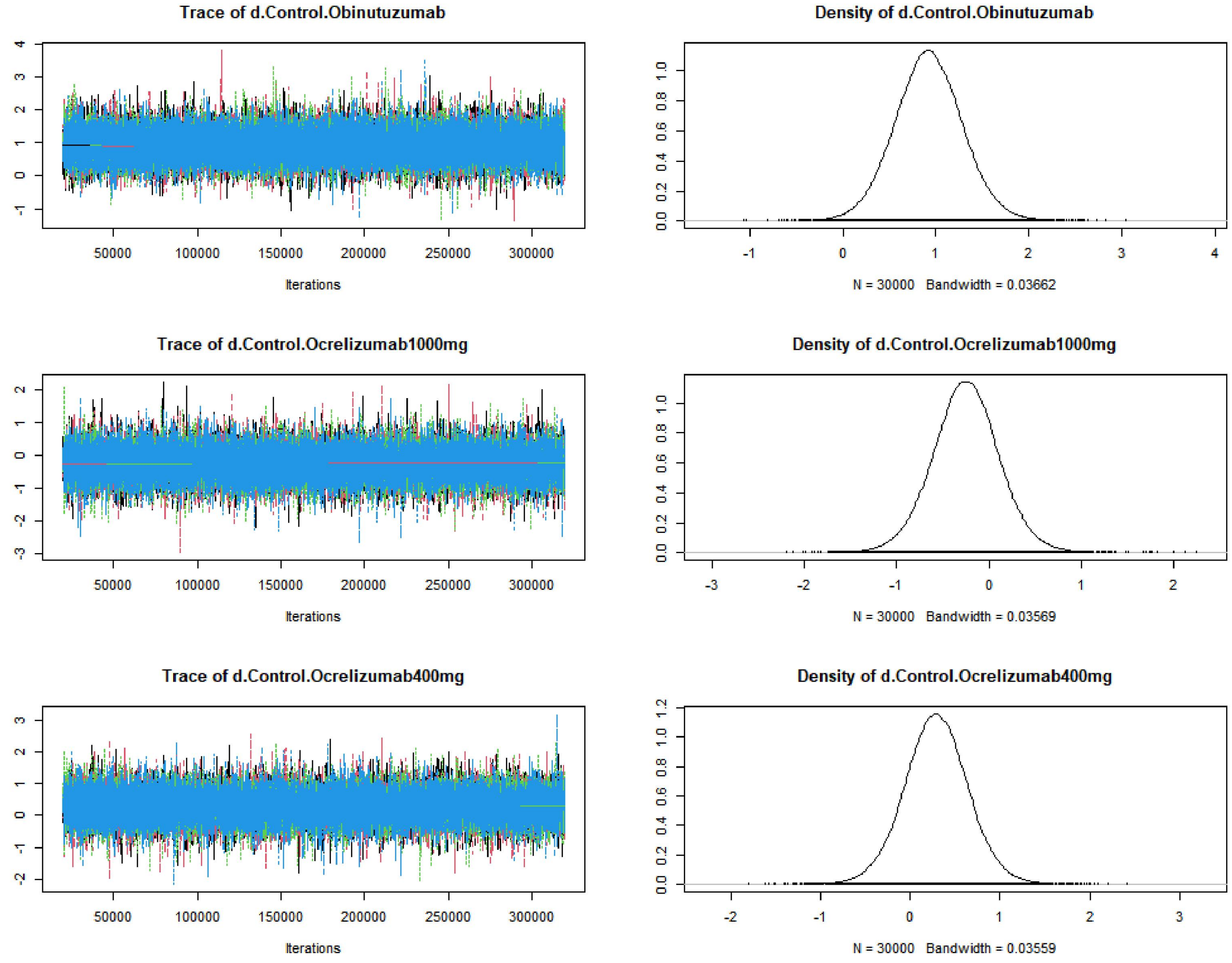

Supplementary Figure 10C. Traces plots and density plots of pairwise comparisons on CRR by Bayesian network meta-regression analyses.

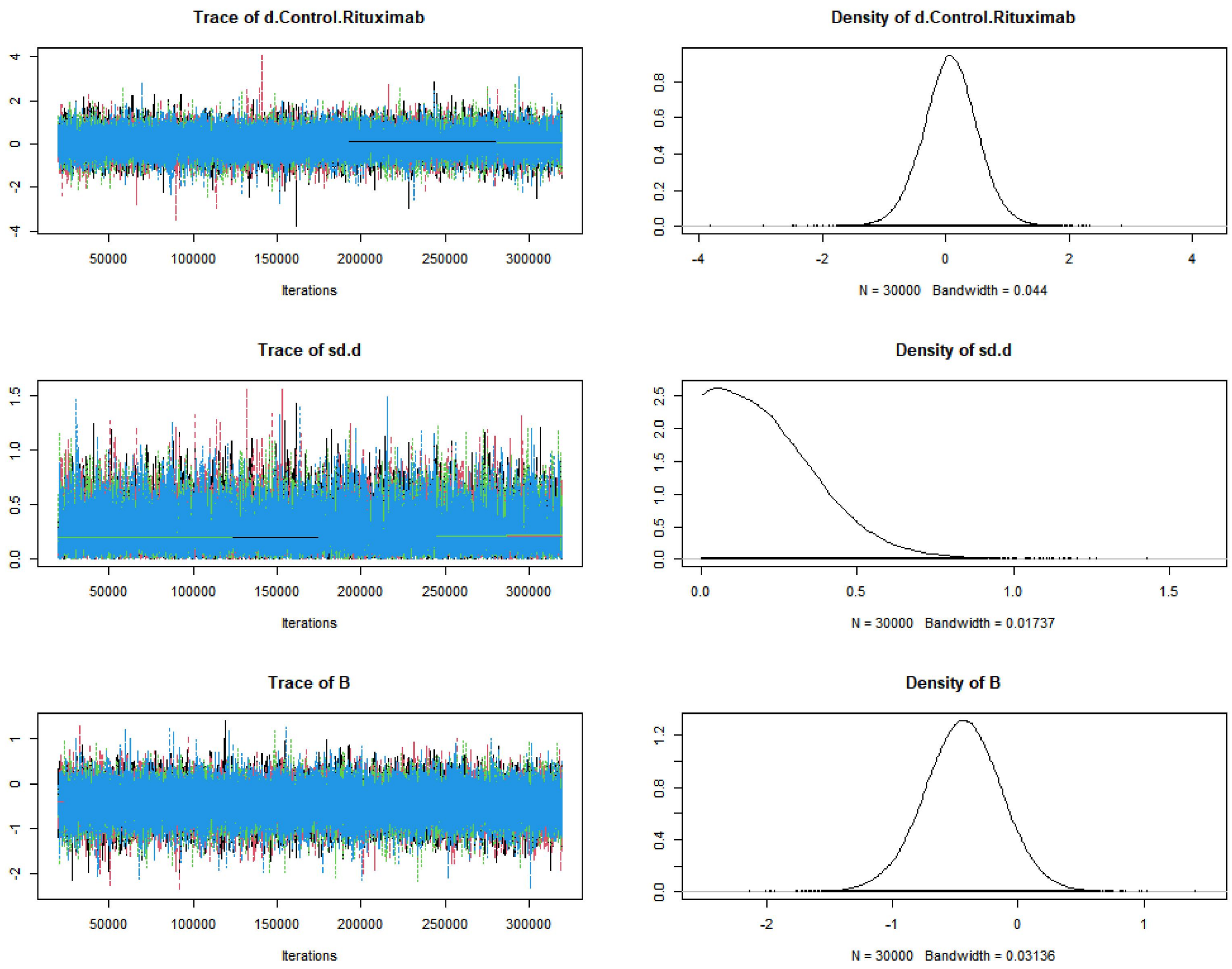

Supplementary Figure 10D. Traces plots and density plots of pairwise comparisons on CRR by Bayesian network meta-regression analyses.

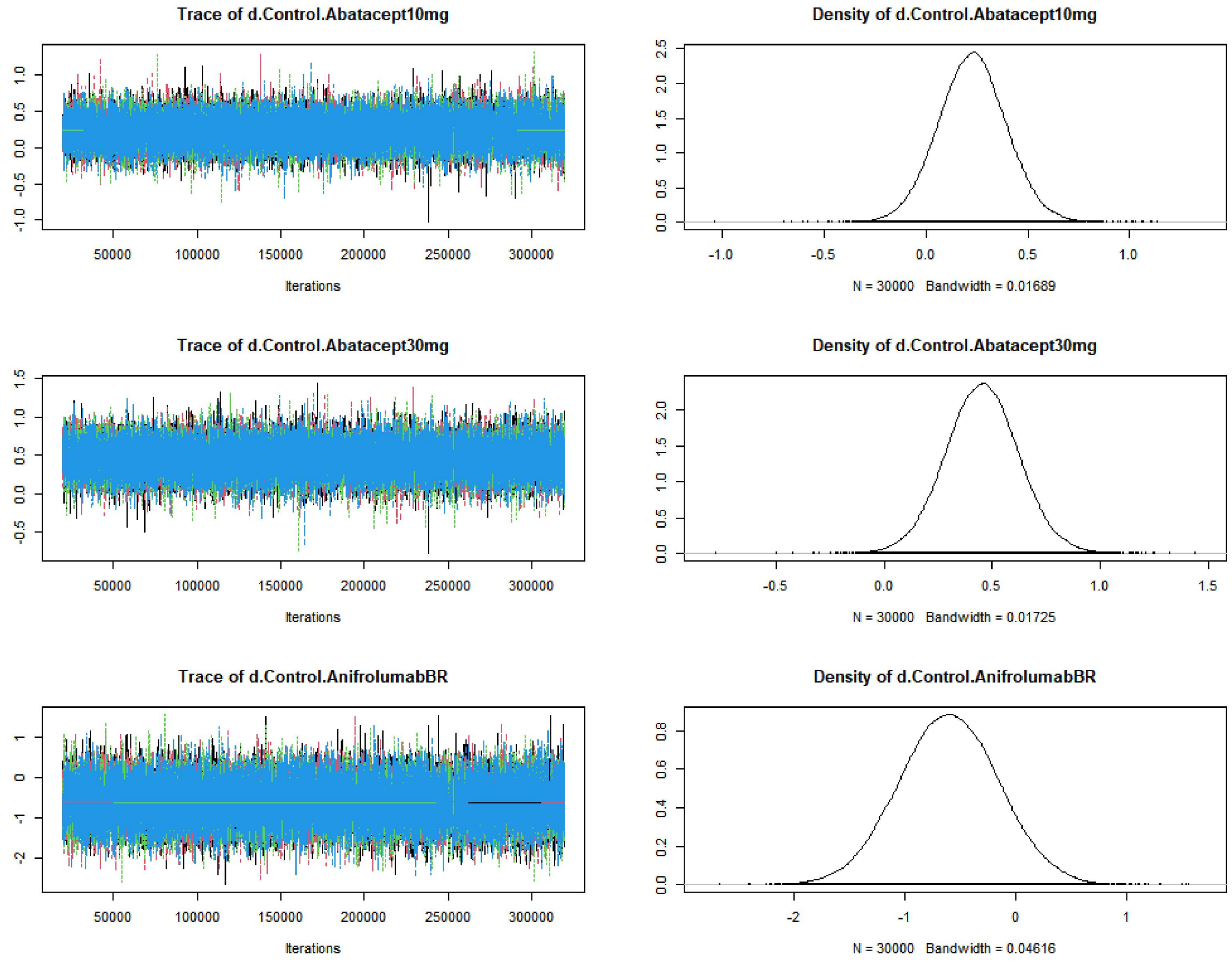

Supplementary Figure 11A. Traces plots and density plots of pairwise comparisons on PRR by Bayesian network meta-regression analyses.

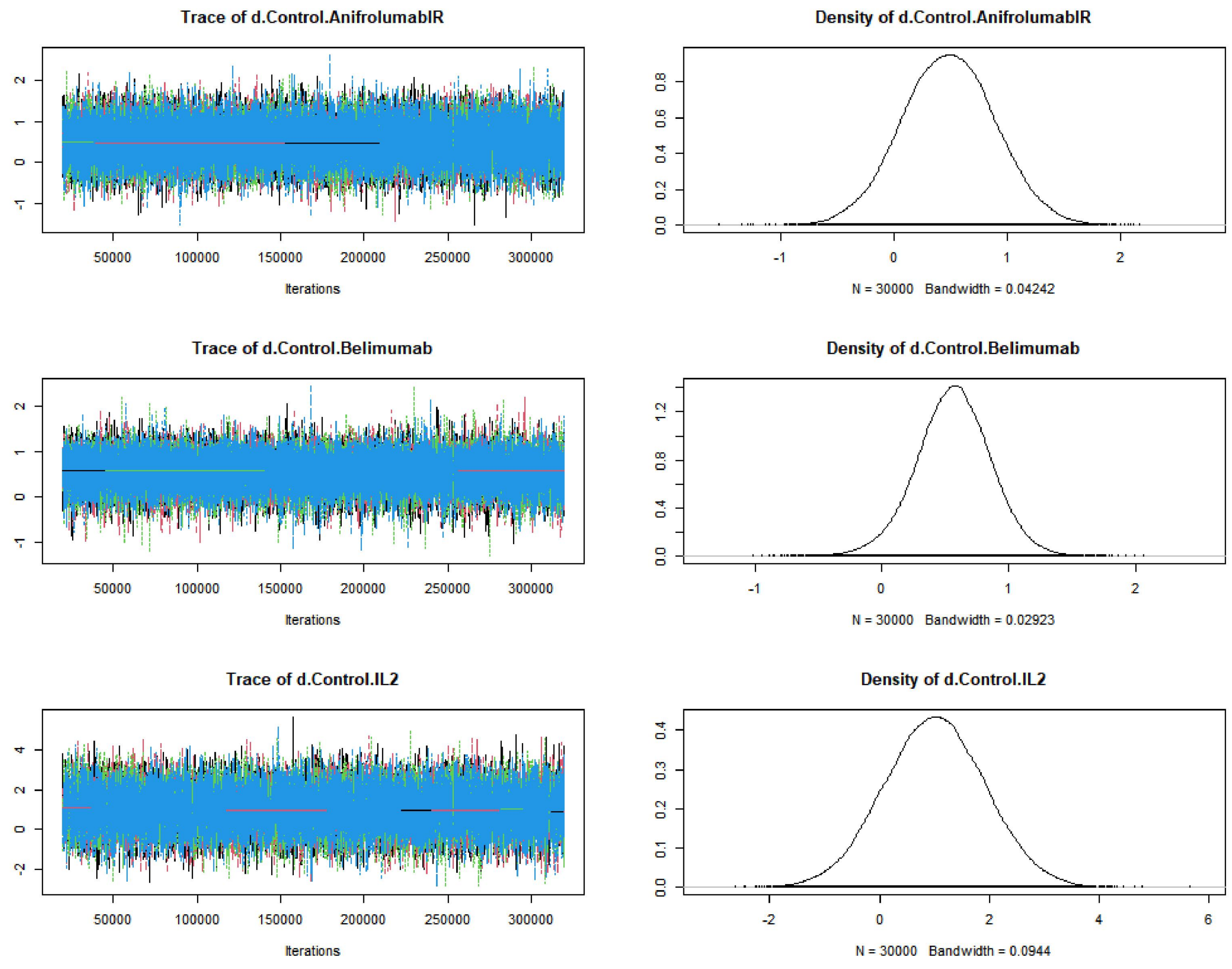

Supplementary Figure 11B. Traces plots and density plots of pairwise comparisons on PRR by Bayesian network meta-regression analyses.

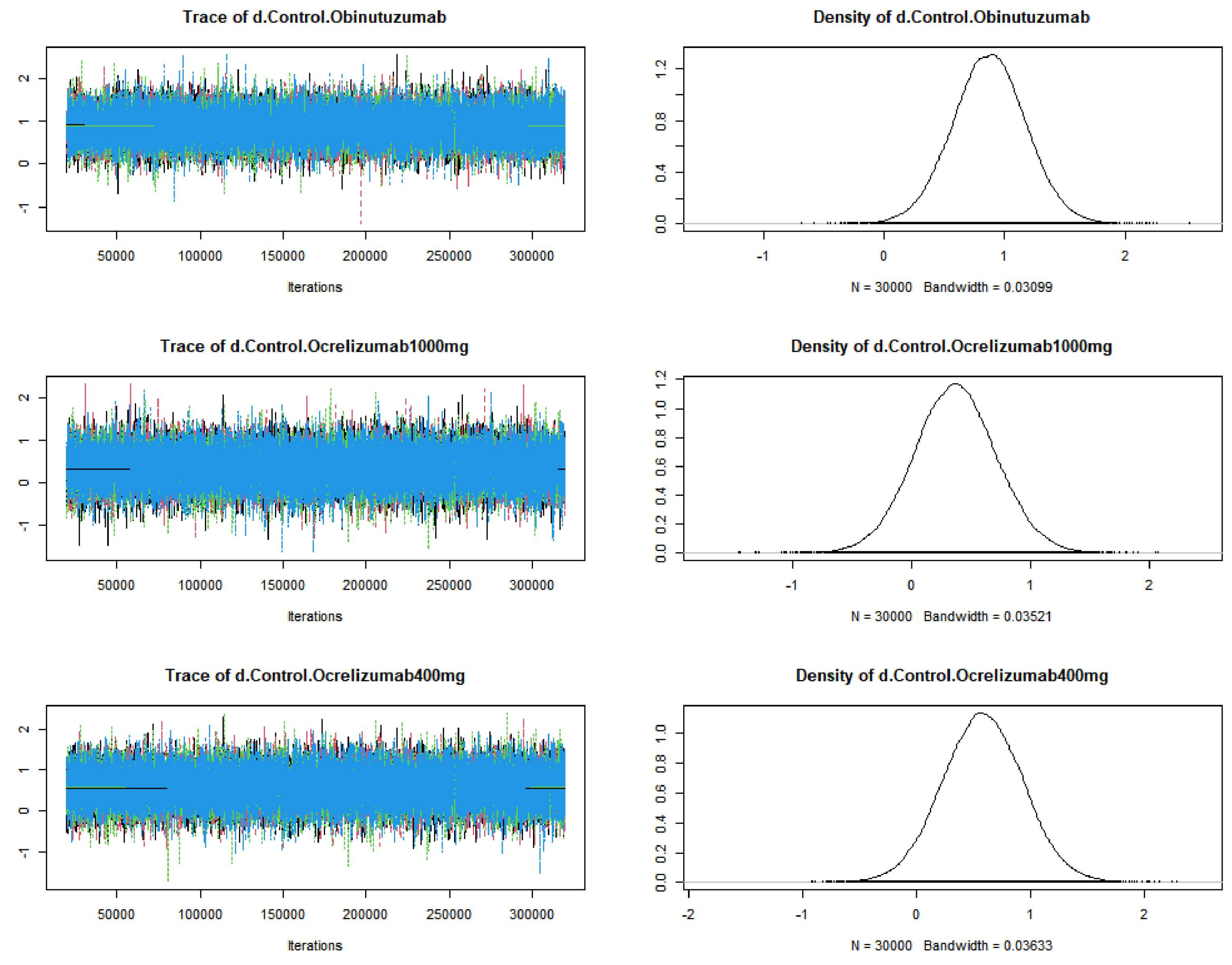

Supplementary Figure 11C. Traces plots and density plots of pairwise comparisons on PRR by Bayesian network meta-regression analyses.

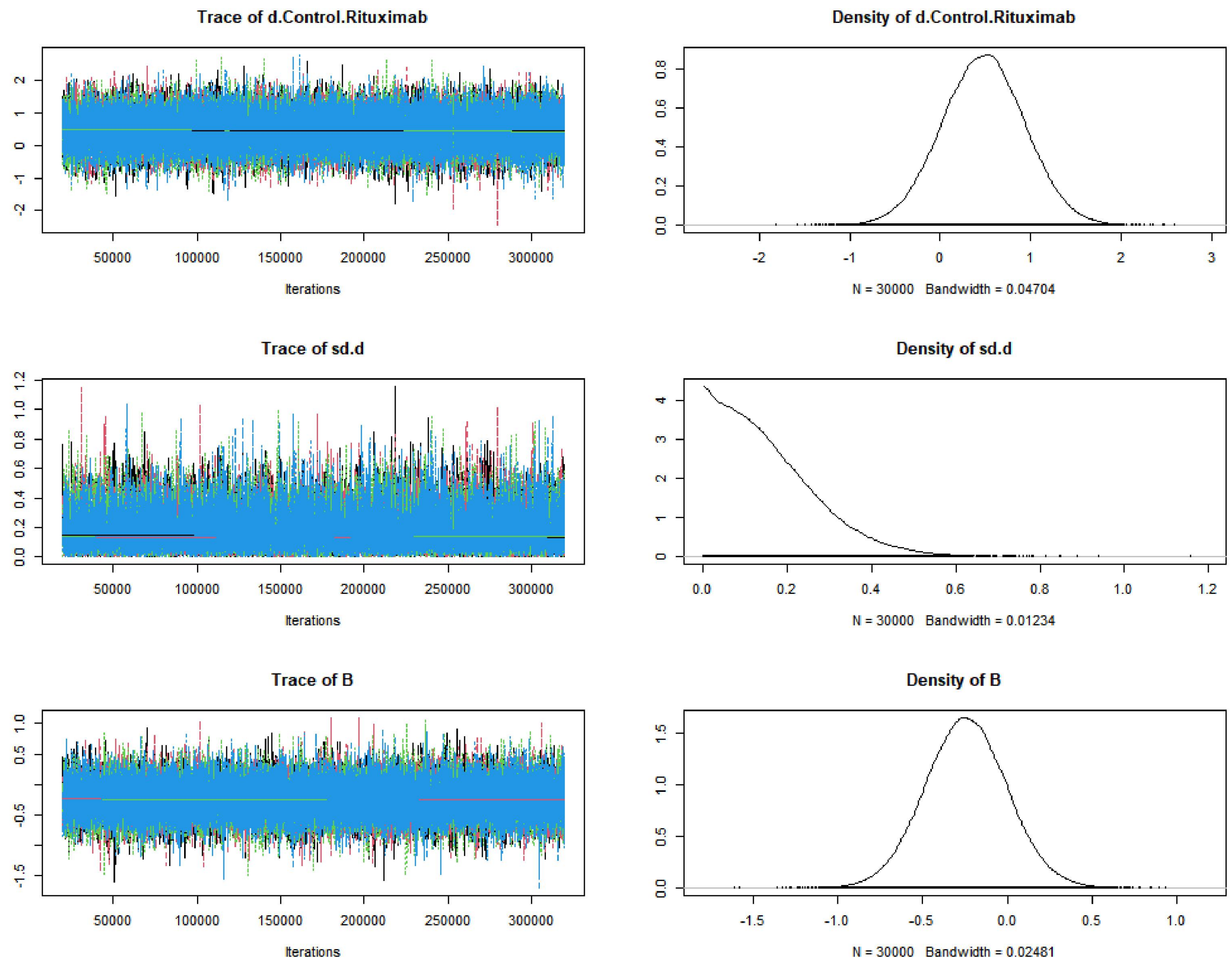

Supplementary Figure 11D. Traces plots and density plots of pairwise comparisons on PRR by Bayesian network meta-regression analyses.

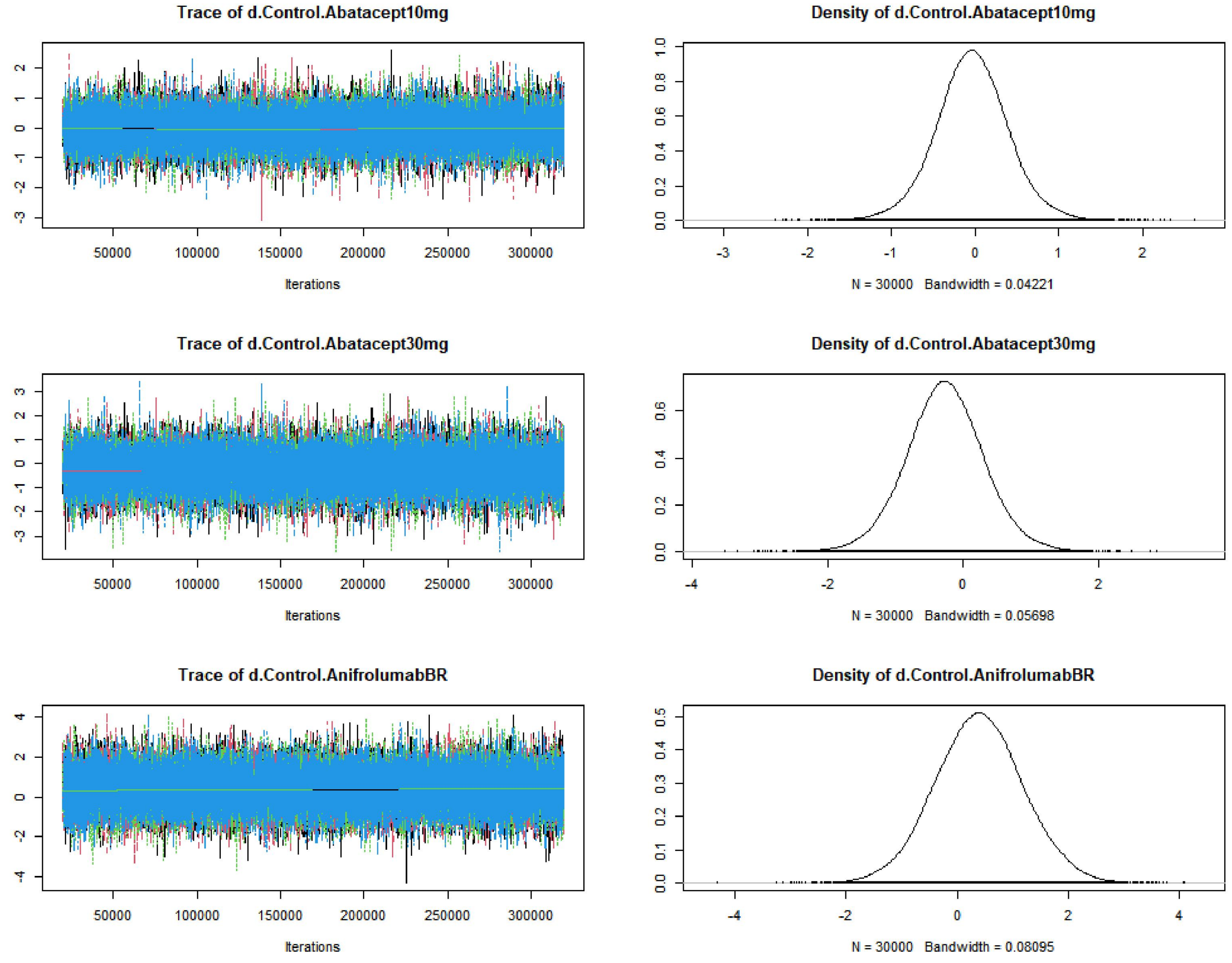

Supplementary Figure 12A. Traces plots and density plots of pairwise comparisons on AE by Bayesian network meta-regression analyses.

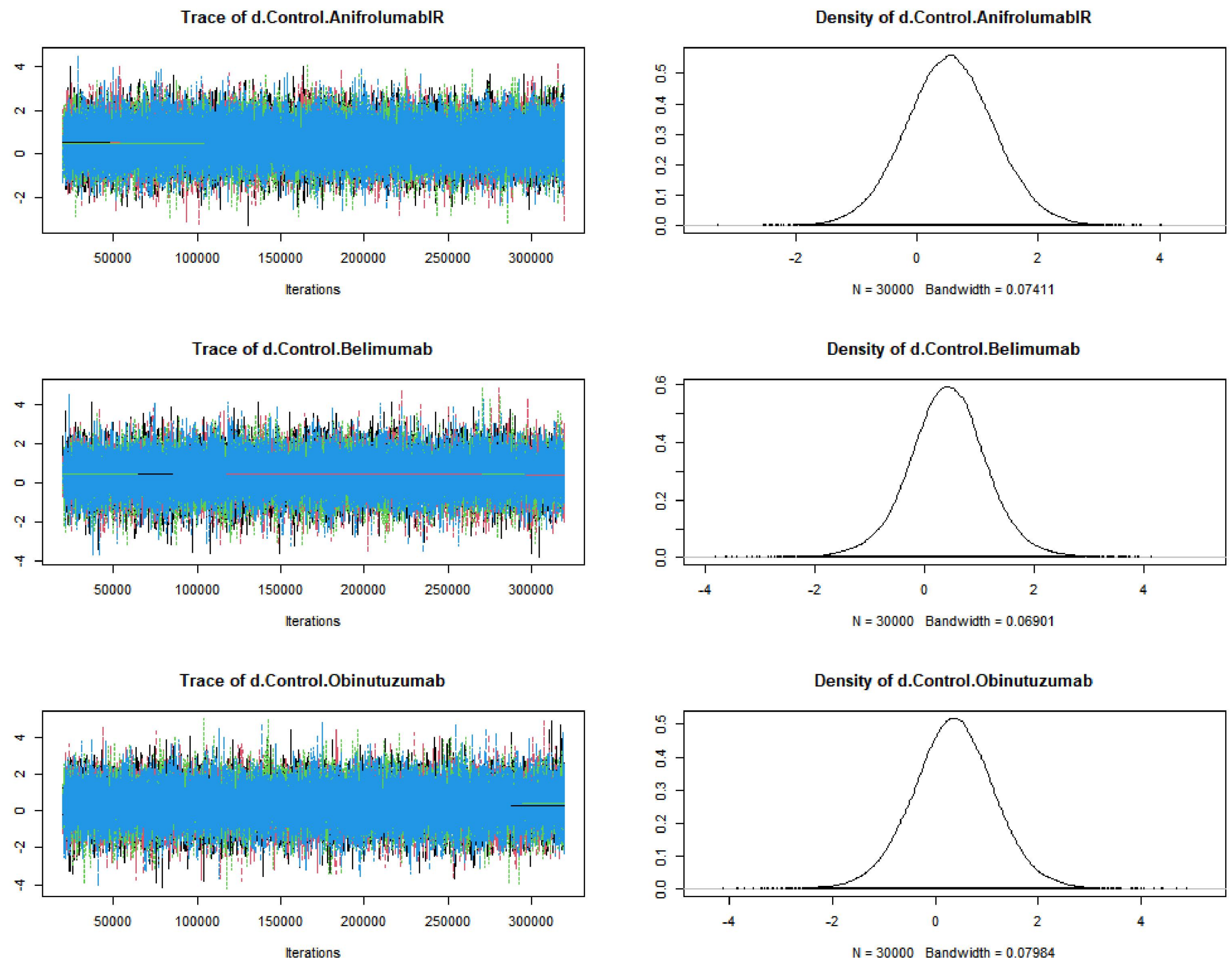

Supplementary Figure 12B. Traces plots and density plots of pairwise comparisons on AE by Bayesian network meta-regression analyses.

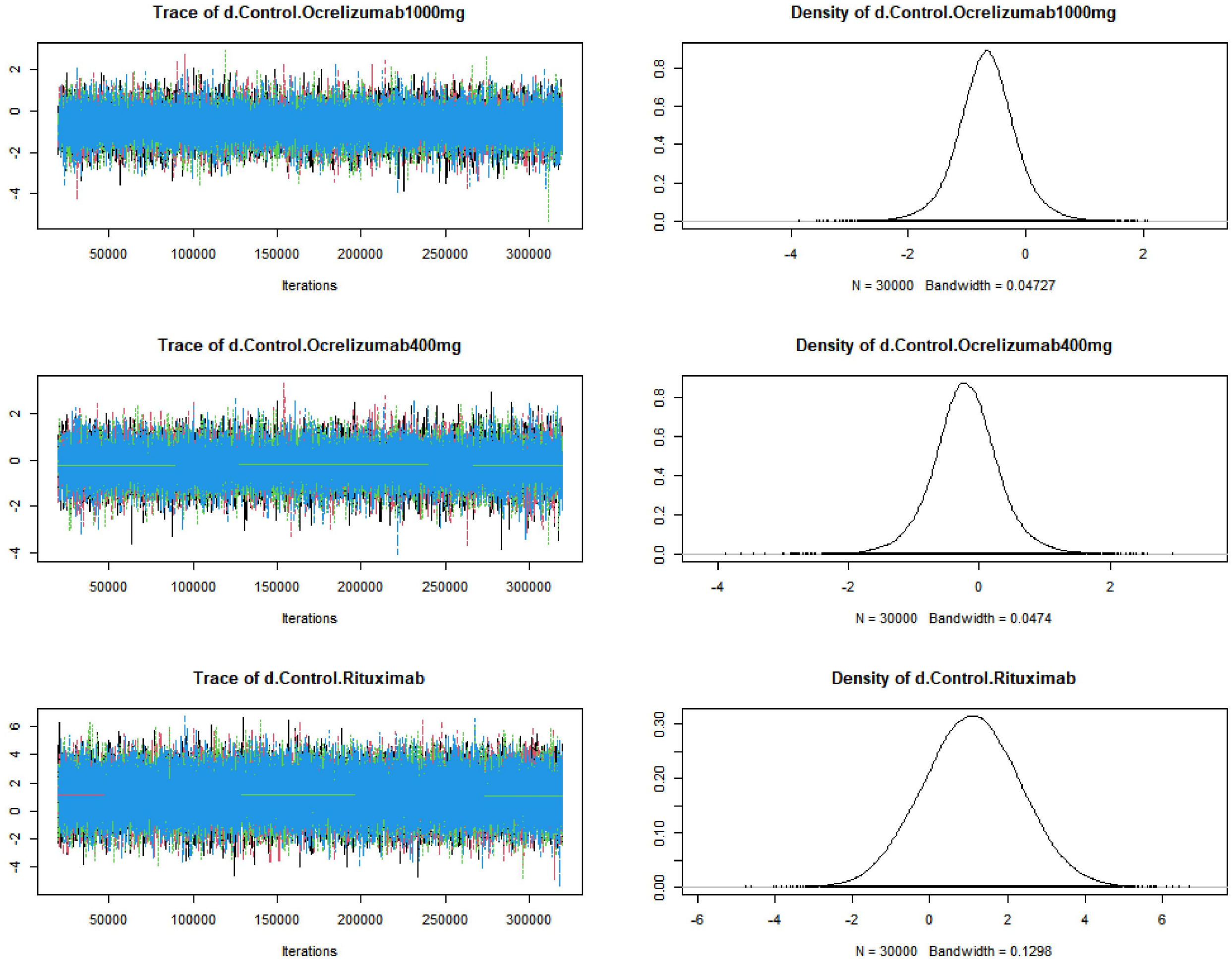

Supplementary Figure 12C. Traces plots and density plots of pairwise comparisons on AE by Bayesian network meta-regression analyses.

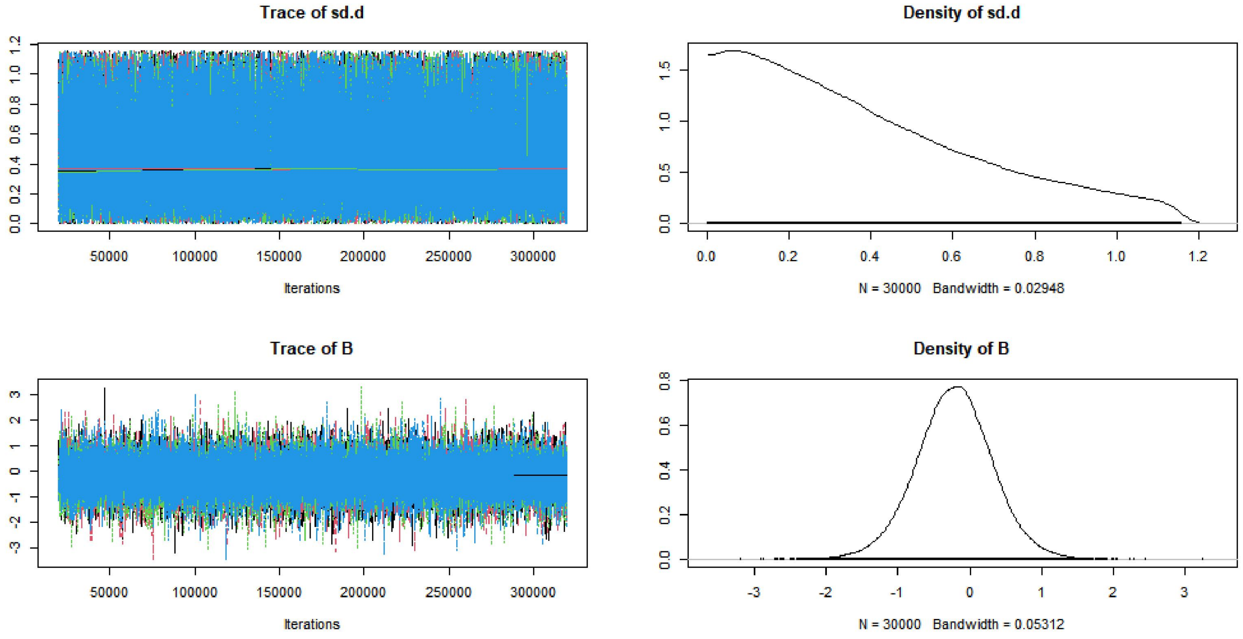

Supplementary Figure 12D. Traces plots and density plots of pairwise comparisons on AE by Bayesian network meta-regression analyses.

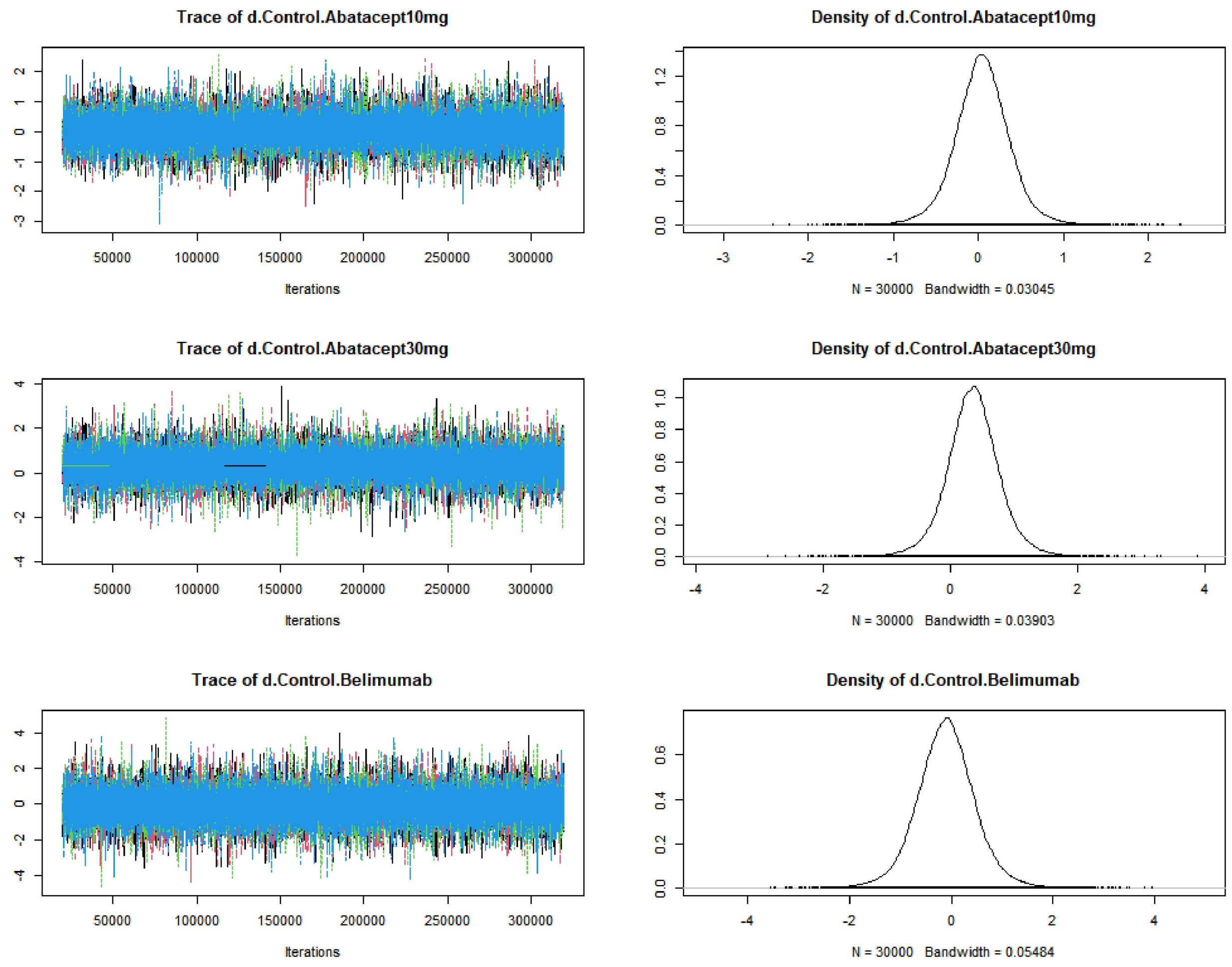

Supplementary Figure 13A. Traces plots and density plots of pairwise comparisons on IAE by Bayesian network meta-regression analyses.

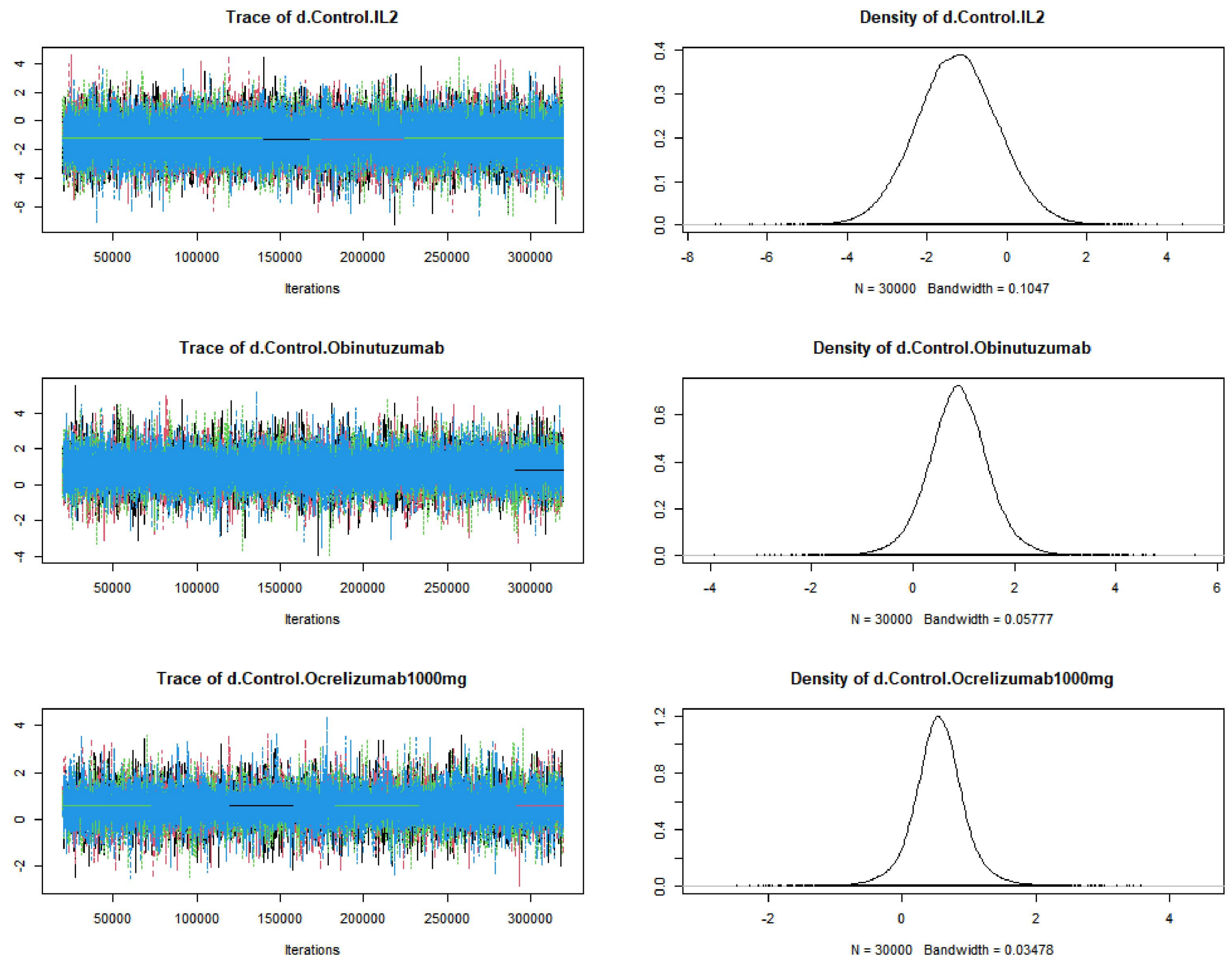

Supplementary Figure 13B. Traces plots and density plots of pairwise comparisons on IAE by Bayesian network meta-regression analyses.

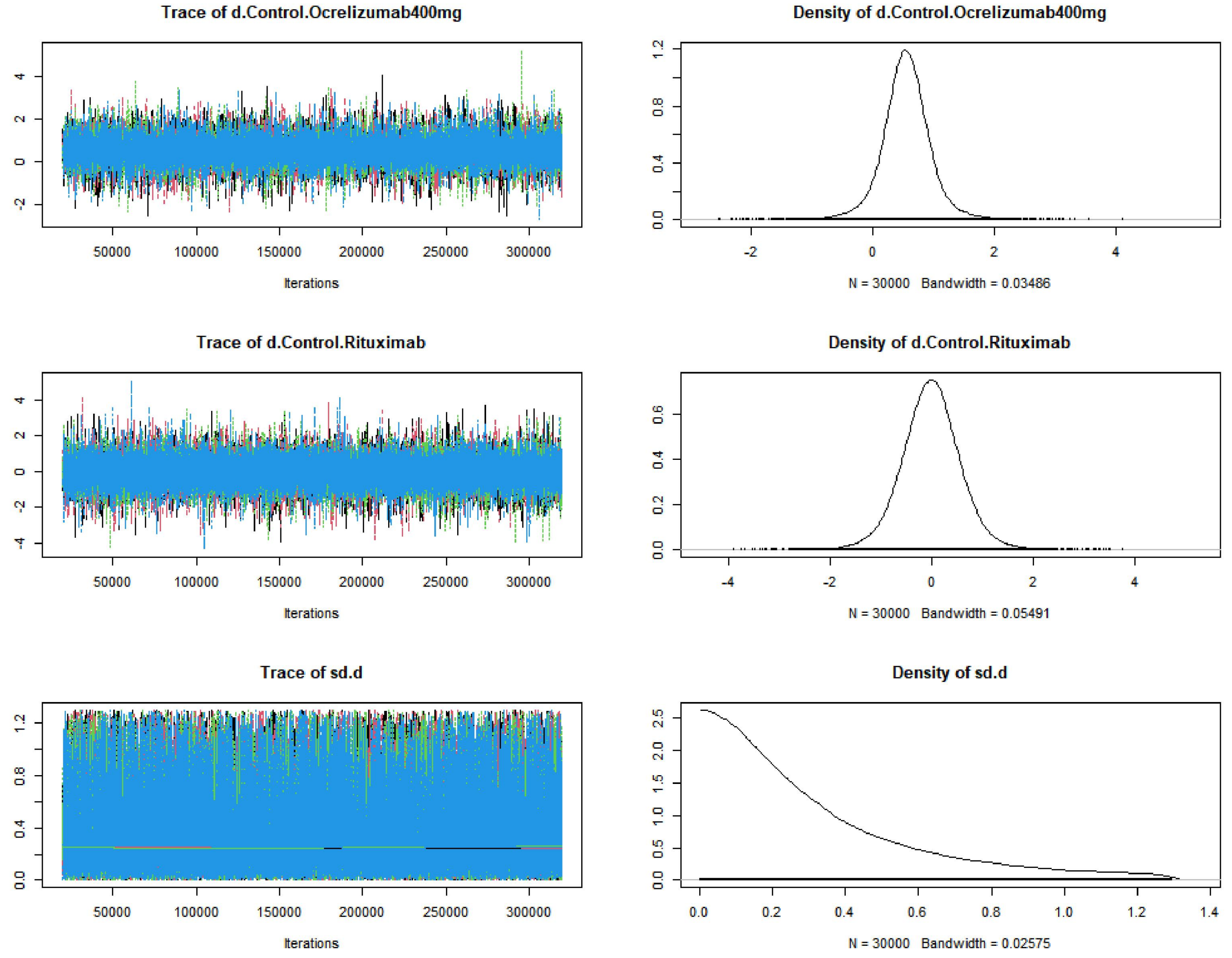

Supplementary Figure 143C. Convergence of the three Markov Chain Monte Carlo chains established by of the history feature for IAE.IAE:infection adverse event.

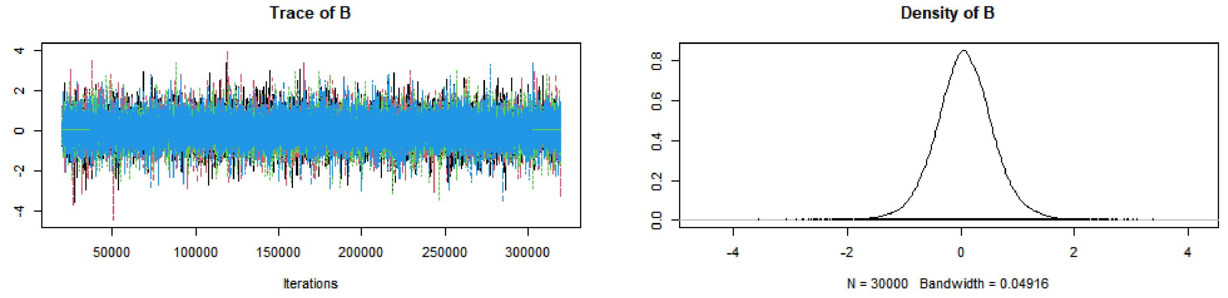

Supplementary Figure 13D. Traces plots and density plots of pairwise comparisons on IAE by Bayesian network meta-regression analyses.

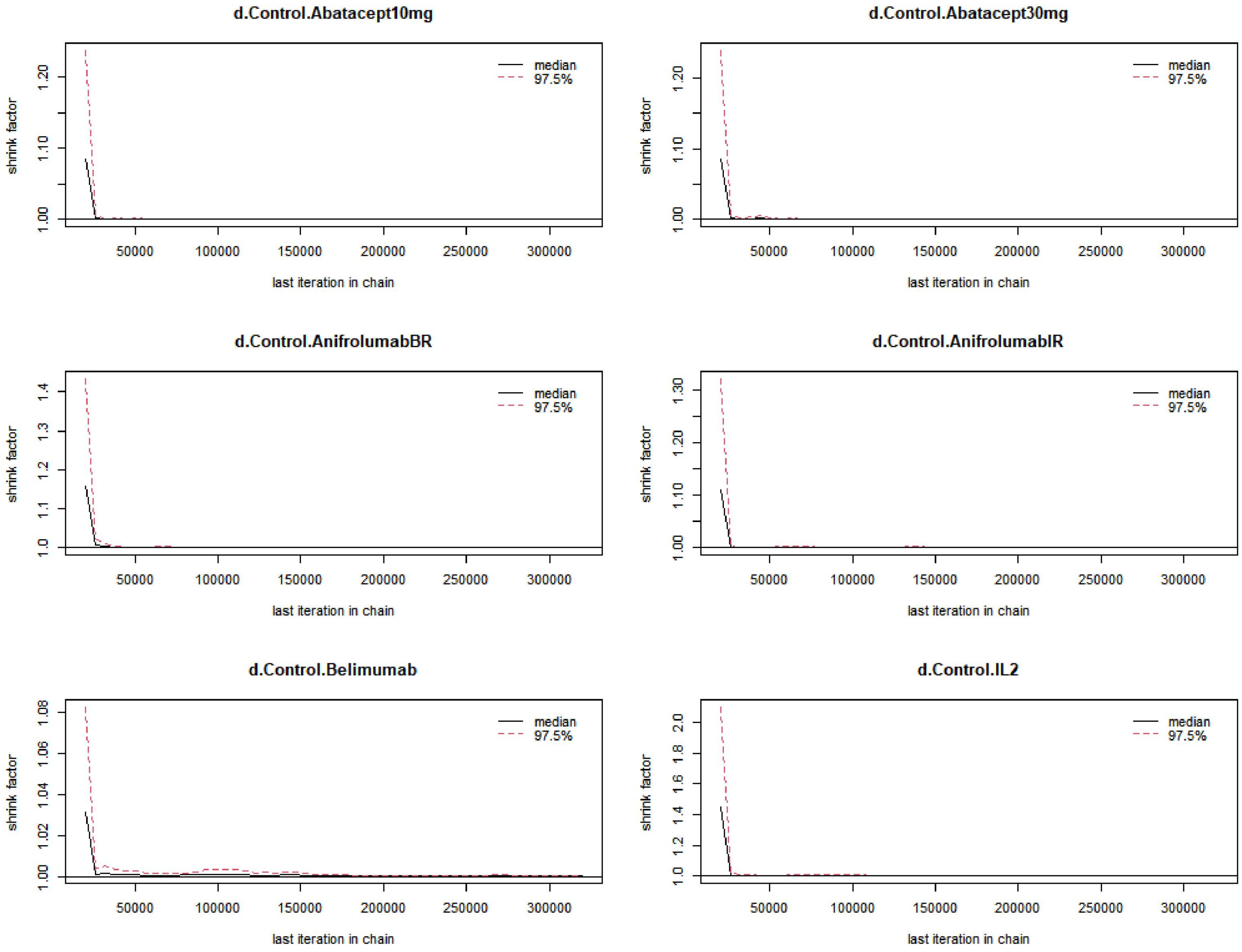

Supplementary Figure 14A. Brooks-Gelman-Rubin convergence diagnostic plots on CRR by Bayesian network meta-regression analyses.

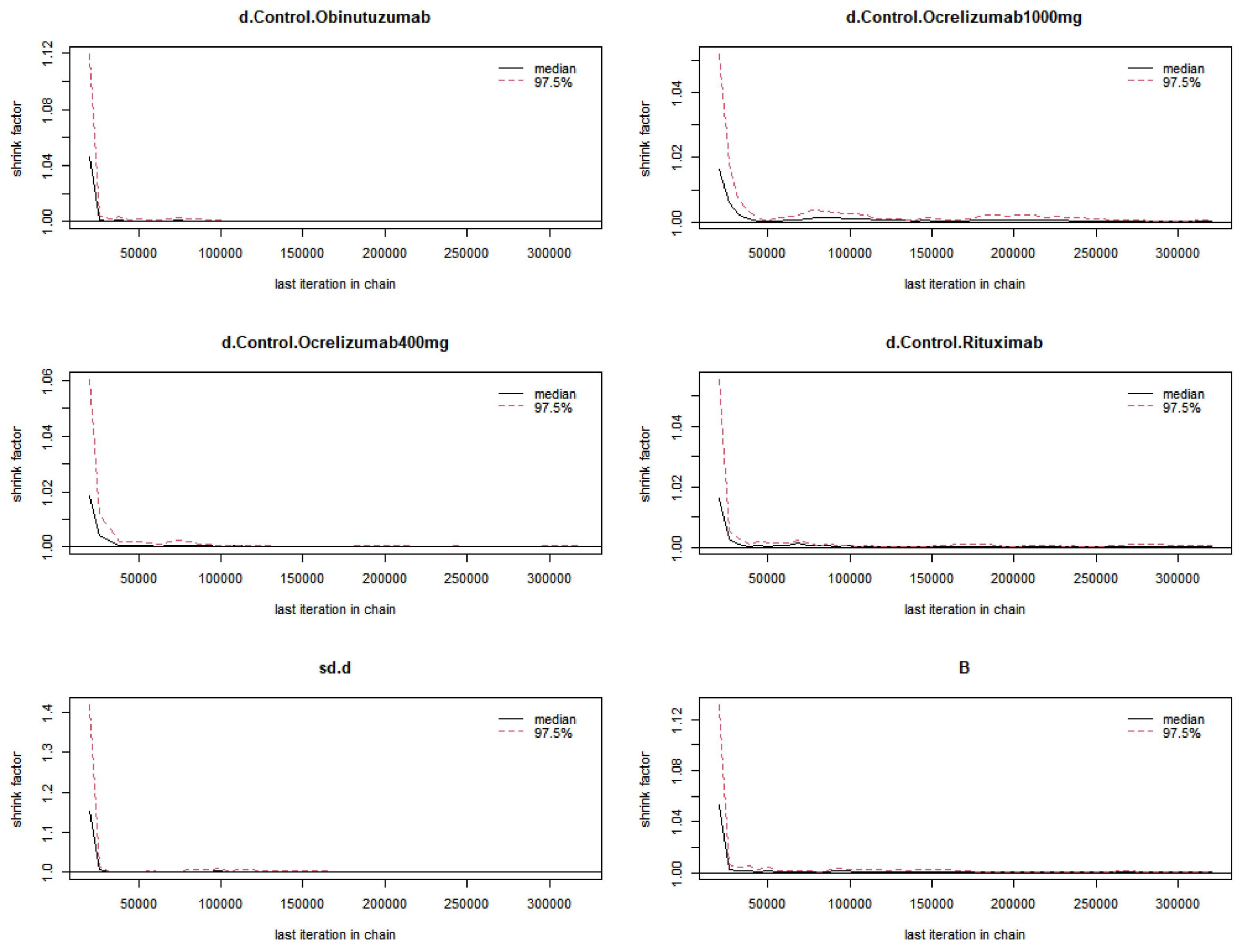

Supplementary Figure 14B. Brooks-Gelman-Rubin convergence diagnostic plots on CRR by Bayesian network meta-regression analyses.

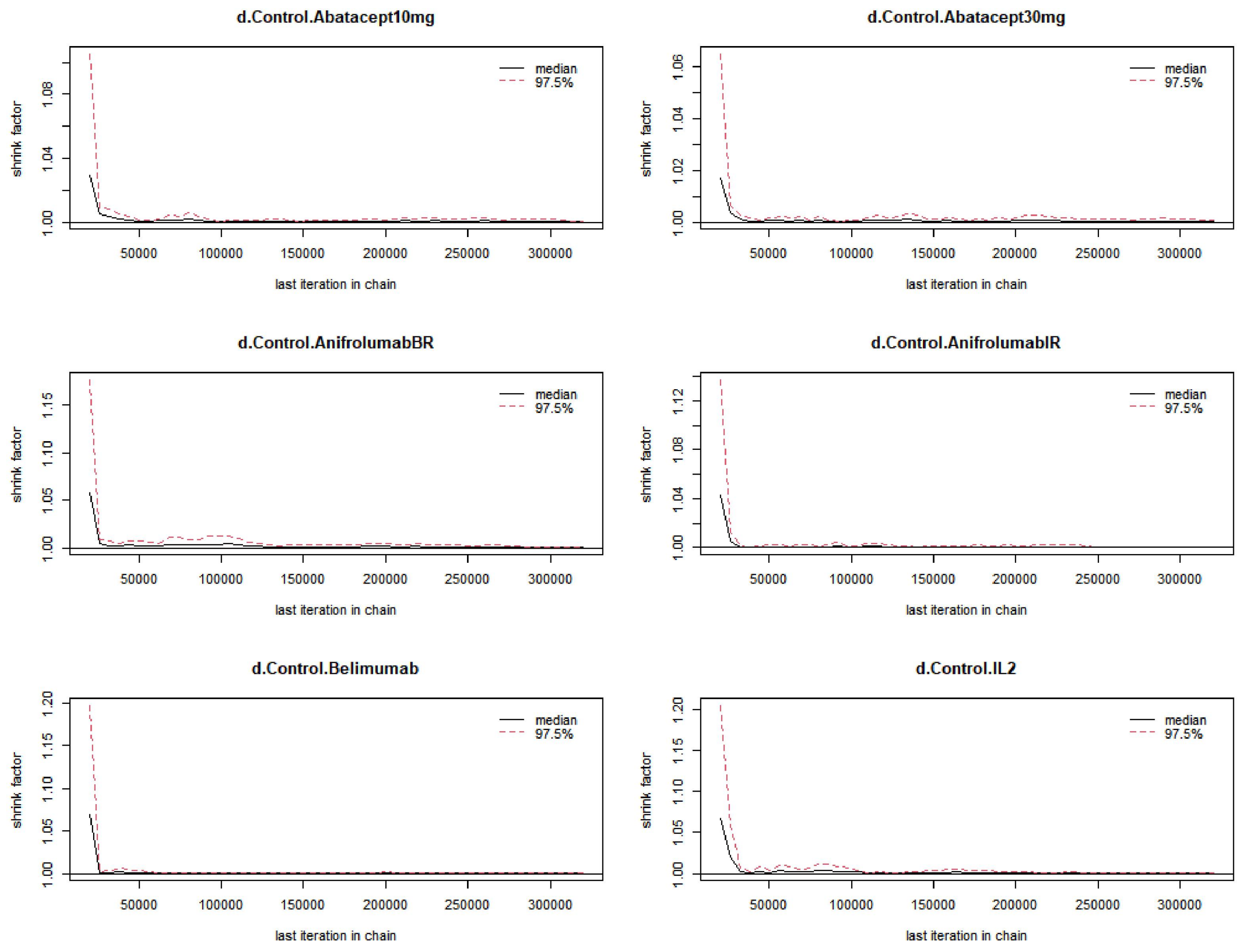

Supplementary Figure 15A. Brooks-Gelman-Rubin convergence diagnostic plots on PRR by Bayesian network meta-regression analyses.

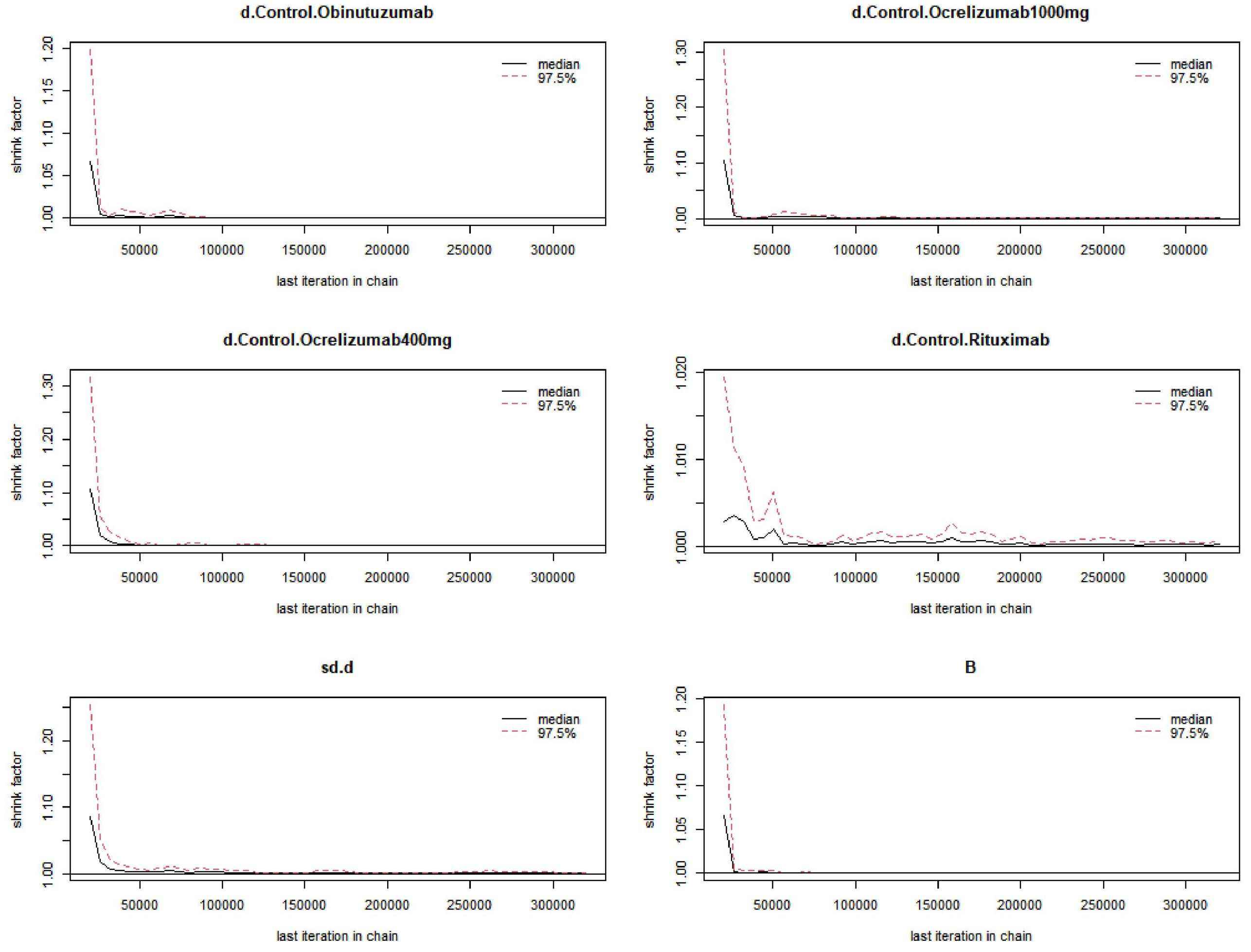

Supplementary Figure 15B. Brooks-Gelman-Rubin convergence diagnostic plots on PRR by Bayesian network meta-regression analyses.

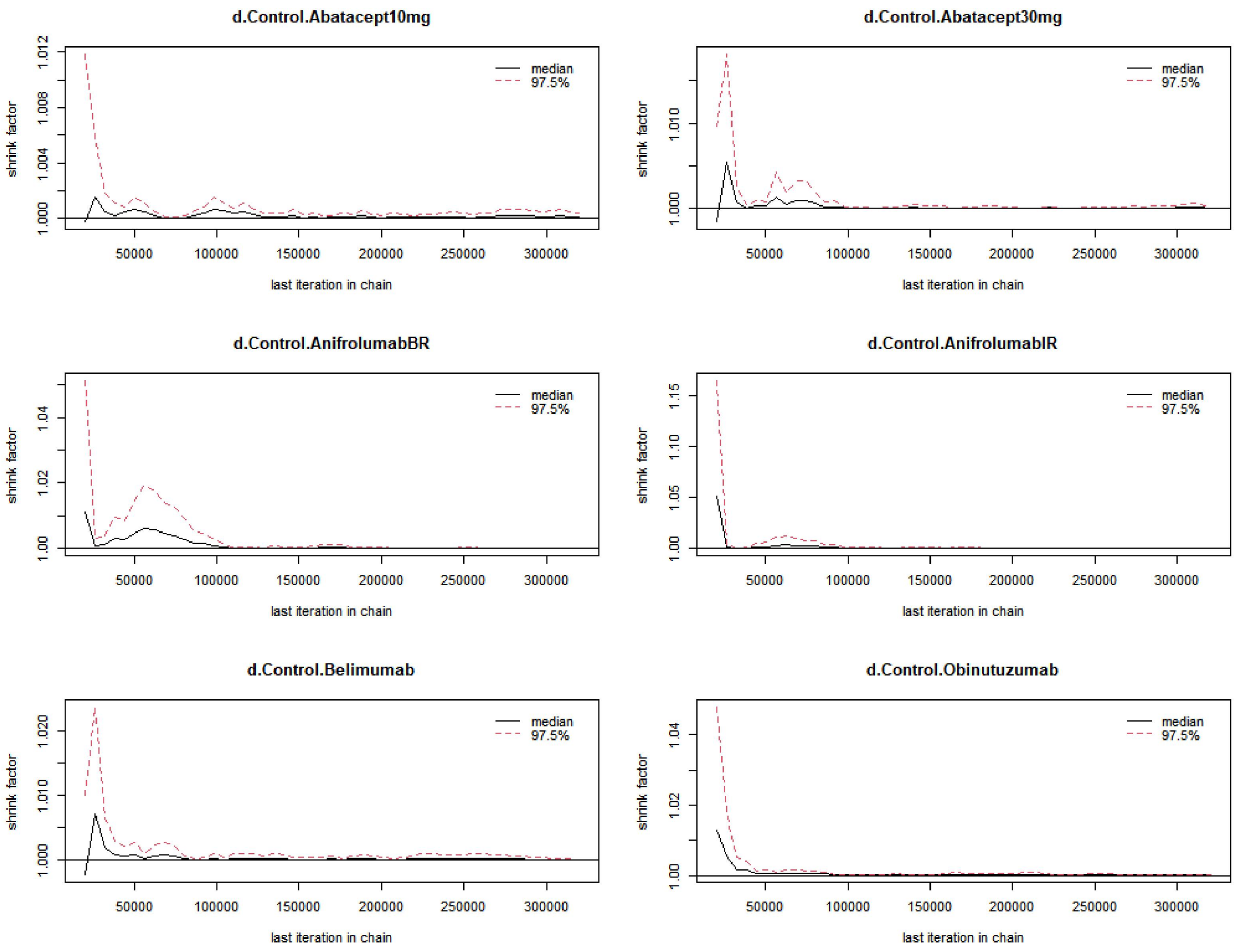

Supplementary Figure 16A. Brooks-Gelman-Rubin convergence diagnostic plots on AE by Bayesian network meta-regression analyses.

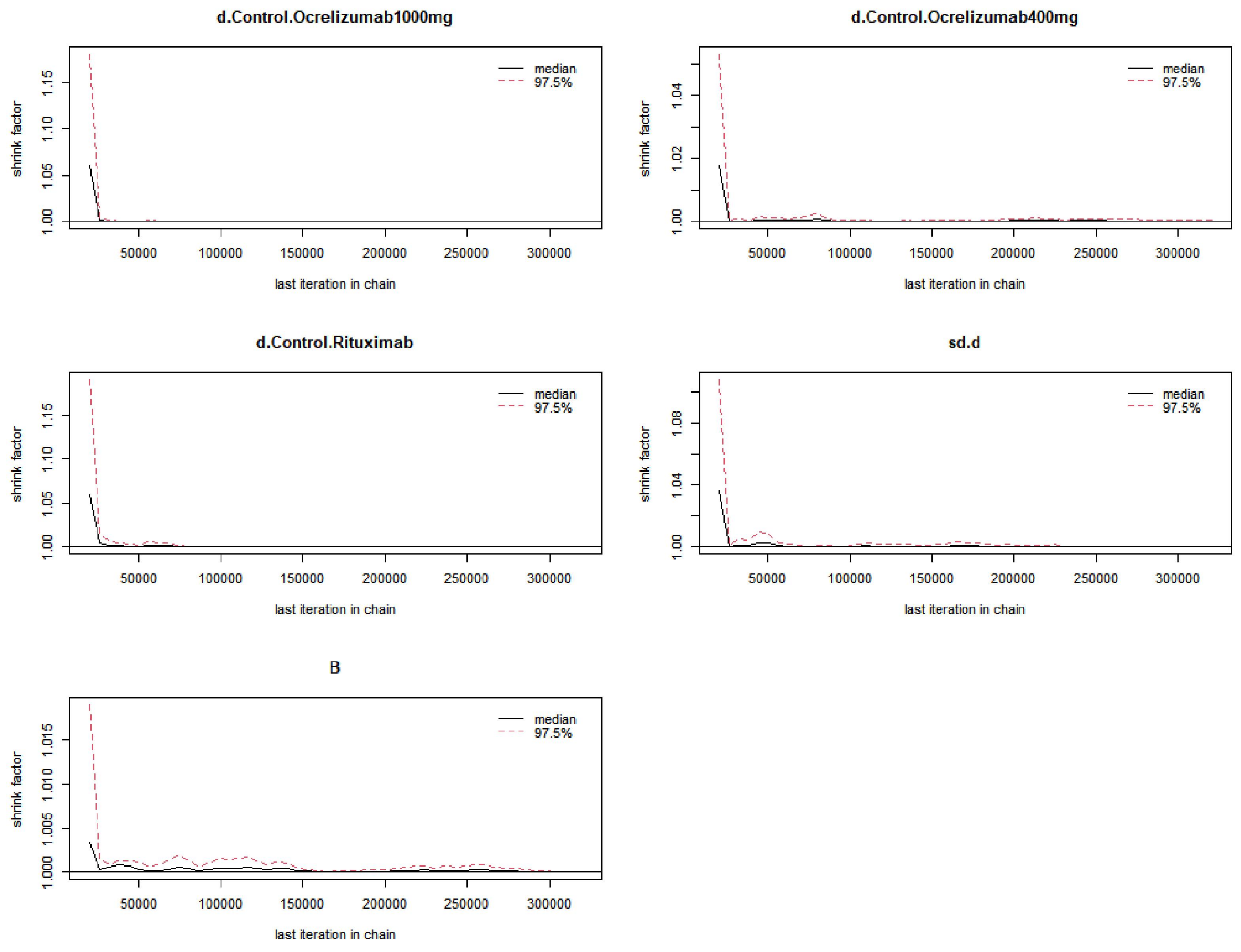

Supplementary Figure 16B. Brooks-Gelman-Rubin convergence diagnostic plots on AE by Bayesian network meta-regression analyses.

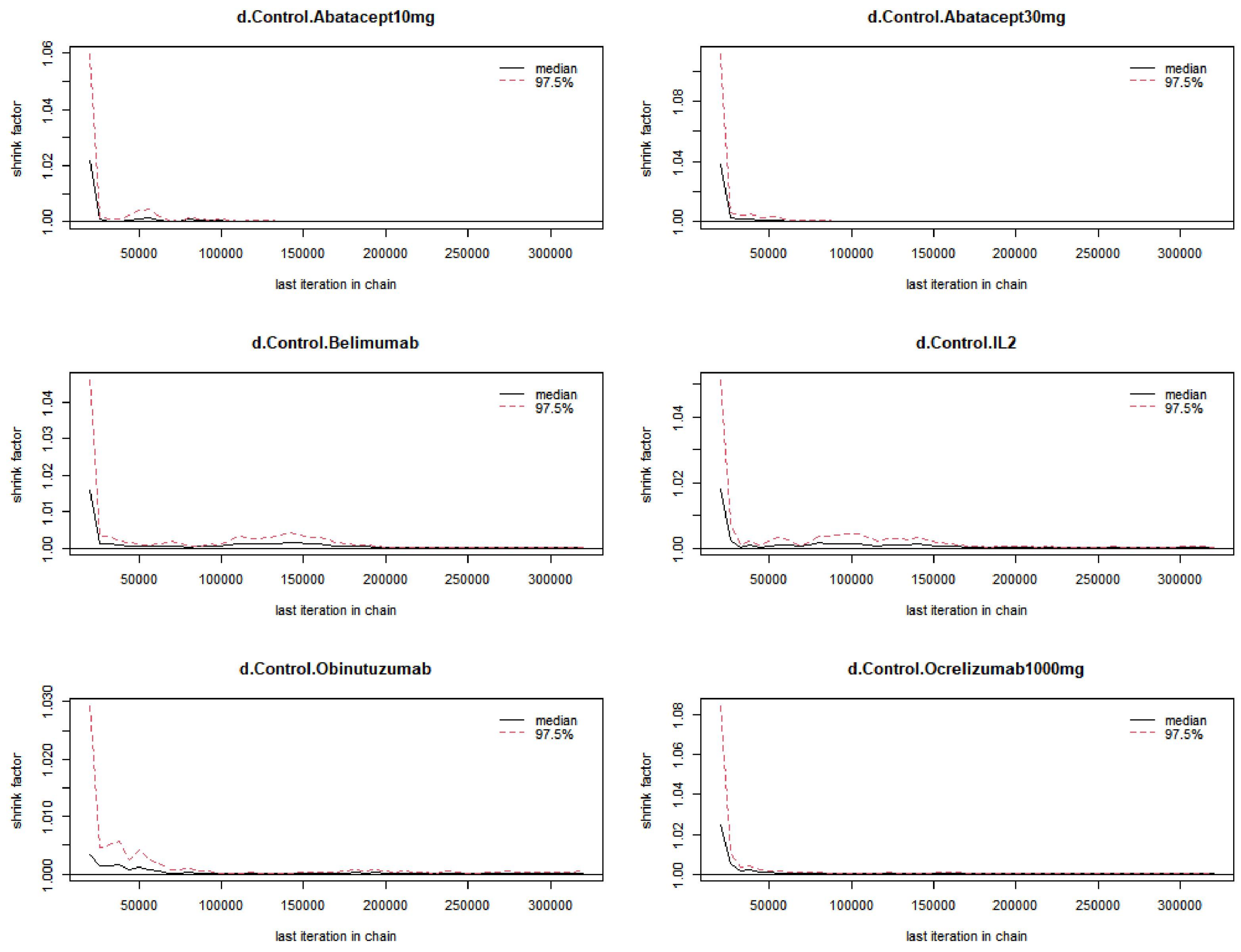

Supplementary Figure 17A. Brooks-Gelman-Rubin convergence diagnostic plots on IAE by Bayesian network meta-regression analyses.

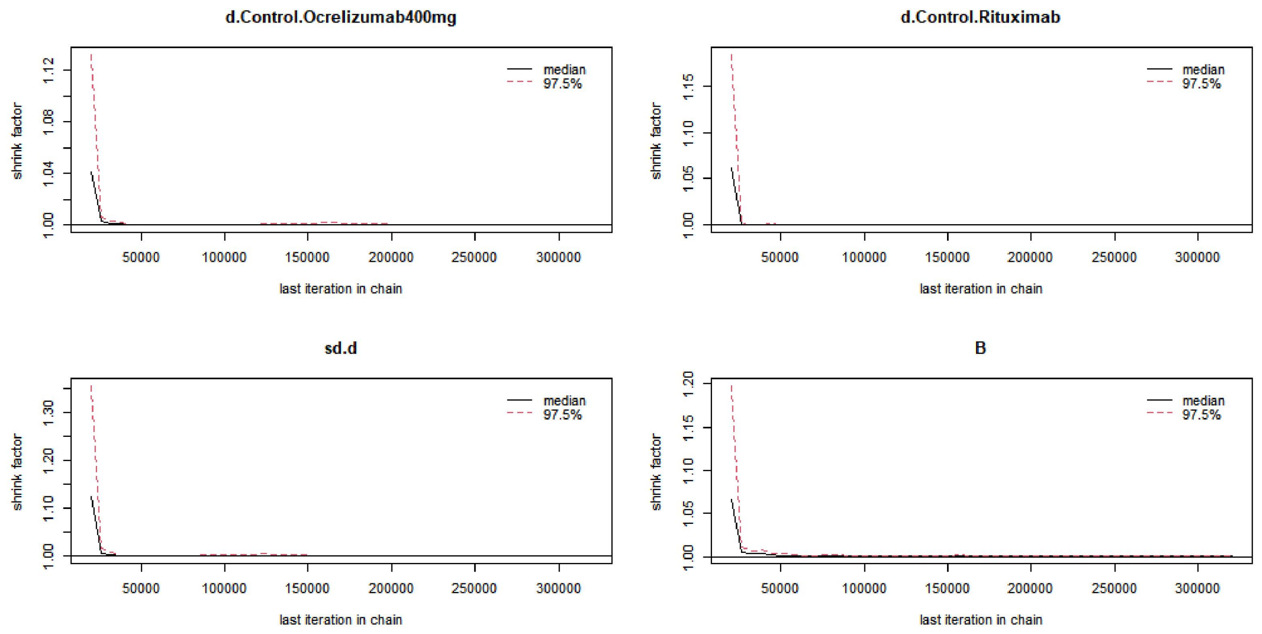

Supplementary Figure 17B. Brooks-Gelman-Rubin convergence diagnostic plots on IAE by Bayesian network meta-regression analyses.

2.2 Supplementary Tables

Supplementary Table 1. Matrix and SUCRA of pairwise comparisons of regimens on CRR utilizing Bayesian network meta analysis (shown as HR and 95% Cls).

	Abatacept-10mg	Abatacept-30mg	Anifrolumab-BR	Anifrolumab-IR	Belimumab
SUCRA (%)	59	69	3	58	63
Abatacept-10mg	Abatacept10mg	1.14 (0.73, 1.79)	0.29 (0.1, 0.78)	1 (0.37, 2.56)	1.07 (0.49, 2.16)
Abatacept-30mg	0.88 (0.56, 1.36)	Abatacept30mg	0.25 (0.09, 0.69)	0.87 (0.32, 2.3)	0.94 (0.41, 1.96)
Anifrolumab-BR	3.5 (1.29, 9.89)	3.99 (1.44, 11.63)	Anifrolumab-BR	3.49 (1.39, 8.81)	3.73 (1.22, 11.29)
Anifrolumab-IR	1 (0.39, 2.69)	1.15 (0.44, 3.17)	0.29 (0.11, 0.72)	Anifrolumab-IR	1.07 (0.37, 3.07)
Belimumab	0.93 (0.46, 2.05)	1.07 (0.51, 2.43)	0.27 (0.09, 0.82)	0.94 (0.33, 2.73)	Belimumab
Control	1.62 (1.1, 2.52)	1.85 (1.18, 3.08)	0.46 (0.18, 1.17)	1.61 (0.68, 3.86)	1.73 (0.93, 3.19)

IL2	0.28 (0.04, 2.07)	0.32 (0.04, 2.39)	0.08 (0.01, 0.69)	0.28 (0.03, 2.34)	0.3 (0.04, 2.3)
Obinutuzumab	0.76 (0.35, 1.77)	0.88 (0.39, 2.09)	0.22 (0.07, 0.7)	0.77 (0.25, 2.33)	0.82 (0.32, 2.06)
Ocrelizumab-1000mg	1.11 (0.5, 2.67)	1.28 (0.55, 3.16)	0.32 (0.1, 1.05)	1.12 (0.36, 3.46)	1.19 (0.46, 3.09)
Ocrelizumab-400mg	1.92 (0.85, 4.61)	2.19 (0.94, 5.45)	0.55 (0.17, 1.79)	1.91 (0.62, 6.01)	2.05 (0.78, 5.32)
Rituximab	1.42 (0.54, 4)	1.62 (0.6, 4.72)	0.41 (0.11, 1.49)	1.42 (0.4, 5.01)	1.52 (0.5, 4.58)

Supplementary Table 1. (Continue)

Control	IL2	Obinutuzumab	Ocrelizumab-1000mg	Ocrelizumab-400mg	Rituximab
24	91	75	52	19	36
0.62 (0.4, 0.91)	3.6 (0.48, 25.73)	1.31 (0.57, 2.84)	0.9 (0.37, 2)	0.52 (0.22, 1.18)	0.7 (0.25, 1.85)
0.54 (0.33, 0.85)	3.16 (0.42, 22.83)	1.14 (0.48, 2.57)	0.78 (0.32, 1.81)	0.46 (0.18, 1.06)	0.62 (0.21, 1.67)
2.16 (0.85, 5.46)	12.67 (1.46, 108.56)	4.56 (1.43, 14.5)	3.12 (0.96, 10.2)	1.82 (0.56, 5.98)	2.45 (0.67, 8.96)
0.62 (0.26, 1.47)	3.63 (0.43, 30.48)	1.31 (0.43, 3.96)	0.9 (0.29, 2.78)	0.52 (0.17, 1.62)	0.7 (0.2, 2.47)
0.58 (0.31, 1.08)	3.39 (0.43, 25.88)	1.22 (0.49, 3.09)	0.84 (0.32, 2.18)	0.49 (0.19, 1.27)	0.66 (0.22, 1.98)
Control	5.85 (0.83, 40.46)	2.11 (1.06, 4.21)	1.45 (0.7, 2.99)	0.84 (0.4, 1.75)	1.14 (0.46, 2.82)
0.17 (0.02, 1.21)	IL2	0.36 (0.05, 2.88)	0.25 (0.03, 1.99)	0.14 (0.02, 1.16)	0.19 (0.02, 1.66)
0.47 (0.24, 0.95)	2.78 (0.35, 21.85)	Obinutuzumab	0.69 (0.25, 1.87)	0.4 (0.15, 1.09)	0.54 (0.17, 1.69)
0.69 (0.33, 1.43)	4.06 (0.5, 32.21)	1.46 (0.54, 3.98)	Ocrelizumab400mg	0.58 (0.28, 1.21)	0.79 (0.24, 2.52)
1.19 (0.57, 2.47)	6.94 (0.86, 55.07)	2.51 (0.91, 6.89)	1.72 (0.83, 3.56)	Ocrelizumab1000mg	1.35 (0.42, 4.32)
0.88 (0.35, 2.19)	5.17 (0.6, 43.58)	1.86 (0.59, 5.83)	1.27 (0.4, 4.08)	0.74 (0.23, 2.39)	Rituximab

Supplementary Table 2. Matrix and SUCRA of pairwise comparisons of regimens on PRR utilizing Bayesian network meta analysis (shown as HR and 95% Cls).

	Abatacept-10mg	Abatacept-30mg	Anifrolumab-BR	Anifrolumab-IR	Belimumab
SUCRA (%)	59	69	3	58	63
Abatacept-10mg	Abatacept10mg	1.14 (0.73, 1.79)	0.29 (0.1, 0.78)	1 (0.37, 2.56)	1.07 (0.49, 2.16)
Abatacept-30mg	0.88 (0.56, 1.36)	Abatacept30mg	0.25 (0.09, 0.69)	0.87 (0.32, 2.3)	0.94 (0.41, 1.96)
Anifrolumab-BR	3.5 (1.29, 9.89)	3.99 (1.44, 11.63)	Anifrolumab-BR	3.49 (1.39, 8.81)	3.73 (1.22, 11.29)
Anifrolumab-IR	1 (0.39, 2.69)	1.15 (0.44, 3.17)	0.29 (0.11, 0.72)	Anifrolumab-IR	1.07 (0.37, 3.07)
Belimumab	0.93 (0.46, 2.05)	1.07 (0.51, 2.43)	0.27 (0.09, 0.82)	0.94 (0.33, 2.73)	Belimumab
Control	1.62 (1.1, 2.52)	1.85 (1.18, 3.08)	0.46 (0.18, 1.17)	1.61 (0.68, 3.86)	1.73 (0.93, 3.19)
IL2	0.28 (0.04, 2.07)	0.32 (0.04, 2.39)	0.08 (0.01, 0.69)	0.28 (0.03, 2.34)	0.3 (0.04, 2.3)
Obinutuzumab	0.76 (0.35, 1.77)	0.88 (0.39, 2.09)	0.22 (0.07, 0.7)	0.77 (0.25, 2.33)	0.82 (0.32, 2.06)
Ocrelizumab-1000mg	1.11 (0.5, 2.67)	1.28 (0.55, 3.16)	0.32 (0.1, 1.05)	1.12 (0.36, 3.46)	1.19 (0.46, 3.09)
Ocrelizumab-400mg	1.92 (0.85, 4.61)	2.19 (0.94, 5.45)	0.55 (0.17, 1.79)	1.91 (0.62, 6.01)	2.05 (0.78, 5.32)
Rituximab	1.42 (0.54, 4)	1.62 (0.6, 4.72)	0.41 (0.11, 1.49)	1.42 (0.4, 5.01)	1.52 (0.5, 4.58)

Supplementary Table 2. (Continue)

Control	IL2	Obinutuzumab	Ocrelizumab-1000mg	Ocrelizumab-400mg	Rituximab
24	91	75	52	19	36
0.62 (0.4, 0.91)	3.6 (0.48, 25.73)	1.31 (0.57, 2.84)	0.9 (0.37, 2)	0.52 (0.22, 1.18)	0.7 (0.25, 1.85)
0.54 (0.33, 0.85)	3.16 (0.42, 22.83)	1.14 (0.48, 2.57)	0.78 (0.32, 1.81)	0.46 (0.18, 1.06)	0.62 (0.21, 1.67)
2.16 (0.85, 5.46)	12.67 (1.46, 108.56)	4.56 (1.43, 14.5)	3.12 (0.96, 10.2)	1.82 (0.56, 5.98)	2.45 (0.67, 8.96)
0.62 (0.26, 1.47)	3.63 (0.43, 30.48)	1.31 (0.43, 3.96)	0.9 (0.29, 2.78)	0.52 (0.17, 1.62)	0.7 (0.2, 2.47)

0.58 (0.31, 1.08)	3.39 (0.43, 25.88)	1.22 (0.49, 3.09)	0.84 (0.32, 2.18)	0.49 (0.19, 1.27)	0.66 (0.22, 1.98)
Control	5.85 (0.83, 40.46)	2.11 (1.06, 4.21)	1.45 (0.7, 2.99)	0.84 (0.4, 1.75)	1.14 (0.46, 2.82)
0.17 (0.02, 1.21)	IL2	0.36 (0.05, 2.88)	0.25 (0.03, 1.99)	0.14 (0.02, 1.16)	0.19 (0.02, 1.66)
0.47 (0.24, 0.95)	2.78 (0.35, 21.85)	Obinutuzumab	0.69 (0.25, 1.87)	0.4 (0.15, 1.09)	0.54 (0.17, 1.69)
0.69 (0.33, 1.43)	4.06 (0.5, 32.21)	1.46 (0.54, 3.98)	Ocrelizumab400mg	0.58 (0.28, 1.21)	0.79 (0.24, 2.52)
1.19 (0.57, 2.47)	6.94 (0.86, 55.07)	2.51 (0.91, 6.89)	1.72 (0.83, 3.56)	Ocrelizumab1000mg	1.35 (0.42, 4.32)
0.88 (0.35, 2.19)	5.17 (0.6, 43.58)	1.86 (0.59, 5.83)	1.27 (0.4, 4.08)	0.74 (0.23, 2.39)	Rituximab

Supplementary Table 3. Matrix and SUCRA of pairwise comparisons of regimens on AE utilizing Bayesian network meta analysis (shown as HR and 95% Cls).

	Control	Obinutuzumab	Rituximab	Anifrolumab-BR	Anifrolumab-IR
SUCRA (%)	46	56	79	64	71
Control	Control	1.25 (0.29, 5.41)	3.17 (0.27, 36.88)	1.58 (0.34, 7.29)	1.82 (0.45, 7.52)
Obinutuzumab	0.8 (0.18, 3.42)	Obinutuzumab	2.53 (0.15, 43.85)	1.26 (0.15, 10.41)	1.45 (0.19, 11.14)
Rituximab	0.32 (0.03, 3.64)	0.4 (0.02, 6.85)	Rituximab	0.5 (0.03, 8.93)	0.58 (0.03, 9.59)
Anifrolumab-BR	0.63 (0.14, 2.93)	0.79 (0.1, 6.5)	2.02 (0.11, 35.82)	Anifrolumab-BR	1.15 (0.25, 5.41)
Anifrolumab-IR	0.55 (0.13, 2.23)	0.69 (0.09, 5.18)	1.73 (0.1, 29.39)	0.87 (0.18, 4.04)	Anifrolumab-IR
Belimumab	0.74 (0.21, 2.62)	0.93 (0.13, 6.4)	2.34 (0.15, 36.91)	1.17 (0.16, 8.5)	1.34 (0.2, 8.97)
Abatacep-t30mg	1.38 (0.46, 4.17)	1.74 (0.28, 10.84)	4.39 (0.3, 64.76)	2.19 (0.33, 14.54)	2.51 (0.42, 15.07)
Abatacept-10mg	1.04 (0.44, 2.44)	1.3 (0.24, 7.08)	3.28 (0.25, 44.28)	1.64 (0.29, 9.4)	1.88 (0.37, 9.85)
Ocrelizumab-400mg	1.15 (0.44, 2.98)	1.44 (0.25, 8.18)	3.63 (0.26, 49.91)	1.82 (0.3, 10.91)	2.08 (0.39, 11.42)
Ocrelizumab-1000mg	1.8 (0.7, 4.63)	2.26 (0.4, 12.81)	5.69 (0.42, 78.52)	2.84 (0.47, 17.01)	3.28 (0.61, 17.86)

Supplementary Table 3. (Continue)

Belimumab	Abatacept-30mg	Abatacep-t10mg	Ocrelizumab-400mg	Ocrelizumab-1000mg
60	28	44	38	14
1.35 (0.38, 4.8)	0.72 (0.24, 2.19)	0.97 (0.41, 2.26)	0.87 (0.34, 2.27)	0.56 (0.22, 1.44)
1.08 (0.16, 7.45)	0.57 (0.09, 3.61)	0.77 (0.14, 4.15)	0.7 (0.12, 3.96)	0.44 (0.08, 2.5)
0.43 (0.03, 6.71)	0.23 (0.02, 3.38)	0.3 (0.02, 4.05)	0.28 (0.02, 3.79)	0.18 (0.01, 2.4)
0.86 (0.12, 6.21)	0.46 (0.07, 3.05)	0.61 (0.11, 3.5)	0.55 (0.09, 3.34)	0.35 (0.06, 2.12)
0.75 (0.11, 4.91)	0.4 (0.07, 2.37)	0.53 (0.1, 2.73)	0.48 (0.09, 2.58)	0.31 (0.06, 1.64)
Belimumab	0.54 (0.1, 2.88)	0.71 (0.16, 3.28)	0.64 (0.13, 3.15)	0.41 (0.08, 1.99)
1.87 (0.35, 9.96)	Abatacept-30mg	1.33 (0.45, 3.93)	1.21 (0.28, 5.19)	0.77 (0.18, 3.29)
1.4 (0.31, 6.45)	0.75 (0.25, 2.24)	Abatacept-10mg	0.9 (0.25, 3.27)	0.58 (0.16, 2.06)
1.55 (0.32, 7.58)	0.83 (0.19, 3.57)	1.11 (0.31, 3.97)	Ocrelizumab-400mg	0.64 (0.25, 1.64)
2.43 (0.5, 11.81)	1.3 (0.3, 5.57)	1.74 (0.48, 6.19)	1.57 (0.61, 4.03)	Ocrelizumab-1000mg

Supplementary Table 4. Matrix and SUCRA of pairwise comparisons of regimens on IAE utilizing Bayesian network meta analysis (shown as HR and 95% Cls).

	Control	Obinutuzumab	IL2	Rituximab	Belimumab
SUCRA (%)	34	88	7	38	35
Control	Control	2.53 (0.88, 7.28)	0.27 (0.04, 1.72)	0.97 (0.33, 2.84)	0.93 (0.34, 2.55)
Obinutuzumab	0.4 (0.14, 1.13)	Obinutuzumab	0.11 (0.01, 0.9)	0.38 (0.08, 1.73)	0.37 (0.09, 1.59)
IL2	3.65 (0.58, 22.52)	9.22 (1.12, 76.5)	IL2	3.55 (0.42, 29.31)	3.42 (0.42, 27.17)
Rituximab	1.03 (0.35, 3.04)	2.61 (0.58, 11.82)	0.28 (0.03, 2.39)	Rituximab	0.97 (0.22, 4.2)
Belimumab	1.07 (0.39, 2.92)	2.7 (0.63, 11.69)	0.29 (0.04, 2.37)	1.04 (0.24, 4.54)	Belimumab

Abatacep-t30mg	0.69 (0.32, 1.48)	1.74 (0.47, 6.44)	0.19 (0.03, 1.37)	0.67 (0.18, 2.5)	0.64 (0.18, 2.28)
Abatacept-10mg	0.95 (0.51, 1.79)	2.41 (0.71, 8.29)	0.26 (0.04, 1.81)	0.92 (0.27, 3.22)	0.89 (0.27, 2.91)
Ocrelizumab-400mg	0.59 (0.29, 1.2)	1.48 (0.42, 5.28)	0.16 (0.02, 1.14)	0.57 (0.16, 2.06)	0.55 (0.16, 1.87)
Ocrelizumab-1000mg	0.59 (0.29, 1.19)	1.48 (0.42, 5.25)	0.16 (0.02, 1.14)	0.57 (0.15, 2.06)	0.55 (0.16, 1.88)

Supplementary Table 3. (Continue)

Abatacept-30mg	Abatacept-10mg	Ocrelizumab-400mg	Ocrelizumab-1000mg
63	39	73	74
1.45 (0.68, 3.12)	1.05 (0.56, 1.96)	1.71 (0.84, 3.5)	1.71 (0.84, 3.5)
0.57 (0.16, 2.11)	0.41 (0.12, 1.41)	0.68 (0.19, 2.4)	0.67 (0.19, 2.4)
5.28 (0.73, 38.26)	3.82 (0.55, 26.26)	6.22 (0.87, 43.79)	6.22 (0.87, 43.72)
1.49 (0.4, 5.65)	1.08 (0.31, 3.76)	1.76 (0.49, 6.42)	1.76 (0.48, 6.46)
1.55 (0.44, 5.52)	1.12 (0.34, 3.67)	1.83 (0.53, 6.3)	1.83 (0.53, 6.29)
Abatacept-30mg	0.72 (0.34, 1.55)	1.18 (0.41, 3.35)	1.18 (0.42, 3.35)
1.38 (0.65, 2.97)	Abatacept-10mg	1.63 (0.63, 4.2)	1.63 (0.63, 4.19)
0.85 (0.3, 2.42)	0.61 (0.24, 1.58)	Ocrelizumab-400mg	1 (0.49, 2.05)
0.85 (0.3, 2.41)	0.61 (0.24, 1.58)	1 (0.49, 2.05)	Ocrelizumab-1000mg
